# Urinary CE-MS peptide marker pattern for detection of solid tumors

**DOI:** 10.1038/s41598-018-23585-y

**Published:** 2018-03-27

**Authors:** Iwona Belczacka, Agnieszka Latosinska, Justyna Siwy, Jochen Metzger, Axel S. Merseburger, Harald Mischak, Antonia Vlahou, Maria Frantzi, Vera Jankowski

**Affiliations:** 1 Mosaiques Diagnostics GmbH, Hannover, Germany; 2 University Hospital RWTH Aachen, Institute for Molecular Cardiovascular Research (IMCAR), Aachen, Germany; 3 Department of Urology, University of Lübeck, Lübeck, Germany; 4 University of Glasgow, Institute of Cardiovascular and Medical Sciences, Glasgow, United Kingdom; 5 Biotechnology Division, Biomedical Research Foundation, Academy of Athens (BRFAA), Athens, Greece

**Keywords:** Metastasis, Tumour biomarkers

## Abstract

Urinary profiling datasets, previously acquired by capillary electrophoresis coupled to mass-spectrometry were investigated to identify a general urinary marker pattern for detection of solid tumors by targeting common systemic events associated with tumor-related inflammation. A total of 2,055 urinary profiles were analyzed, derived from a) a cancer group of patients (n = 969) with bladder, prostate, and pancreatic cancers, renal cell carcinoma, and cholangiocarcinoma and b) a control group of patients with benign diseases (n = 556), inflammatory diseases (n = 199) and healthy individuals (n = 331). Statistical analysis was conducted in a discovery set of 676 cancer cases and 744 controls. 193 peptides differing at statistically significant levels between cases and controls were selected and combined to a multi-dimensional marker pattern using support vector machine algorithms. Independent validation in a set of 635 patients (293 cancer cases and 342 controls) showed an AUC of 0.82. Inclusion of age as independent variable, significantly increased the AUC value to 0.85. Among the identified peptides were mucins, fibrinogen and collagen fragments. Further studies are planned to assess the pattern value to monitor patients for tumor recurrence. In this proof-of-concept study, a general tumor marker pattern was developed to detect cancer based on shared biomarkers, likely indicative of cancer-related features.

## Introduction

Cancer is one of the leading causes of death worldwide, contributing to 21% of the overall mortality rate^1^. It is also the second leading disease condition affecting people in developing countries^1^. In 2012, 14.1 million newly diagnosed cancer cases and 8.2 million cancer-related deaths were recorded^1^. Moreover, by the year 2030, the global cancer incidence is predicted to be nearly doubled^2^. The major cause of cancer morbidity and mortality is cancer metastasis, accounting for approximately 90% of all cancer related deaths^3^. As the molecular mechanisms that trigger metastasis are still under investigation, it has been shown that cancer metastasis involves a series of pathological changes, starting from local invasion, followed by tumor cell intravasation towards lymphatic and blood vessels and subsequent extravasation to distant organs and tissues leading to secondary tumor formation. In addition, this complex process of human tumor pathogenesis is accompanied by intrinsic inflammatory responses^4^.

Despite the remarkable research progress and drug development, the low long-term survival rates of most cancer patients leaves room for improvement in the management of cancer^5^. A cornerstone is, on the one hand, timely detection, when curative treatment is still possible, thus the chances of positive outcome are higher. At the same time, there is also an increasing clinical need for monitoring of cancer relapse and response to treatment. The current diagnostic/monitoring approaches typically involve the combination of imaging techniques and invasive tissue biopsies^6^. For diagnostic screening, the generally preferred specimens are easily accessible body fluids, such as blood serum/plasma or urine due to the relatively non-invasive manner of collection^7,8^. In an effort to increase the chances of detecting cancer earlier, non-invasive screening tests have been introduced into clinical practice, such as: a) carcinoembryonic antigen (CEA)^9^, mainly used for monitoring of cholangiocarcinoma, b) cancer antigen 125 (CA-125)^10^ primarily used to monitor treatment of ovarian cancer, c) prostate specific antigen (PSA)^11^ used for the detection of prostate cancer and d) carbohydrate antigen 19-9 (CA19-9)^12^ mainly applied for the detection of pancreatic cancer.

Several proteomics studies involving capillary electrophoresis coupled to mass-spectrometry (CE-MS) particularly in the field of cancer^8,13–16^ and chronic diseases^17^ have demonstrated a good diagnostic potential of urinary peptide biomarkers, when combined to multi-marker patterns. These biomarkers have been identified in the context of a single type of cancer (e.g. bladder, prostate, pancreatic, renal cell carcinoma, cholangiocarcinoma etc.^8,13–16^).

In this proof-of-concept study, the main objective was to investigate pre-existing CE-MS urinary profiling data from different tumors, aiming at the identification of cancer-related markers in urine. The research hypothesis was that cancer progression is associated with common systemic molecular changes, likely reflecting tumor invasion and inflammation. Based on the observation that at least in part, these changes are detectable in urine, we further hypothesized that a combination of such urinary peptides into a multi-marker pattern, may serve as a non-invasive test to monitor cancer progression and metastasis. For this purpose, we evaluated datasets that were previously obtained from five different types of solid tumors including bladder (BCa) and prostate (PCa) cancers, renal cell carcinoma (RCC), cholangiocarcinoma (CC) and pancreatic cancer. To address the expected heterogeneity across different cancer types, urinary peptides were combined into a multi-marker classifier, using a support vector machine (SVM)-based algorithm^13^.

## Results

### Optimization of a urinary peptide multi-marker pattern for detection of solid tumors

**Figure 1:**
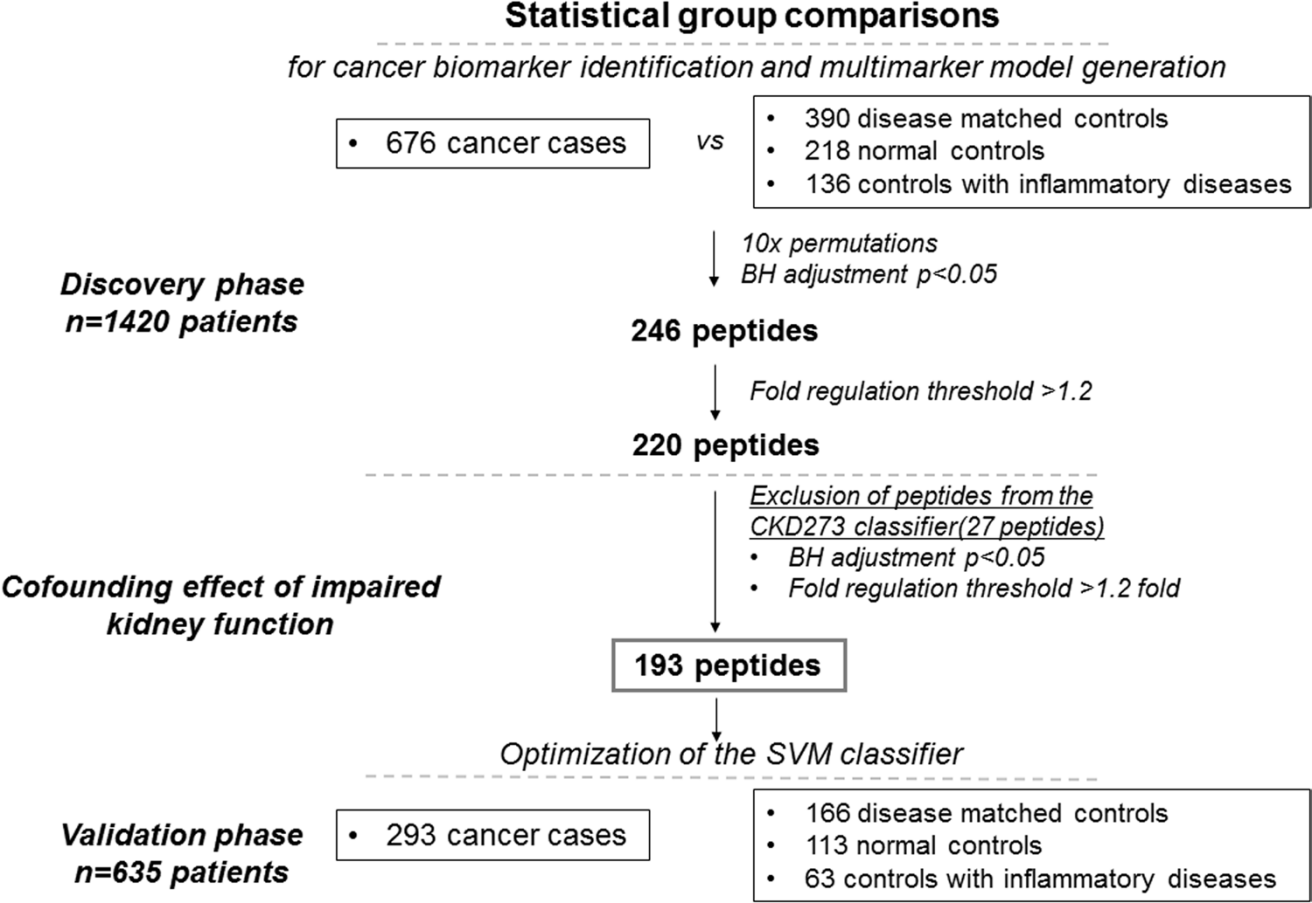
Schematic representation of the study design and workflow of the statistical analyses for the development of the urine-based peptide biomarker pattern. The statistical analysis resulted in the discovery of 193 urinary peptides discriminating between cancer cases and non-malignant controls (***p*** < 0.05). ***BH****, Benjamini-Hochberg adjustment*; ***CKD273****, chronic kidney disease 273 peptide classifier;* ***p, p****-value*.

The previously acquired CE-MS urinary peptide profiles were compared in a case- control study in a discovery set of 1,420 datasets, aiming at identifying common significant peptides in different types of cancer. The cancer group consisted of 676 cancer cases and the control group of 390 patients with tumor-related benign diseases, 218 healthy individuals and 136 patients with inflammatory diseases (Fig. 1). Statistical comparison in the discovery set between the group of cancer cases (n = 676) and non-cancer controls (n = 744), revealed 246 significantly altered peptides, after adjustment for multiple testing. By applying a fold regulation threshold of 1.2, 220 urinary peptides were retained as potential biomarkers. Of these, 100 were detected with increased and 120 with decreased abundance in the cancer group.

Since impaired kidney function may be a confounder in cancer patients, due to their advanced age, the impact of progressive loss of kidney function was assessed by investigating the potential overlap between the cancer related urinary peptides that were identified in the present study and the previously well-established chronic kidney disease (CKD) specific biomarkers^17^. By comparing the 220 cancer peptides of this study with the 273 CKD specific biomarkers that were previously reported^17^, 27 peptides were found to be overlapping and were excluded from the list of the cancer specific biomarkers (Fig. 1). The remaining 193 peptides were selected as potentially cancer specific biomarkers for the establishment of a peptide marker pattern (termed “193-general tumor marker pattern”) for detection of solid tumors using SVM (Supplementary Table S1). ROC analysis of the SVM-model on the discovery set, revealed an area under the ROC curve (AUC) of 0.85 [0.83 to 0.87 (95% CI); ***p*** < 0.0001]. In comparison, the diagnostic performance of the 193 peptides when assessed individually, ranged from 0.54–0.62 (AUC values), as presented in Supplementary Table S1. Seventeen of these peptides appear to exhibit certain cancer-type specificity as suggested following a detailed investigation of the distribution of all 193 peptides across the different cancer types (Supplementary Table S2). Since the study was not powered for this analysis, confirmation of this preliminary observation in larger datasets per cancer type is necessary to substantiate any claim of tumor specificity.

### Validation of the 193- general tumor marker pattern

**Figure 2:**
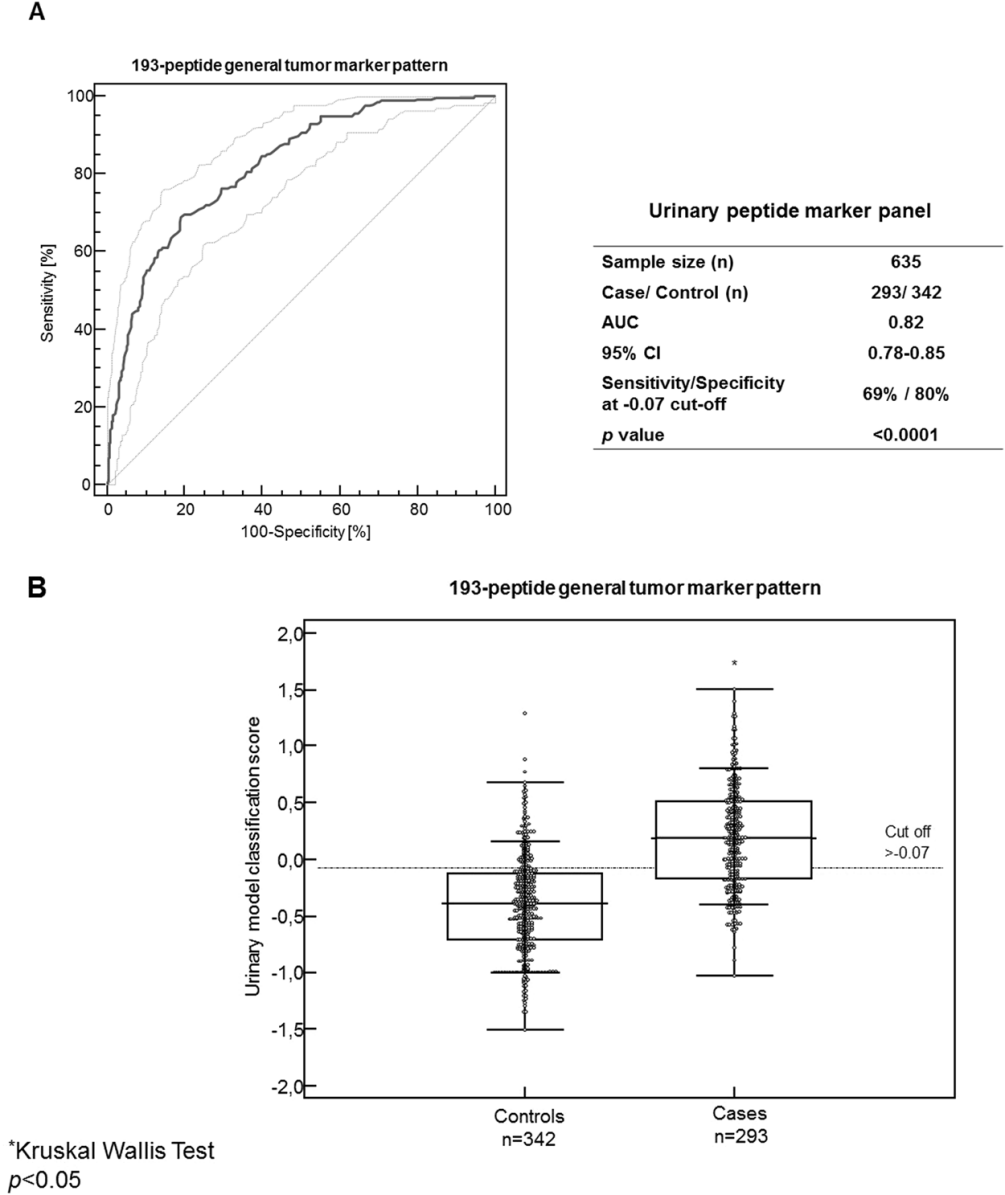
(**A**) ROC curve analysis for the urinary biomarker peptide pattern, consisting of 193 peptides, as performed in the independent validation cohort. The AUC, 95% CI, and ***p*** value are also provided for the classification of cancer patients. (**B**) The classification scores presented in Box-and-Whisker plots are displaying the level of discrimination between cancer cases and non-malignant controls and the distribution of the classification values in each population group. (**A**) Rank-test was performed using Kruskal-Wallis test. The average rank differences were significantly different (***p*** < 0.05) between the controls and cancer cases. ***AUC****, area under the curve;* ***CI****, confidence intervals,* ***ROC****, receiver operating characteristics;* ***p, p****-value*.

The 193-general tumor marker pattern was subsequently validated in an independent cohort of 635 individuals, consisting of 293 cancer cases and 342 controls including: 166 patients with benign diseases (cancer-matched controls), 113 healthy individuals and 63 patients with inflammatory diseases. ROC analysis on this validation set revealed an AUC of 0.82 [0.79 to 0.85 (95% CI); ***p*** < 0.0001]. At the pre-determined cut-off level of −0.07, the peptide marker pattern’s sensitivity and specificity were estimated at 69% and 81%, respectively (Fig. 2A). As presented in the Box-and-Whisker plot representation of Fig. 2B, the classification scores of the peptide marker pattern between the cancer case and non-cancer control groups were significantly different in post-hoc analysis after a positive Kruskal-Wallis test (***p*** < 0.0001).

To test if the 193-general tumor marker pattern could be applied to both solid and non-solid cancer types, we performed a second-phase validation in additional sets of cancer patients, available in our database of urinary peptide profiles including: a) acute myeloid leukemia (AML), n = 27 and b) patients with other solid tumor types, n = 6 [neuroendocrine cancer, n = 2 and single patients with breast cancer, small cell lung carcinoma (SCLC), bronchial cancer and hemangioma]. In the case of AML, 20 out of 27 patients were classified positively corresponding to a sensitivity of 74%. In other type of tumors (n = 6), 5 out of 6 patients scored positive resulting in a sensitivity of 83%. Additionally, we validated the 193-general tumor marker pattern in a small group of metastatic cancer cases (metastatic colon cancer, n = 1; metastatic breast cancer, n = 3, metastatic stomach cancer, n = 3). Six out of 7 of these cases scored positive corresponding to a sensitivity of 86%.

### Establishment of a diagnostic nomogram

**Figure 3:**
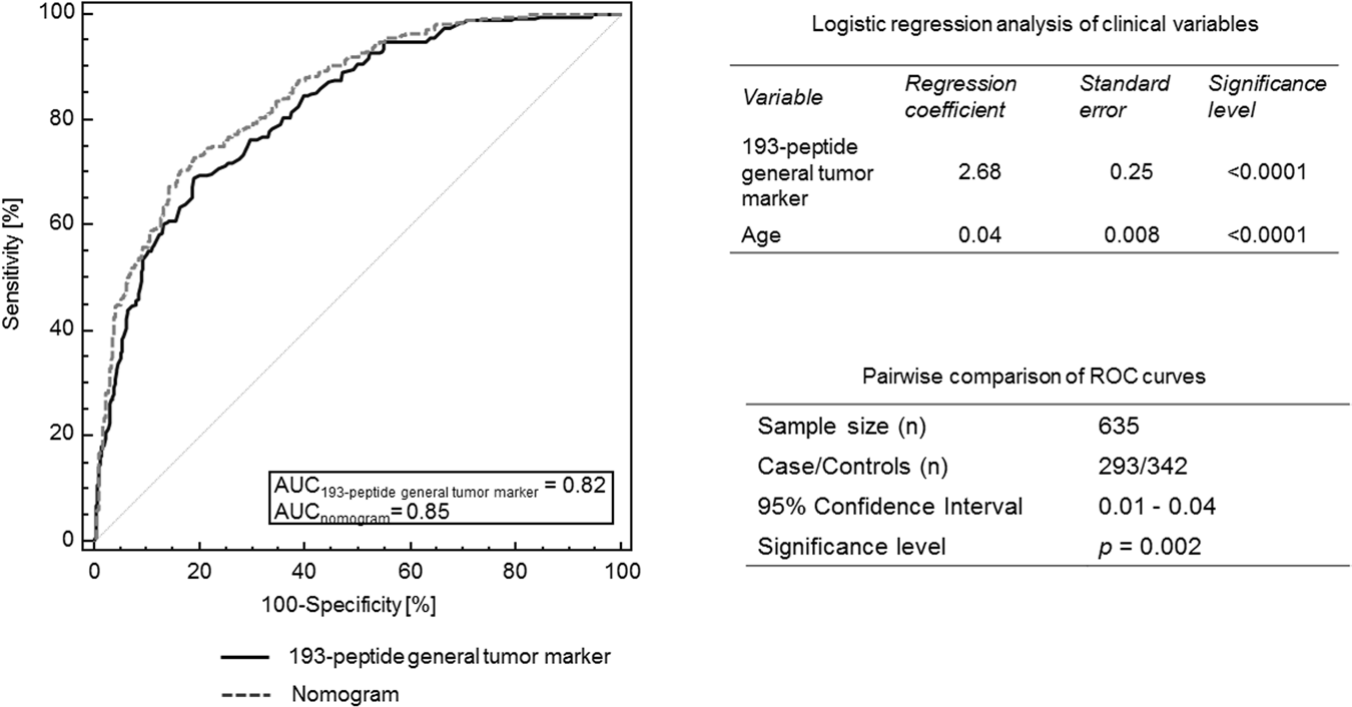
Logistic regression analysis of ROC curve and age as independent variable. ***ROC****, receiver operating characteristics;* ***AUC****, area under the ROC curve*.

**Figure 4:**
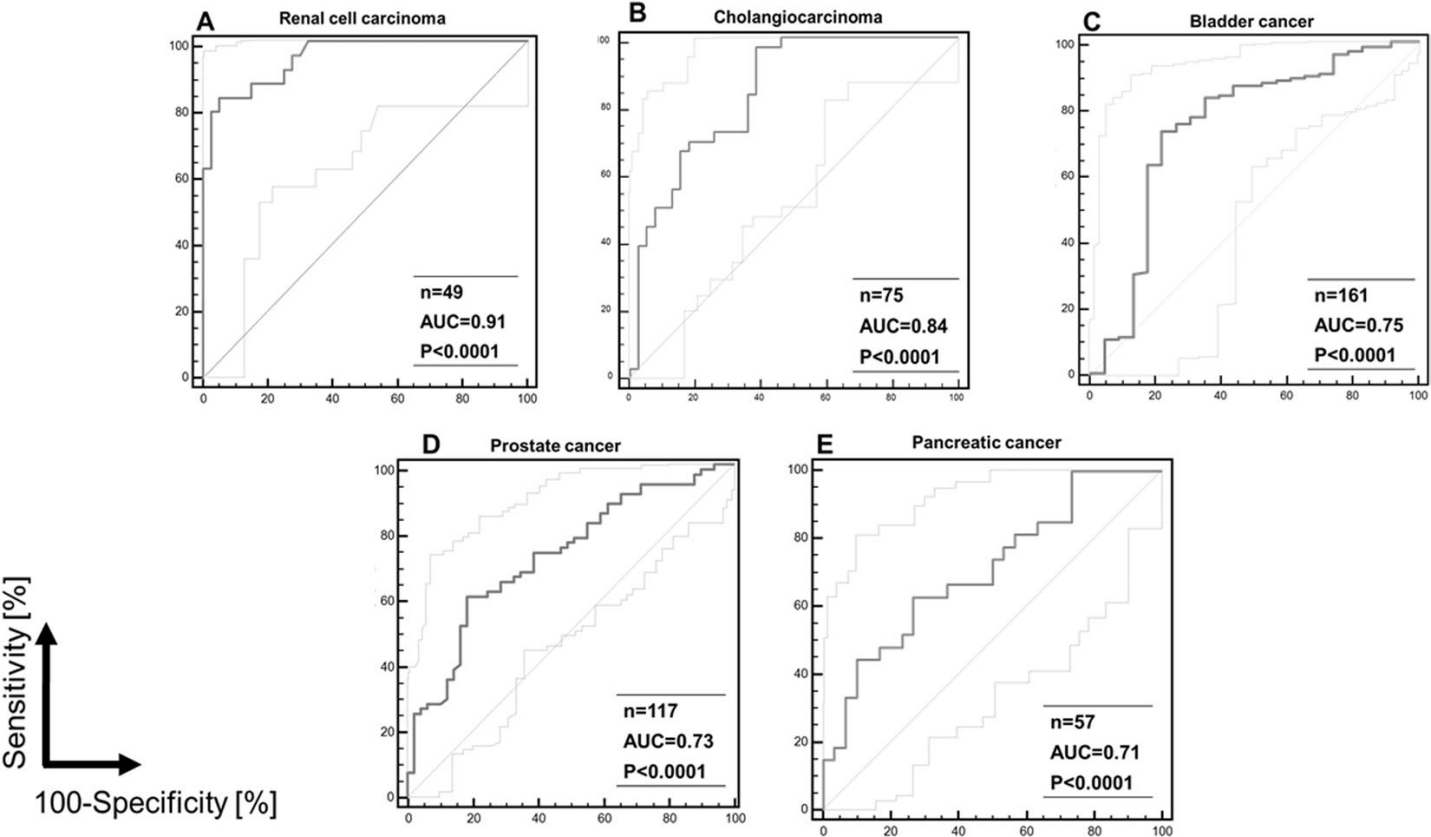
ROC curve analysis for the performance of combined patterns for: (**A**) renal cell carcinoma; (**B**) cholangiocarcinoma; (**C**) bladder cancer; (**D**) prostate cancer; (**E**) pancreatic cancer. ***ROC****, receiver operating characteristics;* ***AUC****, area under the ROC curve*.

Consecutive logistic regression analysis was performed to investigate the impact of age and gender as independent variables, on the disease occurrence. While gender did not affect significantly the occurrence, combination of the 193- general tumor marker pattern with age (***p*** < 0.0001) slightly, but significantly improved the AUC value from 0.82 to 0.85 [0.009–0.040 (95% CI); ***p*** < 0.002] (Fig. 3). As proven superior, the above nomogram was considered for further analyses. The aforementioned nomogram, generated based on the combination of the 193- general tumor marker pattern with age, was further tested separately in each group of cancer patients from the validation set (specific cancer cases and respective disease-matched controls as listed in–Supplementary Table S3) resulting in AUC values ranging from 0.71 to 0.91. Particularly, in the case of 24 RCC patients and 25 negative for RCC controls, the nomogram resulted in a high AUC value of 0.91 [0.79–0.98 (95% CI); ***p*** < 0.0001] (Fig. 4A). Similarly, for the 36 CC patients and 39 patients with benign biliary diseases an AUC value of 0.84 was obtained [0.74–0.92 (95% CI); ***p*** < 0.0001) (Fig. 4B). For the discrimination of the 138 BCa patients from the 23 respective urological controls (patients presenting with haematuria or other benign urological disorders) an AUC value of 0.75 [0.67–0.81 (95% CI); ***p*** < 0.0001] (Fig. 4C) was received, whereas 68 PCa patients and 49 patients with BPH or prostatitis were discriminated with an AUC value of 0.73 [0.64–0.80 (95% CI); ***p*** < 0.0001] (Fig. 4D). For the 27 patients with pancreatic cancer and 30 patients with chronic pancreatitis the AUC value was 0.71 [0.58–0.82 (95% CI); ***p*** < 0.0001] (Fig. 4E).

### Comparative analysis with the previously published CE-MS marker patterns

A comparison of the scoring data obtained with the use of the present nomogram and the previously published specific classifiers for each individual cancer type^8,13–16^ was subsequently performed. Only patients that were common in the independent validation sets of the previously published and the present study were compared. A direct comparison was possible for n = 161 BCa, n = 41 PCa, n = 33 CC, n =1 7 RCC and n = 31 pancreatic cancer study subjects. Based on the results of these comparative ROC analyses, the previously described biomarkers for each cancer type exhibited slightly higher accuracy in cancer detection in all cases, even though the difference did not reach statistical significance for the specific sample sets (Table 1, Supplementary Figure S1).

**Table 1:** Comparative analysis of the nomogram with the previously published specific biomarker panels. AUC, Area under the curve.

	Nomogram*	**Previously published model**	***Significance level (p-value)***	Combined patterns**	***Significance level (p-value)***	**Reference**
**Bladder cancer**	AUC = 0.75	AUC = 0.79	0.62	AUC = 0.84	0.16	Frantzi M. *et al*. 2015^14^
**Prostate cancer**	AUC = 0.66	AUC = 0.80	0.19	AUC = 0.84	0.30	Theodorescu D. *et al*. 2008^16^
**Cholangio-carcinoma**	AUC = 0.84	AUC = 0.89	0.50	AUC = 0.93	0.24	Metzger J. *et al*. 2016^8^
**Pancreatic cancer**	AUC = 0.73	AUC = 0.87	0.12	AUC = 0.87	0.35	Schonemeier B. *et al*. 2016^15^
**Renal cell carcinoma**	AUC = 0.97	AUC = 1.00	0.42	AUC = 1.00	1.00	Frantzi M. *et al*. 2014^13^

^*^Nomograms were generated based on the combination the 193-general tumor marker pattern with age as independent variable. Significance p-value was investigated between the nomograms and the previously published models.

^**^Combined patterns were developed based on the previously published specific peptide marker patterns for each type of cancer and the developed nomogram. Significance p-value was investigated between the combined patterns and the previously published models.

To investigate the added value of combining the introduced nomogram score with that of the specific cancer patterns, logistic regression analysis was performed. When examining all data combined (BCa, PCa, CC, RCC and pancreatic cancer) the combination of the tumor-specific patter with the nomogram resulted in slightly higher accuracy, (yet again not reaching statistical significance, in the available small sample set), comparing to the nomogram alone (Table 1). In detail, the AUC values as received by the combined peptide marker patterns were for BCa: AUC_combined patterns_ = 0.84, for PCa: AUC_combined patterns_ = 0.84, for CC: AUC_combined patterns_ = 0.93, for RCC: AUC_combined patterns_ = 1.00, for pancreatic cancer: AUC_combined patterns_ = 0.87 (Table 1).

### Comparative analysis of the nomogram with the common laboratory single biomarker tests

A direct comparison of the nomogram with common laboratory single biomarker tests was possible for CA19-9 and CEA levels in the case of pancreatic cancer and PSA levels in the case of PCa. When comparing the nomogram score in pancreatic cancer patients (n = 57) with the CA19-9 and CEA levels, the nomogram presented slightly higher performance than CA19-9 (AUC_nomogram_ = 0.71 vs AUC_CA19-9 level_ = 0.70, ***p*** = 0.88) and lower performance than CEA (AUC_nomogram_ = 0.71 vs AUC_CEA level_ = 0.73, ***p*** = 0.83); however in both comparisons the differences did not reach statistical significance. In the case of prostate cancer, (n = 106) the nomogram presented higher overall AUC value (0.74) in PCa detection compared to PSA, with the latter presenting an AUC of 0.63; however this difference was not significant (***p*** = 0.10). Logistic regression analysis was used for the combination of the nomogram with the commonly used single biomarker tests. In case of pancreatic cancer, combination of the nomogram with CA19-9 and CEA resulted in slightly increased AUC values (AUC_nomogram + CA19-9 level_ = 0.81, ***p*** = 0.87; AUC_nomogram + CEA level_ = 0.83, ***p*** = 0.07). In the case of prostate cancer, the combination of the nomogram with PSA presented slightly higher performance (AUC_nomogram + PSA levels_ = 0.75, ***p*** = 0.10). In both cases, the analysis did not reach statistical significance.

### Assessment of invasion and tumor-related inflammation

Based on the initial hypothesis that urinary peptides may reflect tumor invasion, the nomogram consisting of the 193-general tumor marker pattern and age, was further investigated for its potential to detect invasion. The cancer cases from the validation set were grouped into invasive (n = 54) and non-invasive cases (n = 98). Surprisingly, and as presented by Box-and-Whisker plot (Supplementary Figure S2), the 193-general tumor marker pattern showed similar discrimination abilities for detecting non-invasive and invasive cancer cases (AUC = 0.856 for non-invasive vs AUC = 0.863 for invasive cancer cases).

**Figure 5:**
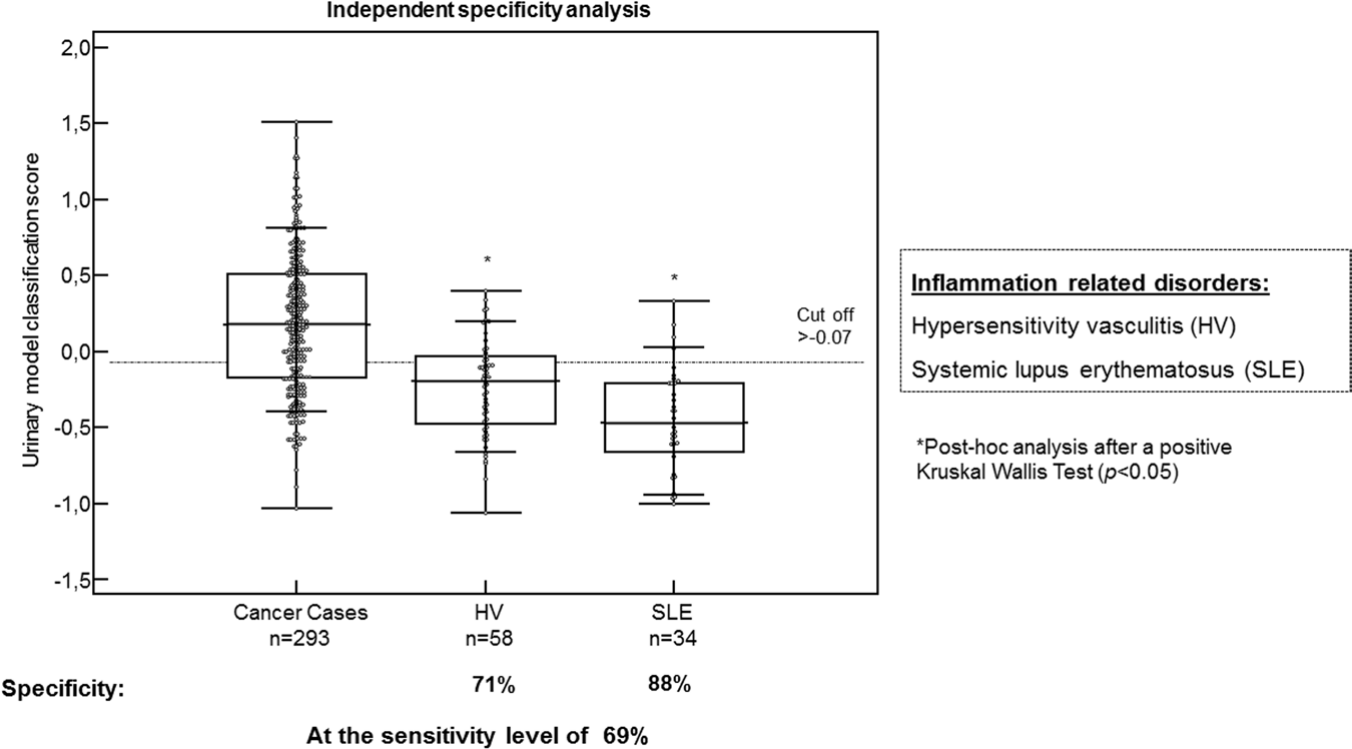
Distribution of classification values for patients with autoinflammatory diseases. The following disease aetiologies were included in the specificity analysis as reference controls: hypersensitivity vasculitis (n = 58) and systemic lupus erythematosus (n = 34). ***HV****, hypersensitivity vasculitis;* ***SLE****, systemic lupus erythematosus*.

As presented, the data indicate that the 193-general tumor marker pattern does not appear to reflect local invasion of the tumor but can detect metastasis with increased accuracy. As part of cancer related systemic features, systemic inflammation was also considered. To investigate the specificity of the peptides in terms of inflammation related to cancer, an additional set of patients with autoinflammatory diseases (hypersensitivity vasculitis (HV), n = 58, and systemic lupus erythematosus (SLE), n = 34) was used to further validate the 193-general tumor marker pattern (Table 2). The mean age of patients with autoinflammatory diseases was significantly different from the cancer patients in the validation set, therefore the nomogram could not be applicable. The specificity values were evaluated at the pre-determined cut-off of −0.07, where the sensitivity for cancer diagnosis was estimated to be 69%. At the indicated cut-off, specificity values for different autoinflammatory related diseases were 71% and 88%, for HV and SLE, respectively. As presented in Fig. 5, the scoring of the patients with inflammatory disease was not significantly different from the cancer or normal controls: the 193- general tumor marker pattern was able to significantly discriminate cancer cases from autoinflammatory diseases, as demonstrated in a post-hoc statistical analysis after a positive Kruskal-Wallis test (***p*** < 0.05) (Fig. 5).

**Table 2:** Clinical data of the patients used in the discovery and validation phases of the study. The patient cohorts were adjusted for age to be further sub-grouped in a discovery and a validation set.

Type of cancer	Group	Discovery set	Mean age (range)	Validation set	Mean age (range)	Sample size (%)
Prostate cancer	Case	N = 138	68 (41–87)	N = 68	68 (45–86)	10%
	Control	N = 130	66 (51–87)	N = 49	66 (51–83)	9%
Pancreatic cancer	Case	N = 61	65 (35–88)	N = 27	63 (33–90)	4%
	Control	N = 62	52 (27–77)	N = 30	52 (25–73)	4%
Renal cell carcinoma	Case	N = 46	62 (41–81)	N = 24	61 (33–79)	3%
	Control	N = 80	58 (23–84)	N = 25	59(23–84)	5%
Cholangio-carcinoma	Case	N = 82	56 (22–81)	N = 36	58 (30–86)	6%
	Control	N = 83	49 (20–83)	N = 39	50 (22–85)	6%
Bladder cancer	Case	N = 349	67 (28–95)	N = 138	69 (27–87)	24%
	Control	N = 23	60 (43–88)	N = 23	65 (33–93)	3%
Normal controls	Control	N = 167	60 (19–86)	N = 89	60 (22–86)	12%
	Individual with smoking history	N = 51	43(19–72)	N = 24	46(20–72)	4%
Inflammation controls	Control	N = 136	46(16–88)	N = 63	47(19–78)	10%
**Total**	**Case**	**N = 676**		**N = 293**		**47%**
	**Control**	**N = 744**		N = **342**		**53%**
Additional cancer types						
Acute myeloid leukemia				N = 27		
Other tumor types				N = 13		
Independent specificity analysis						
Inflammatory conditions		N = 92		36 (19–63)

### Peptide sequence identification

The peptide amino acid sequence was obtained for 133 out of the 193 urinary peptide biomarkers, representing a percentage of 69%. In our study, the vast majority (71%) of the sequenced CE-MS general cancer biomarkers are fragments of various collagen chains, most likely excreted into the urine by proteolytic cleavage of the extracellular matrix. Several fibrinogen α and β chain peptide fragments were also included in the marker pattern, which might be also the remark of inflammatory processes. Besides collagens, several peptide fragments originated from α- and γ-subunits of Na/K-ATPase. Single peptides from stablin-2, 14-3-3 sigma protein, keratin-1, mucin-12 and mucin-16, myeloperoxidase and apolipoprotein A-IV were also among the sequenced peptide markers. Some of the CE-MS ion peaks could not be identified by MS/MS likely due to incomplete fragmentation and/or unknown post-translational modifications that prevent alignment of the fragment spectrum to the available sequence databases^18^.

To assess the “tissue invasion” hypothesis, we further selected only the collagen and fibrinogen peptide fragments and evaluated their diagnostic capabilities. ROC analysis in the independent validation set revealed an AUC value of 0.77 [0.73 to 0.80 (95% CI); ***p*** < 0.0001]. The AUC values for each particular cancer type were: for bladder cancer: AUC = 0.68; for prostate cancer: AUC = 0.66, for cholangiocarcinoma AUC = 0.82, for renal cell carcinoma AUC = 0.85 and for pancreatic cancer: AUC = 0.54. The SVM based pattern that was developed based on collagen and fibrin peptide fragments alone, exhibited significantly lower performance than the 193- general tumour marker pattern (AUC = 0.82, ***p*** < 0.0001).

## Discussion

In this study, we aimed at the development of a general tumor marker panel based on pre-existing CE-MS urinary profiling data, being available from previously published studies^8,13–16^. The study was based on the hypothesis that tumor growth is associated with general molecular changes, representing systemic inflammation triggered by tissue invasion and metastasis. Therefore, general tumor-associated changes may be valuable in monitoring patients for response to therapy, and/or tumor relapse. As such, the clinical context for application of such panel is not envisioned in cancer initial detection/screening, (where identification of the specific tumor type is obviously a requirement), but rather as an adjunct during disease monitoring, most likely in combination also with tumor-specific markers, as available (such as CA125, PSA, as described below). As a proof-of-concept study, we evaluated previously published CE-MS peptide profiles of more than 2,000 cancer patients and individuals with non-malignant diseases and developed a marker panel of 193 peptides presenting statistical significant changes between the (multi)cancer group and controls. Based on previous results^8,13,14^, the combination of several individual peptide markers in an SVM-based multi-marker pattern allows counteracting variability in single biomarkers, hence for higher accuracy, particularly regarding for cancer types characterised by very high intra-tumor heterogeneity.

The sensitivity and specificity levels of the 193 peptide panel revealed satisfactory discriminatory ability in solid, but also non-solid tumors, with sensitivity values to be higher in detecting metastasis at least based on a small set of available metastatic samples. Compared to the previously published cancer type-specific patterns (BCa, PCa, CC, RCC and pancreatic cancer patterns), and with the acknowledgement that the power of this comparison is compromised by the available small sample sizes, the 193- general tumor marker pattern performed with slightly lower AUC values for each individual cancer type. However, combination of the two tests increased accuracy in cancer detection, further suggesting that such a panel may be useful as an adjunct during cancer monitoring, in combination to more cancer type specific tests.

Along these lines, a direct comparison with commonly applied diagnostic tests when available, (CA19-9 and CEA levels for pancreatic cancer and PSA levels for prostate cancer), improved the discrimination of cancer patients, even though the difference was again not significant. These results collectively underscore the need for validation studies further testing the accuracy of the 193-peptide marker pattern in comparison and/or in combination to cancer type specific markers, as an effort to combine knowledge on cancer “systemic” reactions with cancer-type specific events towards improving disease monitoring.

From the 193 tumor-specific peptides, sequence information could be retrieved for 133 peptides. Most of the tumor-specific peptides are fragments of collagen chains, possibly reflecting molecular changes in the extracellular matrix (ECM) organization and the altered activity of ECM-degrading proteases during tumor progression^19^. In the present study, increased urinary levels of fibrinogen-derived peptide fragments were identified in cancer patients. Importantly, one specific peptide, produced by the cleavage of fibrinogen beta chain (FGB) was frequently identified as cancer biomarker in three previously published CE-MS studies, investigating bladder cancer, renal cell carcinoma and pancreatic cancer respectively^13–15^. Fibrinogen is important in tumor stroma-formation, as one of the major components linking the tumor cells to the ECM and once converted to insoluble fibrin by activated thrombin, is responsible for the promotion of platelet coagulation. In addition, fibrinogen is considered as an acute phase reactant protein and is elevated during inflammation processes. In leukemias, high fibrinogen levels are associated with systemic inflammatory response, that is frequently present in AML, and is associated with poor outcome^20^.

Additionally, in the presented study, decreased urinary levels of a myeloperoxidase (MPO)- derived peptide were detected in urine of cancer patients, in comparison to the non-cancer controls. MPO is a lysosomal enzyme produced in high amounts by neutrophilic granulocytes in respect to inflammatory responses^21^. Multiple studies link the G463A polymorphism in the MPO promoter region, which decreases the transcription and activity of MPO, with a lower cancer risk for a variety of human cancers e.g. pancreatic cancer^22^, bladder cancer^23^ and gastric cancer^24^. In addition, peptides from mucin 16 (MUC16) were detected with decreased levels in the urine of cancer patients. Mucins are heavily O-glycosylated proteins released from the cell surface as a result of proteolytic cleavage of their transmembrane segment^25^. MUC16 has been reported to be an important factor in tumor diagnosis since it contains the CA125 cancer antigen which is strongly deregulated in pancreatic cancer. Moreover, MUC16 contributes to the ovarian cancer growth and its metastatic activity^25^. The cancer cells expressing low levels of MUC16 on their surface are the preferential target for the Natural killer (NK) cells, contributing to immunoediting^25^.

To further investigate the hypothesis that specific urinary peptides are related to tissue invasion, we selected only the peptides that are expected to be involved in ECM remodelling and tissue rearrangement during cancer invasion, such as collagens and fibrinogen fragments. However, by combining them in specific peptide pattern, the performance was significantly lower (AUC = 0.77, ***p* < **0.0001), compared to the 193- general tumor marker pattern. When investigating the discriminatory potential in the differentiation of non-invasive and invasive cancer cases, the results also supported that there is no significant difference in the diagnostic potential between non-invasive and invasive tumors. This observation was further supported by the fact that the general tumor marker pattern detected AML patients with good sensitivity (74%). Considering the above results and the fact that AML is a non-solid tumor, we concluded that the molecular changes reflected by the urinary peptide profiles are more likely related to general systemic effects during cancer progression rather than local tumor invasion. This is also suggested by the fact that the vast majority of peptides included in the panel do not appear to be cancer-type specific.

As revealed by the specificity analysis in patients that present inflammation but have no cancer, the established nomogram can well differentiate between the patients presenting autoinflammatory diseases and those suffering from cancer. The good discriminatory capability of the established general tumor pattern suggests that the urinary peptides included in marker pattern may be specific to cancer-related inflammatory processes, distinctive from inflammation properties that occur during autoimmune disease. This observation is in line with the literature, suggesting that tumor-associated inflammation seems to be promoted through a distinct intrinsic pathway^26^. More specifically, a unique orchestration of chemokines, secreted factors and immune cells is present at all stages of tumorigenesis, contributing to the tumor initiation and progression. Even more prominently, particular molecules are reported to be involved specifically during metastasis, where inflammation seems to promote and assist the colonization of the tumor cells in the metastatic niche^4,26^.

There are some limitations to be noted in the current work which warrant further consideration. Although performed in a large cohort, the presented proof-of-concept study was retrospective.

As such, the clinical and pathological data in some cases were missing, among others the level of the standard biochemical biomarkers (PSA, CA19-9 and CEA) and stage. Moreover, information about the patient’s comorbidities and additional information on the administered treatment was not possible to be retrieved. Therefore, the diagnostic ability of the 193-peptide marker pattern needs to be further confirmed prospectively in a proper clinical setting: during cancer monitoring and in parallel to well established cancer-type specific markers.

Based on the results, the 193 tumor-specific peptides combined into a marker pattern presented good accuracy, in comparison to the single peptide biomarkers alone. Addition of age as independent covariate slightly, yet significantly, improved the performance of the multi-marker pattern. However, considering the advanced age of cancer patients (mean of age 67) the developed nomogram may not be applicable to significantly younger individuals (e.g. AML patients with mean value for age of 50).

In addition, some of the peptides (31%) included in the general tumor marker could not be sequenced, mainly due to the incomplete fragmentation or presence of unknown post-translational modifications. Nonetheless, a large number of peptide sequences were retrieved and found to reflect processes involved mainly in cancer related systemic events, further supporting the biological validity of the findings.

Collectively, through presented study we aimed to identify commonly-shared peptide markers between five cancer types that are more likely related to general systemic effects during cancer progression. The envisioned application is a general tumor marker that can detect relapse of tumor is the primary site and/or distant metastasis even after the primary tumor has been removed. Such a marker pattern would have potential not only in monitoring cancer recurrence, but also response to the treatment, e.g. to chemotherapy. However, to support these aims, a prospective study in an appropriate number of patients is needed, where the biomarker pattern is tested in comparison to the current state-of-the-art.

## Methods

### Patient selection

In this multi-centre study, a case-control comparison was performed based on available CE-MS datasets from previous studies^8,13–16,27^. The study was performed in accordance with the guidelines for biomarker identification and reporting in clinical proteomics^28^ and the REMARK Reporting Recommendations^29^. The study was conducted in accordance with the Declaration of Helsinki and ethical approval was obtained by the Ethics Committee from Hannover Medical School in Germany (ID: 3409-2016). Proper informed consent procedures were followed under Institutional Review Board–approved protocols of the participating centers. All experimental protocols for sample collection and processing were performed according to relevant guidelines and the standard protocols for urine collection, as defined by the European Kidney and Urine Proteomics (EuroKUP) and Human Kidney and Urine Proteome Project (HKUPP) networks and described in the respective publications. Available urinary CE-MS peptide profiling datasets from patients presenting five types of solid tumors were selected from an internal database^27^. The distribution of the sample size per cancer type is presented in Supplementary Table S3. The clinical and demographical data per patient are reported in Supplementary Table S4. A total of 2,055 urinary profiles from case and control groups (Table 2) were statistically evaluated for differences in their peptide composition. A total number of 487 urinary peptide profiling datasets from patients with bladder cancer and 58 from disease-matched controls with non-malignant urological pathologies (e.g. haematuria, benign urologic diseases) were included in the analysis, as described in^14^. Peptide datasets from urine samples collected from prostate cancer patients (n = 206) and patients with benign prostate diseases (e.g. benign prostatic hyperplasia or prostatitis; n = 179), described in^16^ were also included. Previously published CE-MS profiling datasets^13^ from 70 renal cell carcinoma patients and 105 individuals negative for malignancy were also included. Urinary CE-MS datasets from 240 patients with cholestasis were considered^8^. Among the 240 patients, 118 patients were diagnosed with cholangiocarcinoma and 122 patients with benign diseases (e.g. primary sclerosing cholangitis (PSC) or benign biliary disorders (BBD)), were used as disease matched controls^8^. Moreover, urinary datasets from patients with pancreatic cancer (n = 88) or chronic pancreatitis (n = 92)^15^ were included in the analysis. Additional urinary CE-MS datasets included in the control group were from non-diseased subjects (n = 256), individuals with smoking history (n = 75) and patients with inflammatory diseases (n = 199) (e.g. arteriosclerosis, IgA nephropathy and systemic lupus erythematosus).

The general tumor marker pattern was tested in additional groups of cancers including: acute myeloid leukemia (AML), n = 27^30^ and patients with other solid tumors: neuroendocrine cancer, n = 2; metastatic colon cancer, n = 1; metastatic stomach cancer, n = 3; metastatic breast cancer, n = 3; breast cancer, n = 1; small cell lung carcinoma, n = 1; bronchial cancer, n = 1; and hemangioma, n = 1^27^. Additionally, an independent analysis was performed to assess the specificity of the general tumor marker pattern in a control group of patients with inflammation-related diseases, like HV (n = 58) and SLE (n = 34), as previously published (Table 2)^31^.

### Evaluation of the CE-MS acquired datasets

In the context of this study, previously published CE-MS data sets were investigated^8,13–16^. In all of these, the same analytical workflow had been followed following standard operating protocols (SOPs) for routine diagnostics^8,32^. The established SOPs and ISO standards, allow to obtain highly comparable data, which are further integrated in the database, in order to perform comparisons targeting a wide spectrum of clinical applications.

The sample collection was performed in according to the established SOPs described in the respective publications. In brief, 10 ml of sample were collected, splitted into aliquots and immediately frozen at −20 °C. No protease inhibitors were added prior to the analysis. The sample preparation was conducted based on established SOPs, as previously described^8,32^. Briefly, 700 μl urine aliquots were diluted 1:2 in an alkaline buffer containing 2 M urea, 10 mM NH_4_OH and 0.02% SDS (pH 10.5). Subsequently, the samples were filtered by Centrisart ultracentrifugation filters (Sartorius, Göttingen, Germany) to remove proteins below 20 kDa, followed by desalting over PD-10 columns (GE Healthcare, Munich, Germany). The peptide extracts were lyophilized and resuspended in high-performance-liquid-chromatography-grade water. CE-MS analysis and data processing was performed according to ISO13485 standards yielding quality controlled urinary data sets^32^. Mass spectral ion peaks representing identical molecules at different charge states were de-convoluted into single masses using MosaiquesVisu software^13,32^. The peak list characterizes each peptide by its molecular mass [kDa], normalized migration time [min] and normalized signal intensity [AU]^13^. Normalization of the CE-MS data was based on 29 collagen fragments that are not affected by disease and serve as internal standards^27^. After normalisation, all detected peptides were deposited, matched, and annotated in a Microsoft SQL database and used as input in the presented study. As proteomics datasets frequently contains missing values, due to biological and/ or technical factors, in this study missing values (“0 values”) were replaced by the minimum intensity value per dataset. Subsequent transformation of the data (log-transformation) was performed, as previously described^33^.

### Statistical analysis

A case-control statistical comparison was conducted to detect potentially significant cancer specific-peptide biomarkers (Fig. 1). The datasets were grouped in a cancer (n = 969) and a control set (n = 1,086) and divided into a discovery and validation set, as described below and presented in Fig. 1.

#### Discovery phase

The peptide profiles were compared for differences in the peptide urinary excretion levels between cancer cases (n = 676) and non-cancer controls (n = 744) by applying the Wilcoxon rank sum test^33^. To increase the validity of the statistical approach, permutation analysis was performed by excluding randomly 30% of the samples, further repeated ten times. Statistical correction of the estimated ***p*** values for multivariate testing was performed based on the Benjamini-Hochberg method^34^. The common significant identified peptides (***p*** < 0.05 after Benjamini-Hochberg adjustment), obtained in all ten permutation analyses and exhibiting at least a 1.2-fold change between cases and controls were considered for further analysis. The presence of cancer-type specific peptides among the 193 peptides was investigated by performing a similar analysis in each cancer type set separately.

#### SVM Optimization

The urinary marker pattern was optimized in the training set, using the SVM-based MosaCluster software (version 1.7.0), developed for the classification of samples in a high dimensional parameter space according to SVM-based algorithms. The classifier was optimised based on the shortlisted cancer specific biomarkers with each biomarker representing one dimension in the n-dimensional parameter space^13^. The SVM kernel parameters were defined and applied during classifier validation as following: C = 4.096, gamma = 0.0064 and epsilon = 0.001.

#### Validation phase

Validation was performed in an independent set of 635 samples, grouped into 293 cancer cases and 342 non-malignant controls. The sensitivity and specificity estimates for the SVM-based peptide marker pattern were calculated based on the number of correctly classified samples. The receiver operating characteristic (ROC) plots and the confidence intervals (95% CI) were based on exact binomial calculations performed in MedCalc 12.7.5.0 (Mariakerke, Belgium). The optimal cut-off level was pre-determined according to the criterion value calculated based on the Youden index J, giving an equal weight to sensitivity and specificity (MedCalc). Statistical comparisons of the classification scores between the cancer cases and control groups of the validation cohort were performed using Kruskal-Wallis rank sum test (MedCalc).

#### Logistic regression analysis

Logistic regression analysis was used to evaluate clinical impact of independent clinical variables (e.g. age, gender, PSA, CA 19-9 and CEA levels) on the diagnostic outcome of the multi-marker pattern. Diagnostic nomograms were developed, by combining the 193-general tumor marker pattern with age, CEA, CA19-9 and PSA levels (MedCalc). The same method was applied to assess the added value of the 193-general tumor marker pattern over the previously published CE-MS-based classifiers.

### Peptide sequencing and matching

The amino acid composition was acquired by MS/MS analysis using either a PACE CE or a Dionex Ultimate 3000 RSLS nanoflow system (Dionex, Camberly UK) coupled to an Orbitrap Velos instrument (Thermo Scientific), as previously described^18^. Protein matching and data analysis was based on Proteome Discoverer 1.2 (activation type: HCD; precursor mass tolerance: 5 ppm; fragment mass tolerance: 0.05 Da). The data were searched against the UniProt human database^35^ without enzyme specificity. Matching of the amino acid sequences with the CE-MS acquired ion peaks was based on mass correlation between CE-MS and liquid chromatography-tandem mass spectrometry analysis (LC-MS/MS). Further validation of the obtained peptide identifications is based on the assessment of the peptide charge at the working pH of 2.2 and the CE-migration time results^32^.

### Data avability

The datasets generated during and/or analyzed during the current study are available from the corresponding author on reasonable request.

## Electronic supplementary material

Supplementary Materials
