# Author Correction: Urinary CE-MS peptide marker pattern for detection of solid tumors

**DOI:** 10.1038/s41598-019-49304-9

**Published:** 2019-09-03

**Authors:** Iwona Belczacka, Agnieszka Latosinska, Justyna Siwy, Jochen Metzger, Axel S. Merseburger, Harald Mischak, Antonia Vlahou, Maria Frantzi, Vera Jankowski

**Affiliations:** 1 Mosaiques Diagnostics GmbH, Hannover, Germany; 2 University Hospital RWTH Aachen, Institute for Molecular Cardiovascular Research (IMCAR), Aachen, Germany; 3 Department of Urology, University of Lübeck, Lübeck, Germany; 4 University of Glasgow, Institute of Cardiovascular and Medical Sciences, Glasgow, United Kingdom; 5 Biotechnology Division, Biomedical Research Foundation, Academy of Athens (BRFAA), Athens, Greece

Correction to: *Scientific Reports* 10.1038/s41598-018-23585-y, published online 27 March 2018

The Author Contributions section in this Article is incorrect.

“I.B., M.F. and A.L. wrote the main manuscript text. I.B., M.F., A.L., J.S. and J.M. analyzed the peptidomics data and performed the statistical analysis. V.J., A.V., J.S., J.M., A.S.M. and H.M. contributed to the writing of the manuscript. I.B. prepared all figures, tables and supplementary materials. All authors reviewed the manuscript.”

should read:

“I.B. wrote the manuscript, performed data collection and evaluation, performed experiments, interpretation of peptidomics data and statistical evaluation. I.B., A.L., J.S., H.M., M.F. and V.J. participated in study supervision. I.B., H.M., A.V., M.F. and V.J. served as scientific advisors in consulting the study design. I.B., J.S. and J.M. contributed to examination of study participants. I.B., A.L., J.S., J.M. and M.F. contributed to data evaluation, interpretation of peptidomics data and statistical evaluation. I.B., J.M., A.S.M., H.M. and M.F. participated in delivery of the materials. I.B., A.L., J.S. and M.F. participated in performing experiments (sequencing of peptides). I.B., A.L. and M.F. contributed to performing experiments (specificity analysis and validation in additional set of cancer patients). I.B. prepared all figures, tables and supplementary materials. All authors corrected the manuscript and gave the final approval of the version to be published.”

