# Supplementary material for: Urinary CE-MS peptide marker pattern for detection of solid tumors
Source: Sci Rep. 2018 Mar 27;8:5227. doi: 10.1038/s41598-018-23585-y (PMC5869723; doi:10.1038/s41598-018-23585-y)
Supplement: Supplementary file 1 — Supplementary Materials [file 41598_2018_23585_MOESM1_ESM.pdf]

Urinary CE-MS peptide marker pattern for detection of solid tumors

Iwona Belczacka[1,2], Agnieszka Latosinska[1], Justyna Siwy[1], Jochen Metzger[1], Axel S. Merseburger[3], Harald Mischak[1,4], Antonia Vlahou[5], Maria Frantzi[1], Vera Jankowski[2*]

[1]*Mosaiques Diagnostics GmbH, Hannover, Germany;*

[2]*University Hospital RWTH Aachen, Institute for Molecular Cardiovascular Research (IMCAR), Aachen, Germany;*

[3]*Department of Urology, University of Lübeck, Lübeck, Germany;*

[4]*University of Glasgow, Institute of Cardiovascular and Medical Sciences, Glasgow, United Kingdom;*

[5]*Biotechnology Division, Biomedical Research Foundation, Academy of Athens (BRFAA), Athens, Greece;*

Supplementary Files

Supplementary Figure S1. Comparison of the classification scores with the previously published peptide marker patterns for the detection of **A.** bladder cancer, **B.** prostate cancer, **C.** cholangiocarcinoma, **D.** renal cell carcinoma, **E.** pancreatic cancer. ***AUC****, Area under the curve.*

Supplementary Figure S2. Representation of the nomogram classification scores between non-invasive and invasive cancer cases. The optimal cut-off (>0.54) was estimated based on the nomogram scores according to the Youden index.

Supplementary Table S1. List of the 193 peptides that were included in the peptide biomarker panels for the detection of solid tumors. ***Amp****,* Amplitude; ***AUC****, Area under the curve;* ***BH****, Benjamini/ Hochberg;* ***CE-MS****, Capillary Electrophoresis coupled to Mass Spectrometry;* ***Da****, Dalton;* ***min****, minutes;* ***FDR adj.****, False Discovery Rate adjustment.*

Supplementary Table S2. Investigation of the distribution of the 193 peptide markers in individual cancers. Statistical analyses were performed in the discovery set using the Wilcoxon rank sum test for each cancer group separately, followed by adjustment using Benjamini-Hochberg method. Each analysis was repeated ten times by excluding randomly 30% of the samples in both groups and applying the frequency threshold of 70% (at least in one group). The results of the analysis are presented in the table. Adjusted *p*-values of the Wilcoxon rank sum test are reported. Non-significant peptides are reported as n.s. N/A denotes peptides that were not commonly detected in all 10 permutations with at least 70% frequency. Peptides that were observed as statistically significant in the discovery set of one cancer type (adj. *p*-value highlighted in red) were considered for further analysis in the validation set (unadjusted *p*-values of Wilcoxon rank sum test are reported). Only those peptides that were observed as statistically significant in both discovery and validation sets are considered as cancer-type specific peptides (17 peptides highlighted in grey color). ***CE-MS****, Capillary Electrophoresis coupled to Mass Spectrometry;* ***Da****, Dalton;* ***min****, minutes;* ***N/A****, peptides that were not commonly detected in all 10 permutations with at least 70% frequency;* ***P****, p-value;* ***n.s****, non-significant.*

Supplementary Table S3. Distribution of the clinical data of patients used in the study.

Supplementary Table S4. Clinical and demographical data for the full list of 2,055 urinary profiling datasets, from patients presenting with one of five types of solid tumors, separated in 1,420 urine samples that were included in the discovery phase and 635 urine samples that were included in the validation phase.

Supplementary Figure S1

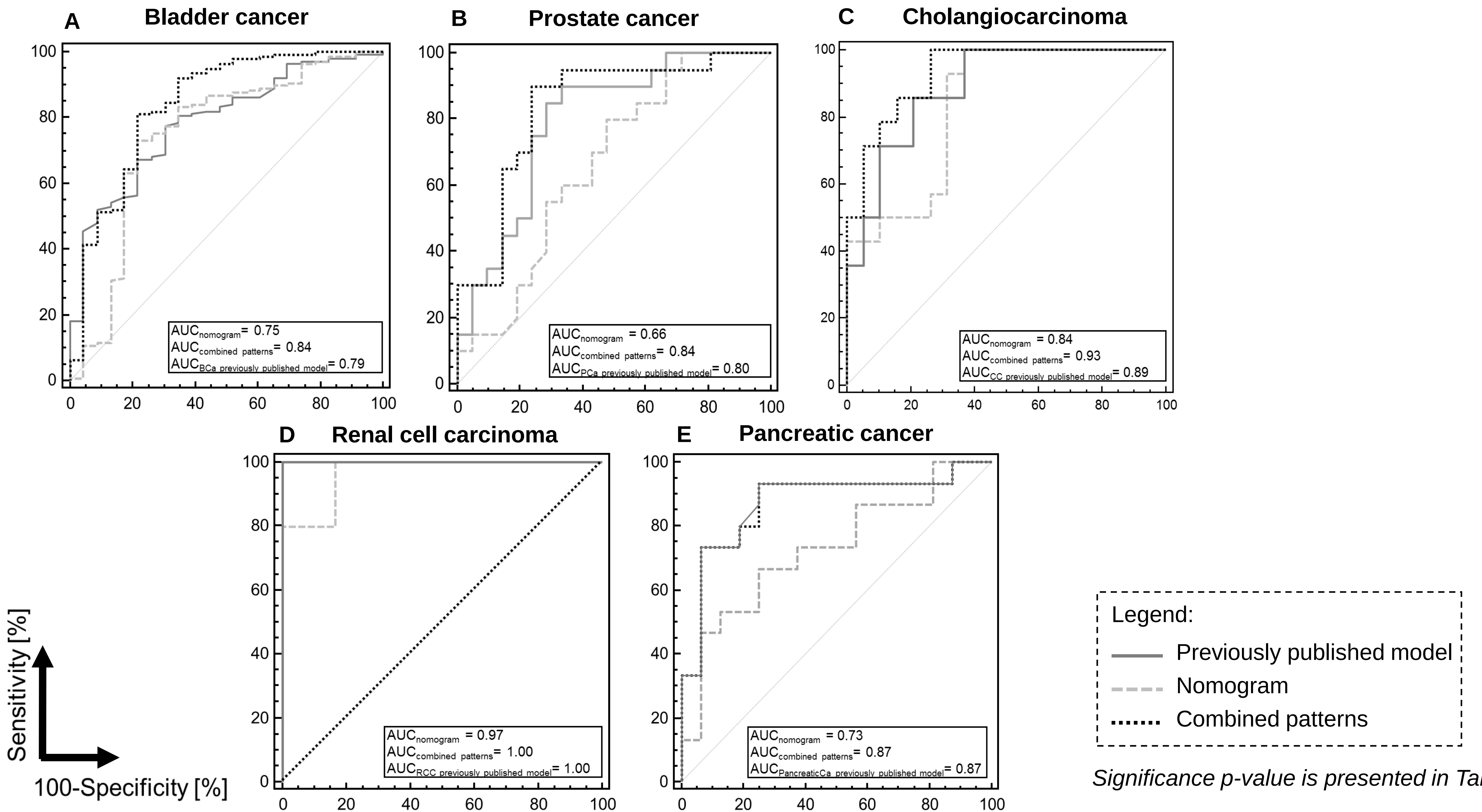

Significance p-value is presented in Table 1.

Supplementary Figure S2

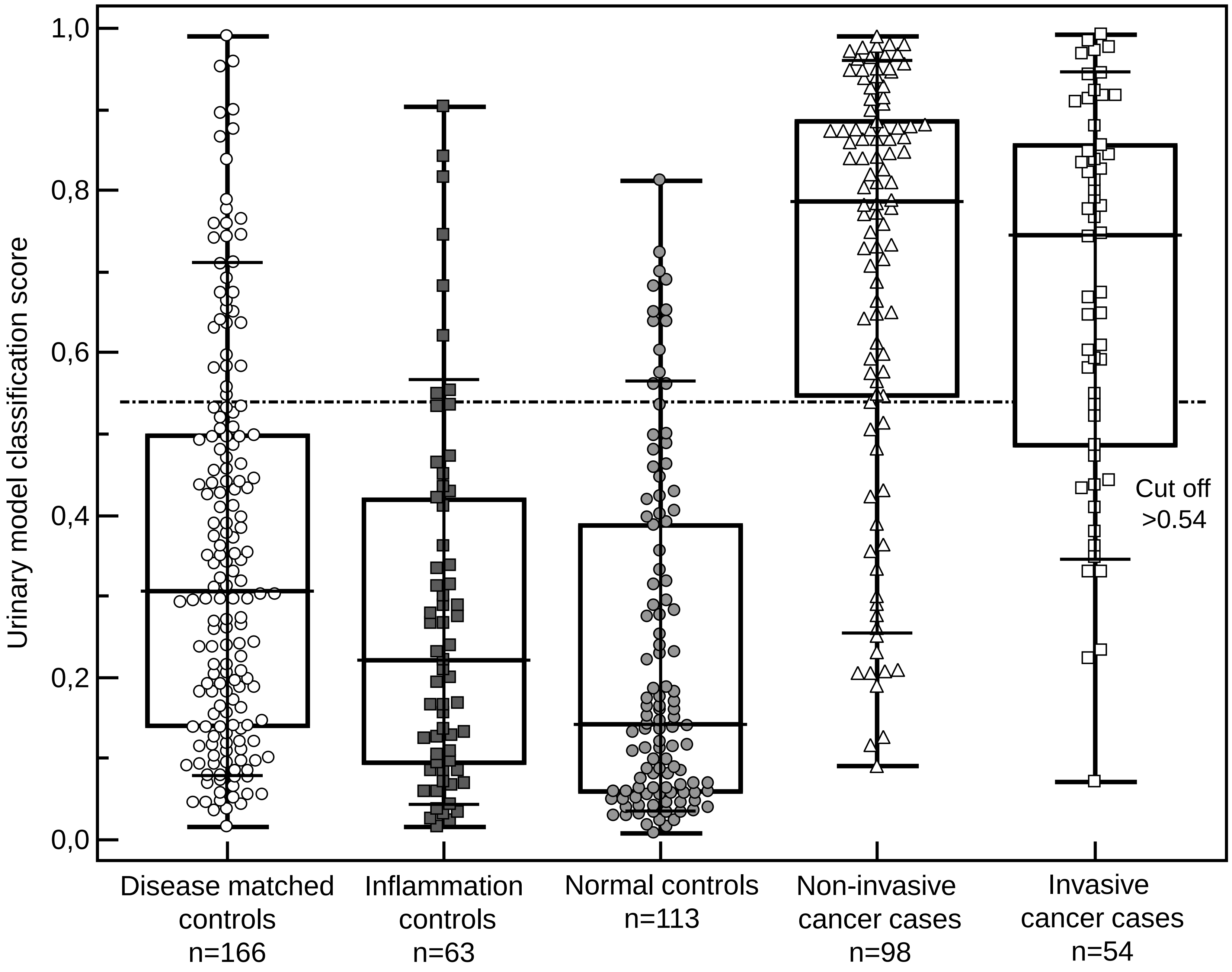

Supplementary Table S1. List of the 193 peptides that were included in the peptide biomarker pattern for detecting of solid tumors

CE-MS characteristics			Biomarker panel					Statistical analysis		
			Distribution in cancer cases (n=676)		Distribution in cancer controls (n=744)					
Mass [Da]	CE Migration time (min)	Protein name	Mean Amp (SD)	Frequency	Mean Amp (SD)	Frequency	Fold change (Mean Amp cases/Mean Amp controls)	p value I	p value II (FDR adj. P)	AUC
884,32	24,72	Cadherin-13	327(879)	69	465(1227)	79	0,70	6,62E-04	3,57E-03	0,56
928,37	35,53	Collagen alpha-5(VI) chain	24(151)	34	40(92)	22	0,61	1,11E-03	5,25E-03	0,55
949,22	34,54		314(122)	48	649(199)	57	0,48	2,85E-04	1,73E-03	0,56
956,45	20,35	Mucin-12	76(605)	76	124(711)	81	0,61	2,49E-04	1,55E-03	0,56
980,29	35,64		198(159)	49	106(121)	35	1,86	7,20E-04	3,85E-03	0,56
992,42	20,37	PHD finger protein 10	12(513)	64	29(743)	74	0,42	2,11E-08	1,09E-06	0,58
1007,31	35,68		111(5529)	66	60(3331)	59	1,83	4,15E-04	2,39E-03	0,56
1013,37	25,06	Inositol 1,4,5-trisphosphate receptor type 2	2572(2468)	42	3345(3203)	63	0,77	1,62E-03	7,08E-03	0,56
1060,38	38,03		46(1132)	86	73(1310)	89	0,63	7,36E-03	2,36E-02	0,54
1069,48	25,33	Collagen alpha-1(III) chain	225(71)	16	342(130)	33	0,66	1,96E-03	8,22E-03	0,55
1075,49	20,68	Myeloperoxidase	54(207)	32	120(229)	43	0,45	1,66E-08	9,89E-07	0,59
1084,43	25,18	Collagen alpha-1(II) chain	1718(930)	29	2327(812)	41	0,74	1,52E-04	1,08E-03	0,57
1099,49	28,06	Sodium/potassium-transporting ATPase subunit gamma	467(757)	85	665(956)	85	0,70	6,06E-07	1,34E-05	0,59
1113,47	27,24	14-3-3 protein sigma	215(578)	19	351(239)	33	0,61	1,20E-09	9,06E-08	0,61
1114,48	24,04	Collagen alpha-1(III) chain	95(530)	48	151(703)	56	0,63	2,34E-04	1,50E-03	0,56
1115,51	21,6	Collagen alpha-1(III) chain	162(914)	33	130(291)	43	1,25	1,61E-03	7,05E-03	0,55
1128,4	33,1		965(205)	32	1545(244)	39	0,62	8,22E-07	1,67E-05	0,59
1129,46	27,75	Collagen alpha-1(VII) chain	180(288)	38	254(165)	24	0,71	2,31E-04	1,49E-03	0,57
1161,5	37,6	Collagen alpha-1(XV) chain	66(738)	31	35(755)	41	1,86	6,06E-05	5,54E-04	0,56
1178,38	20,44	GC-rich sequence DNA-binding factor 1	73(208)	43	112(322)	50	0,65	1,16E-03	5,39E-03	0,55

CE-MS characteristics			Biomarker panel					Statistical analysis		
			Distribution in cancer cases (n=676)		Distribution in cancer controls (n=744)		Fold change (Mean Amp cases/Mean Amp controls)			
Mass [Da]	CE Migration time (min)	Protein name	Mean Amp (SD)	Frequency	Mean Amp (SD)	Frequency		p value I	p value II (FDR adj. P)	AUC
1181,48	36,8	Collagen alpha-1(I) chain	66(602)	66	106(722)	76	0,62	1,89E-06	3,56E-05	0,57
1182,55	28,2	Collagen alpha-3(IV) chain	34(1189)	60	52(4461)	67	0,65	1,08E-04	8,66E-04	0,56
1208,38	45,14		96(138)	31	173(190)	39	0,55	1,26E-03	5,80E-03	0,55
1215,44	26,94		290(378)	51	465(393)	38	0,62	2,62E-04	1,62E-03	0,56
1216,54	24,59		1133(274)	34	1495(357)	46	0,76	2,05E-05	2,23E-04	0,58
1217,48	27,78	E3 ubiquitin-protein ligase UBR2	128(170)	58	248(103)	53	0,52	2,17E-09	1,45E-07	0,59
1224,36	45,35		133(1650)	95	199(1738)	98	0,67	1,21E-03	5,61E-03	0,55
1226,53	21,11		440(1163)	36	576(485)	24	0,76	8,60E-04	4,35E-03	0,56
1232,52	38,06	Collagen alpha-1(II) chain	98(1239)	52	47(1320)	63	2,10	2,64E-05	2,79E-04	0,56
1239,43	33,58	Myb-binding protein 1A	243(868)	50	198(594)	33	1,23	1,16E-04	9,15E-04	0,56
1245,55	21,69	Collagen alpha-1(II) chain	80(2637)	92	111(3084)	96	0,72	4,00E-03	1,44E-02	0,54
1250,56	28	Collagen alpha-1(I) chain	36626(188)	35	44802(131)	27	0,82	3,00E-12	6,81E-10	0,63
1257,44	33,56	Heterogeneous nuclear ribonucleoprotein U	2898(2052)	85	3777(1855)	90	0,77	2,45E-03	9,75E-03	0,56
1260,56	21,9	Collagen alpha-1(III) chain	291(1227)	88	447(1353)	96	0,65	8,75E-06	1,18E-04	0,58
1268,53	21,43	Collagen alpha-3(IV) chain	157(1543)	84	210(1710)	74	0,75	4,80E-03	1,67E-02	0,55
1270,55	29,29	Collagen alpha-1(I) chain	46(1732)	46	77(2522)	61	0,60	1,56E-05	1,82E-04	0,56
1283,55	27,39	Collagen alpha-2(I) chain	69(258)	45	99(378)	56	0,70	9,57E-05	7,91E-04	0,56
1286,54	29,33	Collagen alpha-1(I) chain	36(175)	33	59(199)	43	0,61	9,27E-06	1,24E-04	0,57
1287,59	21,92	Apolipoprotein A-IV	330(342)	50	554(468)	68	0,60	1,77E-10	2,46E-08	0,61
1304,61	22	Collagen alpha-1(XXVI) chain	120(269)	56	198(343)	67	0,61	3,89E-05	3,90E-04	0,57
1312,55	29,69	Titin	1445(426)	43	1939(562)	49	0,75	8,24E-10	6,67E-08	0,61
1321,59	28,38	Collagen alpha-1(I) chain	11305(10867)	86	14846(10508)	97	0,76	2,95E-07	9,21E-06	0,59
1336,58	21,56	Collagen alpha-1(IV) chain	138(815)	82	104(560)	78	1,33	1,09E-03	5,19E-03	0,55
1350,57	21,29		22(166)	41	64(211)	49	0,35	8,45E-13	2,39E-10	0,60
1350,62	27,21	Mucin-4	343(145)	24	442(248)	35	0,77	1,07E-07	4,32E-06	0,60

CE-MS characteristics			Biomarker panel					Statistical analysis		
			Distribution in cancer cases (n=676)		Distribution in cancer controls (n=744)		Fold change (Mean Amp cases/Mean Amp controls)			
Mass [Da]	CE Migration time (min)	Protein name	Mean Amp (SD)	Frequency	Mean Amp (SD)	Frequency		p value I	p value II (FDR adj. P)	AUC
1351,63	38,77	Collagen alpha-1(I) chain	238(211)	41	148(188)	31	1,60	1,32E-04	1,00E-03	0,57
1352,79	24,82	Mucin-16	166(9693)	91	253(11319)	94	0,65	1,36E-04	1,00E-03	0,56
1361,63	22,09	Sodium/potassium-transporting ATPase subunit alpha-4	315(1702)	40	465(181)	30	0,68	7,68E-03	2,45E-02	0,55
1378,61	28,89	Collagen alpha-1(I) chain	2716(617)	87	3299(494)	80	0,82	3,22E-07	9,28E-06	0,59
1383,63	38,9	Ras and Rab interactor 3	214(4770)	18	66(7423)	33	3,25	1,02E-04	8,26E-04	0,56
1390,44	36,82		27064(351)	39	34359(468)	49	0,79	7,03E-03	2,27E-02	0,55
1396,62	26,63	Collagen alpha-1(III) chain	75(4293)	69	99(6392)	75	0,76	3,72E-04	2,16E-03	0,56
1407,64	27,55	Collagen alpha-1(I) chain	160(205)	48	245(277)	57	0,66	6,89E-06	9,52E-05	0,58
1408,65	39,07	Collagen alpha-1(I) chain	636(142)	45	437(95)	30	1,46	1,25E-04	9,66E-04	0,57
1417,64	20,06	Collagen alpha-2(I) chain	688(4625)	92	947(4918)	95	0,73	7,34E-05	6,50E-04	0,57
1422,53	37,7	Ankyrin repeat domain 17	599(453)	61	897(661)	65	0,67	1,89E-08	1,04E-06	0,60
1423,64	37,35	Fibrinogen alpha chain	130(2759)	54	63(1204)	42	2,06	8,98E-08	3,91E-06	0,58
1425,59	22,34		3146(3560)	96	3923(3918)	98	0,80	1,63E-04	1,14E-03	0,57
1438,67	28,06	Collagen alpha-1(III) chain	3302(1039)	28	4178(3833)	37	0,79	4,49E-07	1,08E-05	0,59
1462,61	39,5	Collagen alpha-3(V) chain	545(828)	69	932(819)	81	0,58	1,47E-04	1,05E-03	0,56
1485,68	23,77	Collagen alpha-1(I) chain	1219(1581)	47	1492(1670)	61	0,82	3,41E-07	9,28E-06	0,59
1495,68	39,45	Collagen alpha-1(I) chain	104(1443)	55	67(1455)	64	1,55	2,67E-03	1,04E-02	0,55
1505,61	28,85	Nuclear receptor corepressor 1	149(1326)	89	281(1570)	95	0,53	3,01E-07	9,21E-06	0,58
1524,65	20,03	Collagen alpha-1(XI) chain	455(99)	34	606(98)	43	0,75	5,33E-03	1,83E-02	0,55
1525,55	21,83		190(377)	27	306(504)	43	0,62	1,37E-03	6,23E-03	0,55
1552,5	39,02		239(2932)	85	300(4090)	89	0,80	2,64E-04	1,63E-03	0,56
1560,57	21,53		100(1163)	33	209(1591)	42	0,48	2,32E-05	2,48E-04	0,56
1564,72	28,29	Collagen alpha-1(V) chain	42(1230)	80	56(1047)	76	0,75	1,62E-03	7,07E-03	0,55
1594,76	40,28	Collagen alpha-1(I) chain	1372(315)	34	1044(167)	22	1,31	4,82E-07	1,14E-05	0,59
1610,88	30,35		1029(356)	72	705(243)	61	1,46	3,44E-04	2,04E-03	0,56

CE-MS characteristics			Biomarker panel					Statistical analysis		
			Distribution in cancer cases (n=676)		Distribution in cancer controls (n=744)		Fold change (Mean Amp cases/Mean Amp controls)			
Mass [Da]	CE Migration time (min)	Protein name	Mean Amp (SD)	Frequency	Mean Amp (SD)	Frequency		p value I	p value II (FDR adj. P)	AUC
1620,7	22,69	Collagen alpha-2(I) chain	141(18543)	98	225(21590)	99	0,63	6,57E-05	5,91E-04	0,56
1640,6	23,02		3567(1376)	78	5741(1473)	82	0,62	1,26E-07	4,93E-06	0,60
1651,79	40,75	Collagen alpha-1(I) chain	4598(96)	33	3442(79)	23	1,34	2,12E-10	2,46E-08	0,62
1684,66	30,88	Collagen alpha-1(I) chain	1661(525)	32	2980(596)	41	0,56	1,82E-11	3,43E-09	0,62
1703,83	33,54	Collagen alpha-3(V) chain	160(1625)	28	410(996)	37	0,39	1,66E-05	1,90E-04	0,57
1716,65	20,16		234(3672)	71	373(4285)	78	0,63	5,26E-03	1,81E-02	0,55
1720,69	19,64		54(319)	59	106(165)	55	0,51	3,83E-05	3,87E-04	0,56
1732,77	28,3		2174(1032)	65	2696(1422)	72	0,81	1,69E-04	1,16E-03	0,57
1734,66	19,83		77(184)	40	129(245)	52	0,60	1,63E-04	1,14E-03	0,56
1764,68	19,92		167(137)	31	261(162)	40	0,64	7,88E-06	1,08E-04	0,57
1767	24,9		249(470)	69	451(651)	83	0,55	6,41E-03	2,11E-02	0,54
1767,78	19,8	Fibrinogen alpha chain	227(465)	52	137(468)	36	1,66	3,77E-07	9,74E-06	0,58
1793,89	32,41	Fibrinogen beta chain	644(1912)	96	505(6442)	96	1,27	2,25E-04	1,46E-03	0,57
1807,81	20,72	Collagen alpha-1(I) chain	2074(3133)	95	2645(3144)	98	0,78	1,32E-02	3,70E-02	0,55
1809,87	32,43	Acyl-protein thioesterase 2	265(475)	66	163(583)	71	1,62	5,87E-06	8,52E-05	0,57
1817,7	20,25	Stabilin-2	2637(368)	42	3950(503)	51	0,67	3,78E-07	9,74E-06	0,59
1828,85	21,21	Collagen alpha-1(I) chain	165(4898)	30	107(5118)	44	1,54	1,81E-02	4,76E-02	0,54
1835,71	19,87		663(262)	41	1049(330)	50	0,63	3,87E-07	9,74E-06	0,59
1858,84	24,34	Prostaglandin-H2 D-isomerase	157(34)	18	304(61)	33	0,52	1,05E-05	1,36E-04	0,57
1878,59	31,17		695(557)	29	904(1516)	40	0,77	1,41E-04	1,02E-03	0,57
1885,87	21,25	Collagen alpha-1(I) chain	88(1150)	50	53(1250)	58	1,66	1,11E-02	3,25E-02	0,54
1889,86	33,19	Collagen alpha-2(IV) chain	142(7725)	35	69(6887)	24	2,05	1,12E-05	1,41E-04	0,57
1892,87	22,13	Collagen alpha-1(I) chain	599(484)	51	454(555)	60	1,32	3,25E-04	1,95E-03	0,57
1893,04	29,05	26S protease regulatory subunit 10B	184(1141)	67	263(913)	62	0,70	6,42E-06	9,10E-05	0,58
1899,81	22,44		80(2404)	46	106(1816)	32	0,75	6,20E-05	5,62E-04	0,56

CE-MS characteristics			Biomarker panel					Statistical analysis		
			Distribution in cancer cases (n=676)		Distribution in cancer controls (n=744)		Fold change (Mean Amp cases/Mean Amp controls)			
Mass [Da]	CE Migration time (min)	Protein name	Mean Amp (SD)	Frequency	Mean Amp (SD)	Frequency		p value I	p value II (FDR adj. P)	AUC
1911,05	25,23		79331(1478)	73	103725(1000)	68	0,76	1,08E-05	1,38E-04	0,58
1915	33,62	Metastasis suppressor protein 1	83(449)	36	49(263)	24	1,70	3,08E-03	1,17E-02	0,55
1916,78	20,22		1155(8075)	89	1608(7772)	91	0,72	6,67E-05	5,95E-04	0,57
1933,88	21,64	Collagen alpha-1(I) chain	964(677)	67	752(780)	75	1,28	5,41E-03	1,83E-02	0,55
1934,79	19,91	Keratin-1	340(108)	25	534(138)	35	0,64	2,47E-06	4,49E-05	0,58
1945	33,66	Glycogen phosphorylase, brain form	288(112)	39	214(97)	29	1,34	1,10E-03	5,21E-03	0,56
1991,94	22,05	Collagen alpha-1(III) chain	1815(368)	41	1021(287)	27	1,78	5,22E-06	7,97E-05	0,58
1996,78	20,96		519(269)	24	842(389)	34	0,62	1,29E-09	9,12E-08	0,61
2064,92	24,43	Collagen alpha-1(II) chain	730(244)	31	549(221)	41	1,33	5,14E-05	4,89E-04	0,57
2067,82	20,6	Microtubule-associated protein tau	1088(2169)	25	1491(2375)	35	0,73	7,42E-04	3,88E-03	0,56
2093,93	33,71	Collagen alpha-2(I) chain	302(243)	37	167(274)	26	1,80	2,35E-07	7,90E-06	0,59
2103,96	33,08	Collagen alpha-1(I) chain	2759(872)	37	2308(488)	26	1,20	1,05E-03	5,08E-03	0,56
2145,93	48,5	Nidogen-2	10877(299)	38	18165(289)	29	0,60	2,61E-10	2,46E-08	0,61
2152,97	32,92		66(770)	29	35(1771)	39	1,89	1,63E-05	1,88E-04	0,57
2170,97	27,53	Collagen alpha-1(I) chain	277(121)	43	400(129)	53	0,69	1,45E-05	1,75E-04	0,57
2184,57	35,06		362(278)	40	552(254)	31	0,66	3,38E-08	1,63E-06	0,60
2187,96	39,54	Mucin-12	1465(103)	29	1980(135)	49	0,74	9,49E-04	4,70E-03	0,56
2199	22,33	Collagen alpha-1(I) chain	104(592)	68	298(859)	79	0,35	4,69E-03	1,64E-02	0,54
2264,94	43,13	Collagen alpha-2(IV) chain	238(690)	31	94(845)	43	2,54	4,96E-04	2,78E-03	0,55
2272,24	23,91	Histone deacetylase 6	227(816)	42	131(946)	52	1,73	2,68E-06	4,75E-05	0,58
2306,04	19,52	Fibrinogen alpha chain	1194(322)	23	313(304)	33	3,82	2,26E-06	4,20E-05	0,57
2321,19	22,06	Fibrinogen beta chain	378(2271)	41	195(1725)	51	1,94	1,03E-07	4,31E-06	0,59
2356,66	35,6		485(2078)	90	609(2567)	90	0,80	1,31E-04	9,93E-04	0,57
2479,06	47,73	Mucin-22	727(134)	26	1281(257)	40	0,57	8,06E-09	5,07E-07	0,60

CE-MS characteristics			Biomarker panel					Statistical analysis		
			Distribution in cancer cases (n=676)		Distribution in cancer controls (n=744)		Fold change (Mean Amp cases/Mean Amp controls)			
Mass [Da]	CE Migration time (min)	Protein name	Mean Amp (SD)	Frequency	Mean Amp (SD)	Frequency		p value I	p value II (FDR adj. P)	AUC
2548	21,36		282(686)	68	376(859)	76	0,75	8,55E-04	4,35E-03	0,56
2565,15	23,6	Collagen alpha-1(XVII) chain	248(492)	51	188(377)	43	1,32	6,42E-04	3,50E-03	0,55
2583,2	28,3	Collagen alpha-1(I) chain	790(3794)	62	588(2369)	54	1,34	1,82E-04	1,21E-03	0,57
2600,23	35,2	Collagen alpha-1(I) chain	44(364)	61	27(307)	49	1,62	1,57E-03	6,96E-03	0,55
2647,21	23,52	Collagen alpha-1(III) chain	94(182)	41	51(171)	32	1,84	1,95E-05	2,14E-04	0,57
2663,21	23,57	Collagen alpha-1(III) chain	934(203)	45	652(407)	37	1,43	1,45E-07	5,49E-06	0,60
2734,29	27,99		121(1321)	80	67(1165)	71	1,80	1,18E-03	5,49E-03	0,55
2739,23	28,46	Chloride intracellular channel protein 6	145(180)	46	108(106)	35	1,34	1,65E-03	7,13E-03	0,56
2751,34	29,11		238(386)	55	309(561)	60	0,77	8,83E-04	4,45E-03	0,56
2808,3	24,33		444(7276)	73	230(6286)	81	1,93	4,57E-06	7,50E-05	0,57
2834,19	22,35		44(96)	23	104(98)	32	0,43	1,15E-10	1,86E-08	0,60
2854,37	34,67	Collagen alpha-1(III) chain	3628(1093)	41	4551(1197)	28	0,80	9,64E-05	7,91E-04	0,57
2861,29	48,8	Protein transport protein Sec31A	531(218)	34	852(127)	24	0,62	2,11E-07	7,47E-06	0,59
2887,35	35,57	Collagen alpha-2(V) chain	283(408)	68	198(326)	61	1,43	4,45E-03	1,57E-02	0,55
2903,35	35,64	Collagen alpha-2(I) chain	68(319)	54	36(334)	43	1,88	2,76E-05	2,86E-04	0,57
2907,35	35,85	Collagen alpha-2(I) chain	287(836)	76	174(785)	71	1,65	3,17E-06	5,51E-05	0,58
2946,2	35,07		86(963)	64	50(682)	53	1,72	4,14E-05	4,04E-04	0,57
2959,36	48,32	Collagen alpha-1(III) chain	169(78)	26	277(99)	37	0,61	2,92E-05	3,00E-04	0,56
2973,46	24,38	Collagen alpha-1(I) chain	571(353)	54	367(304)	42	1,55	2,19E-05	2,36E-04	0,58
2989,44	24,47	Collagen alpha-1(I) chain	310(695)	40	165(658)	27	1,88	2,49E-06	4,49E-05	0,58
2999,3	22,24	Collagen alpha-1(I) chain	2498(396)	49	1860(438)	57	1,34	1,60E-03	7,05E-03	0,56
3017,4	48,54	Collagen alpha-2(V) chain	1590(453)	63	2399(611)	76	0,66	5,39E-06	8,04E-05	0,57
3021,38	23,21	Collagen alpha-1(XI) chain	983(236)	42	1200(146)	34	0,82	9,29E-03	2,81E-02	0,55
3023,41	20,91	Collagen alpha-1(III) chain	224(102)	26	163(181)	44	1,37	3,51E-04	2,06E-03	0,56
3025,4	29,89	Collagen alpha-2(I) chain	144(13665)	80	94(15637)	81	1,53	1,22E-04	9,46E-04	0,57

CE-MS characteristics			Biomarker panel					Statistical analysis		
			Distribution in cancer cases (n=676)		Distribution in cancer controls (n=744)					
Mass [Da]	CE Migration time (min)	Protein name	Mean Amp (SD)	Frequency	Mean Amp (SD)	Frequency	Fold change (Mean Amp cases/Mean Amp controls)	p value I	p value II (FDR adj. P)	AUC
3031,44	35,84	Collagen alpha-1(I) chain	248(1185)	72	186(4564)	64	1,34	9,92E-04	4,83E-03	0,56
3039,39	24,6	Collagen alpha-2(IV) chain	68(1485)	36	49(1729)	52	1,40	3,54E-03	1,31E-02	0,54
3057,39	29,96	Collagen alpha-2(V) chain	103(778)	40	76(539)	27	1,36	5,69E-03	1,89E-02	0,54
3091,46	28,24		399(192)	39	545(170)	30	0,73	3,58E-03	1,32E-02	0,55
3092,44	36,22	Collagen alpha-2(I) chain	258(2752)	97	193(2528)	95	1,34	8,74E-03	2,70E-02	0,55
3158,42	29,8	Collagen alpha-1(XI) chain	1166(1104)	36	842(694)	26	1,38	2,99E-03	1,15E-02	0,55
3168,37	24,55	Collagen alpha-1(III) chain	689(551)	44	507(1271)	33	1,36	7,42E-04	3,88E-03	0,56
3184,36	24,71	Collagen alpha-1(V) chain	252(3006)	64	141(2555)	56	1,78	6,75E-10	5,89E-08	0,60
3194,43	30,28	Collagen alpha-1(III) chain	146(2336)	65	76(2458)	72	1,92	1,07E-05	1,38E-04	0,56
3261,53	22,19		183(3569)	88	132(4092)	92	1,39	1,68E-05	1,90E-04	0,57
3265,44	35,9	Collagen alpha-1(XI) chain	671(419)	33	368(224)	22	1,82	6,88E-08	3,12E-06	0,59
3281,43	35,96	Collagen alpha-1(XI) chain	2373(18908)	68	1798(6722)	58	1,32	5,14E-06	7,97E-05	0,58
3288,52	36,49	Receptor-type tyrosine-protein phosphatase U	103(284)	40	58(183)	30	1,76	1,83E-05	2,03E-04	0,57
3290,54	23,89	Collagen alpha-2(V) chain	474(9987)	92	651(10436)	95	0,73	1,66E-02	4,41E-02	0,54
3298,46	35,95	Collagen alpha-1(I) chain	358(131)	39	177(133)	27	2,03	1,82E-07	6,66E-06	0,58
3313,54	25,62	Collagen alpha-2(I) chain	129(354)	42	75(306)	28	1,72	7,30E-04	3,88E-03	0,55
3318,52	30,97	Collagen alpha-1(I) chain	121(969)	85	90(760)	80	1,34	1,64E-02	4,38E-02	0,54
3356,52	25,46	Collagen alpha-1(I) chain	187(305)	36	101(141)	25	1,86	8,27E-07	1,67E-05	0,58
3385,59	25,54		2618(680)	40	1972(974)	30	1,33	1,89E-03	7,97E-03	0,56
3401,61	25,47	Collagen alpha-1(V) chain	1275(1454)	55	972(3379)	68	1,31	9,23E-05	7,80E-04	0,57
3439,59	38,9	Collagen alpha-1(XII) chain	117(394)	62	97(250)	50	1,21	1,16E-03	5,39E-03	0,55
3489,58	31,42		591(5387)	41	489(1139)	28	1,21	1,23E-02	3,49E-02	0,54
3546,67	26,15		173(450)	61	72(653)	66	2,40	6,19E-07	1,34E-05	0,57
3582,75	19,46		2313(218)	52	1469(151)	44	1,57	8,22E-05	7,02E-04	0,57
3583,63	26,32	Collagen alpha-1(III) chain	111(167)	43	76(134)	34	1,47	6,82E-06	9,52E-05	0,57

CE-MS characteristics			Biomarker panel					Statistical analysis		
			Distribution in cancer cases (n=676)		Distribution in cancer controls (n=744)		Fold change (Mean Amp cases/Mean Amp controls)			
Mass [Da]	CE Migration time (min)	Protein name	Mean Amp (SD)	Frequency	Mean Amp (SD)	Frequency		p value I	p value II (FDR adj. P)	AUC
3593,47	20,19	Collagen alpha-1(XI) chain	229(1825)	28	307(1087)	40	0,75	6,43E-04	3,50E-03	0,55
3618,67	31,67	Collagen alpha-1(I) chain	110(256)	67	58(240)	60	1,89	3,25E-04	1,95E-03	0,55
3681,71	31,87		92(2612)	84	60(2064)	81	1,52	1,50E-03	6,72E-03	0,55
3710,76	19,57	Collagen alpha-1(XVI) chain	245(2211)	87	142(1970)	88	1,72	1,11E-04	8,80E-04	0,56
3822,11	24,81		201(1957)	83	352(2359)	89	0,57	2,16E-03	8,72E-03	0,55
3943,83	33,54	Collagen alpha-1(III) chain	214(12290)	59	170(10387)	76	1,25	3,30E-03	1,23E-02	0,55
3968,63	21,07		1481(3676)	91	1938(3036)	91	0,76	1,23E-05	1,50E-04	0,58
4043,65	20,16		753(575)	26	933(1150)	38	0,81	4,13E-05	4,04E-04	0,57
4059,65	20,38		243(2442)	34	111(2812)	22	2,20	5,08E-06	7,97E-05	0,56
4078,81	33,06	Collagen alpha-3(IV) chain	246(491)	55	123(317)	41	2,00	5,95E-07	1,34E-05	0,58
4185,91	33,6		67(194)	61	50(156)	54	1,34	1,53E-04	1,08E-03	0,56
4196,78	20,85		182(215)	29	234(287)	38	0,78	3,49E-04	2,06E-03	0,56
4368,87	20,21	Collagen alpha-1(XI) chain	1140(2340)	36	521(2120)	22	2,19	1,18E-05	1,46E-04	0,57
4404,87	20,59		643(299)	29	859(416)	40	0,75	7,54E-05	6,59E-04	0,57
4654,14	25,78	Collagen alpha-1(III) chain	3018(527)	35	2253(416)	24	1,34	5,42E-03	1,83E-02	0,55
4817,17	23,74	Collagen alpha-1(II) chain	847(759)	32	431(372)	17	1,96	1,02E-05	1,35E-04	0,56
4833,16	23,83	Collagen alpha-1(I) chain	794(849)	60	436(962)	48	1,82	4,63E-06	7,50E-05	0,57
4960,48	20,57	Collagen alpha-1(II) chain	4215(392)	39	2056(420)	46	2,05	5,69E-03	1,89E-02	0,54
5510,35	26,99		336(557)	59	520(686)	72	0,65	1,53E-05	1,80E-04	0,58
6211,71	20,26		256(4180)	76	443(4643)	84	0,58	7,03E-07	1,47E-05	0,58
6236,85	21,06		4582(183)	47	2598(165)	40	1,76	6,20E-07	1,34E-05	0,59
9866,38	20,83		2139(1199)	79	2699(1688)	74	0,79	5,95E-05	5,48E-04	0,57
9882,83	20,82		382(40282)	78	147(26267)	69	2,60	2,37E-07	7,90E-06	0,57
10999,63	21,33		913(81)	27	1426(108)	36	0,64	3,16E-07	9,28E-06	0,57

***** Abbreviations: Amp, amplitude; CE-MS, Capillary Electrophoresis coupled to Mass Spectrometry; Da,Dalton; min, minutes; SD: Standard deviation; FDR adj., False Discovery Rate adjustment.

Supplementary Table S2. Investigation of the distribution of the 193 peptide markers in individual cancers. Statistical analyses were performed in the discovery set using the Wilcoxon rank sum test for each cancer group separately, followed by adjustment using Benjamini-Hochberg method. Each analysis was repeated ten times by excluding randomly 30% of the samples in both groups and applying the frequency threshold of 70% (at least in one group). The results of the analysis are presented in the table. Adjusted p-values of the Wilcoxon rank sum test are reported. Non-significant peptides are reported as n.s. N/A denotes peptides that were not commonly detected in all 10 permutations with at least 70% frequency. Peptides that were observed as statistically significant in the discovery set of one cancer type (adj. p-value highlighted in red) were considered for further analysis in the validation set (unadjusted p-values of Wilcoxon rank sum test are reported). Only those peptides that were observed as statistically significant in both discovery and validation sets are considered as cancer-type specific peptides (17 peptides highlighted in grey color).

193-peptide marker pattern			Bladder cancer				Prostate cancer				Cholangiocarcinoma				Pancreatic cancer				Renal cell carcinoma			
			Discovery set		Validation set		Discovery set		Validation set		Discovery set		Validation set		Discovery set		Validation set		Discovery set		Validation set	
Mass [Da]	CE migration time [min]	Protein name	adj. P-value	Fold change (case/control)	unadj. P-value	Fold change (case/control)	adj. P-value	Fold change (case/control)	unadj. P-value	Fold change (case/control)	adj. P-value	Fold change (case/control)	unadj. P-value	Fold change (case/control)	adj. P-value	Fold change (case/control)	unadj. P-value	Fold change (case/control)	adj. P-value	Fold change (case/control)	unadj. P-value	Fold change (case/control)
1129,46	27,75	Collagen alpha-1(VII) chain	N/A	0,71		1,88	0,03	0,68	0,01	0,70	N/A	1,84		1,38	N/A	0,48		1,48	N/A	0,59		0,35
1878,59	31,17		N/A	0,44		1,69	0,02	0,82	0,02	0,74	N/A	0,41		0,34	N/A	0,29		0,32	N/A	0,42		0,56
2184,57	35,06		N/A	0,62		0,78	0,04	0,74	0,03	0,76	N/A	0,40		0,56	N/A	0,40		1,06	N/A	0,57		0,78
2103,96	33,08	Collagen alpha-1(I) chain	n.s	0,92		1,11	0,00	1,21	0,02	1,25	n.s	1,18		1,22	n.s	1,21		1,02	n.s	1,26		1,35
1099,49	28,06	Sodium/potassium-transporting ATPase subunit gamma	N/A	1,16		1,40	0,01	0,75	0,00	0,69	n.s	1,39		1,04	n.s	0,81		1,53	n.s	0,68		0,62
1417,64	20,06	Collagen alpha-2(I) chain	n.s	1,03		1,49	n.s	0,87		0,91	0,01	0,56	0,01	0,62	n.s	0,80		0,83	n.s	0,70		0,28
1640,60	23,02		N/A	0,59		0,69	n.s	1,19		1,06	0,01	0,70	0,00	0,36	n.s	0,59		1,18	N/A	0,57		0,37
1807,81	20,72	Collagen alpha-1(I) chain	n.s	0,85		0,71	n.s	0,86		0,80	0,01	0,71	0,02	0,82	n.s	0,59		0,56	n.s	0,80		0,52
1835,71	19,87		N/A	0,84		0,80	N/A	0,87		0,69	0,00	0,61	0,00	0,27	N/A	0,83		1,58	n.s	0,55		0,18
1934,79	19,91	Keratin-1	N/A	1,14		0,72	N/A	0,88		0,63	0,00	0,47	0,00	0,24	N/A	1,28		1,81	N/A	0,59		0,24
1916,78	20,22		n.s	0,87		0,80	n.s	0,73		0,80	0,00	0,56	0,00	0,27	N/A	1,13		1,68	n.s	0,87		0,40
2067,82	20,60	Microtubule-associated protein tau	n.s	1,01		0,84	n.s	0,91		0,79	0,01	0,60	0,00	0,28	n.s	1,21		1,34	n.s	0,76		0,40
2145,93	48,50	Nidogen-2	N/A	0,25		1,23	N/A	0,81		1,00	0,00	0,53	0,02	0,69	n.s	0,74		0,37	N/A	0,48		0,26
9866,38	20,83		n.s	0,69		0,40	n.s	1,13		1,18	0,00	0,39	0,02	0,60	n.s	0,66		0,67	n.s	1,07		0,39
3968,63	21,07		N/A	0,78		0,41	N/A	1,27		1,27	0,01	0,70	0,00	0,37	N/A	1,16		1,47	N/A	0,67		0,65
1361,63	22,09	Sodium/potassium-transporting ATPase subunit alpha-4	N/A	1,12		1,26	N/A	1,19		1,24	0,03	0,63	0,00	0,43	n.s	0,55		1,06	N/A	0,70		0,41
3168,37	24,55	Collagen alpha-1(III) chain	n.s	2,01		1,39	n.s	1,37		1,45	n.s	1,07		1,73	n.s	1,18		1,04	0,02	2,25	0,04	2,35
884,32	24,72	Cadherin-13	N/A	1,35		1,71	0,01	0,54	n.s	0,99	n.s	2,59		2,75	N/A	0,71		1,38	n.s	1,33		0,65
1084,43	25,18	Collagen alpha-1(II) chain	n.s	0,96		0,95	0,01	0,69	n.s	0,79	n.s	1,28		1,29	n.s	0,62		1,16	n.s	0,85		0,45
1113,47	27,24	14-3-3 protein sigma	N/A	0,56		1,31	0,02	0,70	n.s	0,77	N/A	1,69		1,03	N/A	0,42		0,41	n.s	0,70		0,60
1257,44	33,56	Heterogeneous nuclear ribonucleoprotein U	N/A	0,97		1,29	0,02	0,73	n.s	0,81	n.s	1,32		1,54	n.s	0,70		1,51	n.s	0,68		0,37
2356,66	35,60		N/A	0,56		1,05	0,02	0,79	n.s	0,82	N/A	0,40		0,59	N/A	0,48		0,31	n.s	0,56		0,58
1350,62	27,21	Mucin-4	n.s	0,75		1,24	0,02	0,70	n.s	0,92	N/A	0,83		0,88	N/A	1,07		1,01	n.s	0,80		0,52
3281,43	35,96	Collagen alpha-1(XI) chain	n.s	0,98		0,75	0,03	1,54	n.s	1,62	n.s	0,69		1,10	n.s	0,89		0,89	n.s	1,53		1,46
1438,67	28,06	Collagen alpha-1(III) chain	n.s	0,77		0,91	n.s	0,88		0,82	0,02	0,78	n.s	1,23	n.s	0,90		0,85	n.s	0,90		1,12
1684,66	30,88	Collagen alpha-1(I) chain	N/A	0,40		3,50	N/A	0,95		1,08	0,03	0,80	n.s	0,90	n.s	0,79		1,59	N/A	0,30		0,36
1793,89	32,41	Fibrinogen beta chain	n.s	1,43		1,62	n.s	1,15		1,14	n.s	1,16		0,65	0,00	0,90	n.s	0,96	n.s	1,42		2,45
2999,30	22,24	Collagen alpha-1(I) chain	n.s	1,35		0,77	n.s	1,31		0,88	n.s	1,16		1,16	0,01	1,61	n.s	1,00	n.s	1,78		1,43
980,29	35,64		N/A	4,12		1,29	N/A	1,37		1,93	N/A	0,62		0,63	0,02	0,52	n.s	1,33	N/A	1,21		1,19
1809,87	32,43	Acyl-protein thioesterase 2	N/A	1,92		6,06	N/A	2,16		4,01	N/A	1,91		1,52	0,01	1,20	n.s	0,76	N/A	6,88		12,66
2321,19	22,06	Fibrinogen beta chain	N/A	3,57		2,56	N/A	1,13		0,78	N/A	2,18		1,37	0,01	1,65	n.s	0,38	N/A	2,48		4,42
2479,06	47,73	Mucin-22	N/A	0,46		0,91	N/A	1,04		1,18	N/A	0,59		0,70	0,00	0,27	n.s	0,18	N/A	0,48		0,36
3021,38	23,21	Collagen alpha-1(XI) chain	N/A	0,72		0,37	N/A	1,18		0,62	N/A	0,51		0,60	0,00	0,31	n.s	0,76	N/A	0,77		0,82
2064,92	24,43	Collagen alpha-1(II) chain	n.s	1,19		1,54	n.s	0,91		1,15	n.s	0,93		1,62	n.s	0,83		0,74	0,00	1,82	n.s	1,55
1732,77	28,30		n.s	1,12		1,39	n.s	0,96		0,95	n.s	0,80		0,50	N/A	0,94		1,03	0,02	0,61	n.s	1,02
3091,46	28,24		n.s	0,82		0,42	n.s	0,81		0,77	N/A	0,45		0,46	N/A	0,51		0,52	0,01	0,47	n.s	0,94
2907,35	35,85	Collagen alpha-2(I) chain	n.s	1,52		0,91	N/A	1,39		1,68	N/A	0,92		0,65	n.s	1,08		0,78	0,02	2,28	n.s	1,39
928,37	35,53	Collagen alpha-5(VI) chain	N/A	0,86		1,37	N/A	0,84		0,66	N/A	2,27		0,59	N/A	1,20		0,94	N/A	0,43		0,23
949,22	34,54		N/A	0,97		1,07	N/A	0,76		0,91	N/A	1,26		1,14	N/A	0,41		0,62	N/A	0,87		0,07
956,45	20,35	Mucin-12	N/A	3,84		1,38	N/A	0,57		1,36	n.s	0,93		1,32	N/A	0,85		1,26	N/A	0,59		0,16
992,42	20,37	PHD finger protein 10	N/A	2,16		0,25	N/A	0,78		0,97	N/A	0,48		0,49	N/A	0,68		2,30	N/A	0,46		0,09
1007,31	35,68		N/A	3,92		1,48	N/A	1,51		2,26	N/A	1,13		0,57	N/A	0,42		1,08	N/A	0,74		0,72
1013,37	25,06	Inositol 1,4,5-trisphosphate receptor type 2	n.s	1,12		1,14	0,00	0,66		0,73	n.s	1,47		1,81	0,02	0,57		0,63	n.s	0,81		0,38
1060,38	38,03		N/A	0,79		0,81	N/A	0,59		0,33	N/A	1,27		1,13	N/A	1,01		1,57	N/A	0,35		0,34
1069,48	25,33	Collagen alpha-1(III) chain	N/A	0,56		0,68	N/A	0,71		0,81	N/A	0,83		1,36	N/A	0,69		1,27	N/A	1,48		0,32
1075,49	20,68	Myeloperoxidase	N/A	1,45		0,27	N/A	1,12		1,50	N/A	0,39		0,43	N/A	0,59		0,32	N/A	0,43		0,11
1114,48	24,04	Collagen alpha-1(III) chain	N/A	0,82		1,01	N/A	0,78		0,82	N/A	0,69		0,70	N/A	0,44		0,52	N/A	0,67		0,25
1115,51	21,60	Collagen alpha-1(III) chain	N/A	2,60		7,82	N/A	1,36		1,48	N/A	0,77		0,98	N/A	0,98		0,62	N/A	2,94		0,79
1128,4	33,10		N/A	0,69		1,15	N/A	0,57		0,83	N/A	1,61		1,98	N/A	1,08		1,46	N/A	0,45		0,23
1161,5	37,60	Collagen alpha-1(XV) chain	N/A	2,60		5,75	N/A	0,65		0,89	N/A	2,08		3,52	N/A	0,98		1,60	N/A	0,76		0,77
1178,38	20,44	GC-rich sequence DNA-binding factor 1	N/A	1,06		0,58	N/A	1,20		0,72	N/A	0,55		0,31	N/A	0,48		0,74	N/A	0,30		0,04
1181,48	36,80	Collagen alpha-1(I) chain	N/A	0,73		1,42	N/A	0,68		1,13	N/A	1,56		1,14	N/A	1,00		1,68	N/A	0,21		0,16
1182,55	28,20	Collagen alpha-3(IV) chain	N/A	1,34		4,27	N/A	0,68		0,50	N/A	0,74		0,77	N/A	0,85		1,01	N/A	0,19		0,24
1208,38	45,14		N/A	0,93		2,74	N/A	0,68		0,41	N/A	0,90		1,14	N/A	0,83		2,40	N/A	0,12		0,08
1215,44	26,94		N/A	0,48		0,76	N/A	0,77		0,76	N/A	1,08		0,36	N/A	1,10		1,39	N/A	0,46		0,37
1216,54	24,59		n.s	1,12		1,83	n.s	0,84		0,88	0,02	0,80		0,71	n.s	0,77		1,34	0,04	0,53		0,29
1217,48	27,78	E3 ubiquitin-protein ligase UBR2	N/A	0,45		0,97	N/A	0,91		1,31	N/A	0,96		0,26	N/A	0,78		2,42	N/A	0,71		0,39
1224,36	45,35		N/A	1,63		1,53	N/A	0,56		0,28	N/A	1,87		0,92	N/A	0,88		1,72	N/A	0,00		0,12
1226,53	21,11		N/A	1,04		1,25	0,00	0,66		0,66	0,03	0,68		0,71	0,03	0,52		0,48	N/A	0,59		0,20
1232,52	38,06	Collagen alpha-1(II) chain	N/A	5,36		6,94	N/A	1,12		0,40	N/A	1,71		2,55	N/A	1,47		1,53	N/A	1,86		0,24
1239,43	33,58	Myb-binding protein 1A	N/A	1,00		0,75	N/A	1,01		1,14	N/A	1,16		1,77	N/A	0,37		1,32	N/A	0,82		0,48
1245,55	21,69	Collagen alpha-1(II) chain	N/A	1,15		1,10	N/A	0,51		1,63	N/A	1,01		1,09	N/A	1,09		1,40	N/A	1,28		0,30
1250,56	28,00	Collagen alpha-1(I) chain	n.s	0,90		1,24	0,00	0,82		0,78	n.s	1,10		0,90	n.s	0,95		1,02	0,01	0,69		0,60
1260,56	21,90	Collagen alpha-1(III) chain	N/A	0,75		0,90	N/A	0,50		0,66	N/A	0,90		0,79	N/A	0,55		1,97	N/A	0,82		0,67
1268,53	21,43	Collagen alpha-3(IV) chain	N/A	1,34		1,16	N/A	0,53		0,28	N/A	0,76		0,64	N/A	0,62		0,89	N/A	1,25		0,86
1270,55	29,29	Collagen alpha-1(I) chain	N/A	0,84		4,37	N/A	0,52		0,25	N/A	1,05		1,04	N/A	0,65		1,30	N/A	0,48		0,00
1283,55	27,39	Collagen alpha-2(I) chain	N/A	0,74		1,58	N/A	0,59		0,44	N/A	1,75		0,57	N/A	1,30		1,28	N/A	0,27		0,36
1286,54	29,33	Collagen alpha-1(I) chain	N/A	0,36		0,84	N/A	0,57		0,55	N/A	1,37		1,18	N/A	0,77		1,44	N/A	0,52		0,02
1287,59	21,92	Apolipoprotein A-IV	N/A	0,69		0,70	0,01	0,79		0,78	0,00	0,53		0,41	N/A	0,44		0,77	N/A	0,98		0,40
1304,61	22,00	Collagen alpha-1(XXVI) chain	N/A	0,79		0,96	N/A	0,79		0,71	N/A	0,63		0,48	N/A	0,46		0,97	N/A	0,90		0,35
1312,55	29,69	Titin	n.s	0,91		1,10	n.s	0,93		0,76	0,01	0,80		0,73	0,00	0,45		0,75	0,01	0,78		0,64
1321,59	28,38	Collagen alpha-1(I) chain	n.s	1,03		1,26	0,00	0,78		0,72	0,03	0,86		0,64	n.s	0,87		0,93	0,01	0,43		0,45
1336,58	21,56	Collagen alpha-1(IV) chain	N/A	1,66		1,53	N/A	0,85		0,86	N/A	1,26		1,42	N/A	1,04		1,41	N/A	1,88		0,23
1350,57	21,29		N/A	0,51		0,50	N/A	0,43		0,48	N/A	0,21		0,54	N/A	0,87		0,24	N/A	0,26		0,07
1351,63	38,77	Collagen alpha-1(I) chain	N/A	2,93		4,27	N/A	0,86		0,47	N/A	1,44		1,34	N/A	1,12		0,85	N/A	1,16		0,96
1352,79	24,82	Mucin-16	N/A	1,01		0,21	N/A	1,30		3,72	N/A	0,33		0,20	N/A	0,44		0,47	N/A	1,05		0,64
1378,61	28,89	Collagen alpha-1(I) chain	n.s	0,91		1,11	0,04	0,88		0,72	0,01	0,81		0,70	n.s	0,76		0,95	0,02	0,71		0,90

193-peptide marker pattern			Bladder cancer				Prostate cancer				Cholangiocarcinoma				Pancreatic cancer				Renal cell carcinoma			
			Discovery set		Validation set		Discovery set		Validation set		Discovery set		Validation set		Discovery set		Validation set		Discovery set		Validation set	
Mass [Da]	CE migration time [min]	Protein name	adj. P-value	Fold change (case/control)	unadj. P-value	Fold change (case/control)	adj. P-value	Fold change (case/control)	unadj. P-value	Fold change (case/control)	adj. P-value	Fold change (case/control)	unadj. P-value	Fold change (case/control)	adj. P-value	Fold change (case/control)	unadj. P-value	Fold change (case/control)	adj. P-value	Fold change (case/control)	unadj. P-value	Fold change (case/control)
1383,63	38,90	Ras and Rab interactor 3	N/A	4,06		15,42	N/A	0,71		0,33	N/A	0,39		0,42	N/A	0,89		0,74	N/A	0,86		1,44
1390,44	36,82		n.s	1,05		1,46	n.s	0,91		0,59	n.s	0,73		0,91	n.s	0,72		0,90	n.s	0,83		0,38
1396,62	26,63	Collagen alpha-1(III) chain	N/A	0,69		0,94	N/A	0,86		0,58	N/A	0,71		0,87	N/A	0,44		0,47	N/A	0,78		0,76
1407,64	27,55	Collagen alpha-1(I) chain	N/A	0,63		0,92	N/A	0,47		1,23	N/A	0,80		0,65	N/A	0,72		1,33	N/A	1,55		1,35
1408,65	39,07	Collagen alpha-1(I) chain	N/A	1,86		2,53	N/A	0,71		0,58	N/A	0,82		0,55	N/A	1,23		1,08	N/A	2,00		1,28
1422,53	37,70	Ankyrin repeat domain 17	N/A	0,68		1,06	N/A	0,93		0,68	0,00	0,69		0,65	0,00	0,49		1,14	n.s	0,78		1,21
1423,64	37,35	Fibrinogen alpha chain	N/A	2,02		1,28	N/A	1,82		1,12	N/A	1,76		0,96	N/A	0,91		0,63	N/A	0,79		0,13
1425,59	22,34		n.s	1,00		1,03	0,01	0,81		0,77	0,00	0,56		0,54	0,00	0,52		0,71	n.s	0,88		0,58
1462,61	39,50	Collagen alpha-3(V) chain	N/A	1,25		2,80	N/A	1,38		0,99	N/A	1,02		0,72	N/A	1,27		6,43	N/A	0,25		0,09
1485,68	23,77	Collagen alpha-1(I) chain	n.s	1,11		0,94	n.s	0,94		1,01	0,00	0,72		0,76	0,03	0,76		0,97	n.s	1,07		0,63
1495,68	39,45	Collagen alpha-1(I) chain	N/A	1,31		3,05	N/A	0,85		0,46	N/A	1,67		1,54	N/A	1,31		1,20	N/A	3,81		2,45
1505,61	28,85	Nuclear receptor corepressor 1	N/A	0,78		0,61	N/A	0,95		0,86	N/A	0,81		0,82	N/A	0,80		1,70	N/A	0,51		0,30
1524,65	20,03	Collagen alpha-1(XI) chain	n.s	0,92		0,85	n.s	0,87		0,69	0,00	0,47		0,73	0,01	0,59		0,65	n.s	0,76		0,28
1525,55	21,83		N/A	0,59		0,93	N/A	2,03		2,30	N/A	0,71		0,64	N/A	0,39		1,36	N/A	0,99		0,23
1552,5	39,02		N/A	0,68		2,38	N/A	2,29		0,96	N/A	0,16		0,21	N/A	0,36		1,02	N/A	0,66		0,24
1560,57	21,53		N/A	0,55		0,62	N/A	1,41		1,47	N/A	0,63		0,19	N/A	0,53		1,17	N/A	0,18		0,00
1564,72	28,29	Collagen alpha-1(V) chain	N/A	0,47		0,50	N/A	0,56		1,10	N/A	0,61		0,32	N/A	0,86		0,62	N/A	1,64		2,65
1594,76	40,28	Collagen alpha-1(I) chain	n.s	1,09		1,18	n.s	1,19		1,01	n.s	0,77		0,81	n.s	1,02		1,16	n.s	1,51		1,88
1610,88	30,35		N/A	1,36		3,05	N/A	1,48		1,06	N/A	2,16		1,75	N/A	1,60		1,32	N/A	1,21		1,80
1620,7	22,69	Collagen alpha-2(I) chain	N/A	1,54		0,92	N/A	0,73		0,45	N/A	0,27		0,31	N/A	0,53		1,41	N/A	1,04		1,42
1651,79	40,75	Collagen alpha-1(I) chain	n.s	1,10		1,13	0,03	1,18		1,04	n.s	0,96		0,98	n.s	1,12		1,08	0,02	1,96		1,77
1703,83	33,54	Collagen alpha-3(V) chain	N/A	1,54		1,50	N/A	0,91		0,57	N/A	0,30		1,08	N/A	0,31		0,25	N/A	0,39		1,41
1716,65	20,16		N/A	1,30		0,63	N/A	0,95		0,66	N/A	0,64		0,31	N/A	0,62		0,96	N/A	0,63		0,18
1720,69	19,64		N/A	0,84		0,38	N/A	0,75		0,45	N/A	0,72		0,36	N/A	1,35		2,21	N/A	0,43		0,03
1734,66	19,83		N/A	0,94		0,46	N/A	0,98		0,65	N/A	0,54		0,41	N/A	0,64		0,88	N/A	0,40		0,12
1764,68	19,92		N/A	1,06		0,92	N/A	0,82		0,61	N/A	0,71		0,18	N/A	0,69		1,73	N/A	0,45		0,04
1767	24,90		N/A	0,08		0,04	N/A	1,70		10,42	N/A	0,34		0,42	N/A	0,28		1,23	N/A	1,04		1,42
1767,78	19,80	Fibrinogen alpha chain	N/A	0,88		1,21	N/A	1,54		1,01	N/A	1,92		4,23	N/A	1,92		0,53	N/A	2,30		0,77
1817,7	20,25	Stabilin-2	n.s	0,80		1,02	n.s	0,87		0,73	0,01	0,71		0,31	n.s	0,85		1,33	0,04	0,59		0,33
1828,85	21,21	Collagen alpha-1(I) chain	N/A	1,54		1,21	N/A	1,38		1,84	N/A	2,04		1,33	N/A	0,94		1,00	N/A	1,42		0,36
1858,84	24,34	Prostaglandin-H2 D-isomerase	N/A	0,87		1,74	N/A	1,21		0,18	N/A	1,12		5,76	N/A	0,51		0,37	N/A	0,26		1,33
1885,87	21,25	Collagen alpha-1(I) chain	N/A	1,73		1,12	N/A	1,79		4,40	N/A	1,49		1,97	N/A	2,12		1,31	N/A	1,80		0,66
1889,86	33,19	Collagen alpha-2(IV) chain	N/A	1,11		0,89	N/A	3,36		7,91	N/A	0,99		0,93	N/A	1,25		0,82	N/A	3,06		5,57
1892,87	22,13	Collagen alpha-1(I) chain	n.s	1,21		0,99	0,01	1,65		1,33	n.s	1,09		1,38	n.s	0,94		0,61	0,03	1,73		1,00
1893,04	29,05	26S protease regulatory subunit 10B	N/A	0,53		0,90	N/A	0,86		0,82	N/A	0,83		0,76	N/A	0,57		0,19	N/A	0,74		0,49
1899,81	22,44		N/A	0,60		0,23	N/A	1,81		1,34	N/A	0,56		0,38	N/A	0,32		0,97	N/A	0,10		0,05
1911,05	25,23		n.s	0,89		0,88	0,00	0,61		0,79	0,00	0,50		0,57	0,00	0,52		0,63	n.s	0,92		0,84
1915	33,62	Metastasis suppressor protein 1	N/A	1,01		3,00	N/A	2,24		1,08	N/A	2,54		1,48	N/A	4,25		1,53	N/A	3,43		9,03
1933,88	21,64	Collagen alpha-1(I) chain	n.s	1,23		0,79	0,03	1,48		1,53	n.s	1,22		1,61	0,01	1,50		0,97	n.s	1,95		1,03
1945	33,66	Glycogen phosphorylase, brain form	n.s	1,17		1,49	n.s	1,21		0,91	n.s	1,61		0,97	n.s	2,05		1,27	n.s	1,38		2,89
1991,94	22,05	Collagen alpha-1(III) chain	n.s	1,47		1,00	0,03	1,24		0,96	0,02	2,19		2,01	0,01	1,80		0,88	0,01	2,33		2,39
1996,78	20,96		N/A	1,07		0,51	n.s	1,01		0,69	0,03	0,71		0,24	N/A	0,89		1,47	0,01	0,42		0,17
2093,93	33,71	Collagen alpha-2(I) chain	N/A	1,49		1,67	N/A	4,60		4,76	N/A	0,98		0,43	N/A	0,78		0,75	N/A	2,44		5,91
2152,97	32,92		N/A	0,98		3,49	N/A	0,96		1,05	N/A	2,54		1,47	N/A	2,99		0,71	N/A	2,89		4,96
2170,97	27,53	Collagen alpha-1(I) chain	N/A	0,42		0,54	N/A	0,90		0,93	N/A	0,99		0,31	N/A	1,75		0,74	N/A	1,01		1,25
2187,96	39,54	Mucin-12	n.s	0,72		0,83	n.s	0,96		0,97	0,01	0,57		0,61	0,02	0,30		0,55	0,02	0,58		0,37
2199	22,33	Collagen alpha-1(I) chain	N/A	2,49		0,57	N/A	0,06		1,33	N/A	0,82		1,11	N/A	1,93		1,23	N/A	2,75		0,74
2264,94	43,13	Collagen alpha-2(IV) chain	N/A	0,55		1,45	N/A	2,79		5,60	N/A	1,58		0,67	N/A	1,24		0,45	N/A	1,04		0,72
2272,24	23,91	Histone deacetylase 6	N/A	2,98		1,53	N/A	1,01		0,65	N/A	2,05		1,94	0,04	1,93		1,14	0,00	3,95		2,66
2306,04	19,52	Fibrinogen alpha chain	N/A	0,54		2,32	N/A	0,74		1,13	N/A	5,07		7,72	N/A	4,85		1,11	N/A	2,87		2,30
2548	21,36		N/A	0,68		0,49	N/A	1,15		1,08	N/A	0,81		0,27	N/A	1,32		1,19	N/A	0,69		0,58
2565,15	23,60	Collagen alpha-1(XVII) chain	N/A	1,26		1,50	N/A	0,93		1,27	N/A	3,43		3,08	N/A	1,38		0,81	N/A	7,31		6,88
2583,2	28,30	Collagen alpha-1(I) chain	n.s	1,20		0,70	n.s	0,98		1,26	0,01	1,49		1,69	0,02	1,53		1,10	0,01	1,53		2,88
2600,23	35,20	Collagen alpha-1(I) chain	N/A	2,16		1,35	N/A	0,99		1,37	N/A	1,55		0,26	N/A	1,18		0,88	N/A	2,54		2,46
2647,21	23,52	Collagen alpha-1(III) chain	N/A	3,29		1,25	N/A	1,73		1,20	N/A	1,28		2,37	N/A	0,98		0,71	N/A	1,75		0,60
2663,21	23,57	Collagen alpha-1(III) chain	0,02	1,92		1,19	0,02	1,51		0,99	n.s	0,88		1,10	n.s	0,80		0,73	0,04	1,71		1,20
2734,29	27,99		N/A	1,07		2,35	N/A	3,06		3,35	N/A	2,43		1,09	N/A	1,09		0,95	N/A	4,95		3,46
2739,23	28,46	Chloride intracellular channel protein 6	N/A	1,13		0,81	N/A	1,53		1,12	N/A	1,58		2,09	N/A	1,38		0,92	N/A	1,50		3,09
2751,34	29,11		N/A	0,77		0,57	N/A	0,64		0,52	N/A	0,32		0,46	N/A	0,27		0,63	N/A	0,93		0,72
2808,3	24,33		N/A	1,37		0,92	N/A	1,55		2,98	N/A	0,96		0,80	N/A	1,22		0,70	N/A	5,14		5,19
2834,19	22,35		N/A	0,43		0,26	N/A	0,94		0,91	N/A	0,26		0,21	N/A	0,27		0,21	N/A	0,14		0,09
2854,37	34,67	Collagen alpha-1(III) chain	n.s	0,75		1,14	0,00	0,68		0,73	0,00	0,54		0,49	0,00	0,58		0,88	n.s	0,62		1,00
2861,29	48,80	Protein transport protein Sec31A	N/A	0,29		1,24	N/A	0,57		0,88	N/A	1,18		1,18	N/A	0,74		0,46	N/A	0,78		0,13
2887,35	35,57	Collagen alpha-2(V) chain	N/A	1,55		1,08	N/A	1,11		1,32	n.s	1,24		0,98	n.s	1,15		0,44	N/A	2,00		1,53
2903,35	35,64	Collagen alpha-2(I) chain	N/A	1,62		4,36	N/A	2,03		1,36	N/A	1,42		1,22	N/A	1,25		0,60	N/A	2,40		2,59
2946,2	35,07		N/A	2,33		0,61	N/A	2,13		1,34	N/A	1,02		0,71	N/A	1,04		1,03	N/A	0,51		3,81
2959,36	48,32	Collagen alpha-1(III) chain	N/A	0,35		1,37	N/A	0,51		0,59	N/A	0,74		1,92	N/A	0,40		0,72	N/A	0,77		0,25
2973,46	24,38	Collagen alpha-1(I) chain	N/A	2,12		1,13	N/A	1,24		0,89	N/A	1,28		0,88	N/A	1,49		0,84	N/A	2,10		3,33
2989,44	24,47	Collagen alpha-1(I) chain	N/A	1,47		0,60	N/A	1,54		1,08	N/A	1,52		1,34	N/A	1,76		0,98	N/A	3,53		2,44
3017,4	48,54	Collagen alpha-2(V) chain	N/A	0,24		0,76	N/A	0,72		0,52	N/A	0,67		0,93	n.s	0,88		0,52	N/A	0,70		0,52
3023,41	20,91	Collagen alpha-1(III) chain	N/A	2,03		0,46	N/A	1,40		0,77	N/A	0,91		1,81	N/A	1,13		1,00	N/A	2,39		0,85
3025,4	29,89	Collagen alpha-2(I) chain	N/A	2,44		2,09	N/A	1,52		0,99	N/A	1,46		1,24	N/A	1,61		2,29	N/A	1,17		2,04
3031,44	35,84	Collagen alpha-1(I) chain	N/A	2,02		1,11	N/A	1,23		2,00	N/A	1,11		0,65	n.s	1,11		0,49	N/A	2,79		1,60
3039,39	24,60	Collagen alpha-2(IV) chain	N/A	1,09		3,34	N/A	2,65		1,76	N/A	1,29		1,01	N/A	1,05		0,40	N/A	9,98		4,40
3057,39	29,96	Collagen alpha-2(V) chain	N/A	1,27		0,59	N/A	4,34		2,75	N/A	1,33		2,70	N/A	0,55		1,20	N/A	1,59		0,84
3092,44	36,22	Collagen alpha-2(I) chain	N/A	1,57		0,98	N/A	1,60		1,22	N/A	1,14		0,66	n.s	0,77		0,76	N/A	1,38		1,27
3158,42	29,80	Collagen alpha-1(XI) chain	n.s	1,81		0,98	N/A	1,41		1,24	n.s	1,35		2,97	n.s	1,26		1,03	N/A	1,51		1,09
3184,36	24,71	Collagen alpha-1(V) chain	N/A	4,07		3,94	N/A	2,48		1,35	N/A	1,06		3,14	N/A	2,61		1,35	N/A	2,18		11,18
3194,43	30,28	Collagen alpha-1(III) chain	N/A	1,64		3,28	N/A	4,67		6,60	N/A	1,59		4,59	N/A	1,02		1,09	N/A	4,04		4,34
3261,53	22,19		N/A	1,58		0,36	N/A	1,44		0,88	N/A	0,60		1,14	N/A	0,95		0,57	N/A	5,69		1,23
3265,44	35,90	Collagen alpha-1(XI) chain	N/A	1,00		0,86	N/A	2,45		2,33	N/A	0,77		0,84	n.s	0,75		0,96	N/A	1,99		0,96
3288,52	36,49	Receptor-type tyrosine-protein phosphatase U	N/A	1,86		2,20	N/A	2,30		3,58	N/A	1,33		4,11	N/A	1,21		0,40	N/A	1,75		0,27
3290,54	23,89	Collagen alpha-2(V) chain	N/A	0,50		0,67	N/A	1,04		0,60	N/A	0,63		0,65	N/A	0,34		0,56	N/A	0,80		0,76
3298,46	35,95	Collagen alpha-1(I) chain	N/A	6,95		2,88	N/A	0,67		2,04	N/A	1,80		2,84	N/A	0,88		1,73	N/A	2,02		5,60
3313,54	25,62	Collagen alpha-2(I) chain	N/A	2,04		1,52	N/A	1,75		1,43	N/A	1,66		1,80	N/A	1,52		1,13	N/A	2,43		22,73

193-peptide marker pattern			Bladder cancer				Prostate cancer				Cholangiocarcinoma				Pancreatic cancer				Renal cell carcinoma			
Mass [Da]	CE migration time [min]	Protein name	Discovery set		Validation set		Discovery set		Validation set		Discovery set		Validation set		Discovery set		Validation set		Discovery set		Validation set	
			adj. P-value	Fold change (case/control)	unadj. P-value	Fold change (case/control)	adj. P-value	Fold change (case/control)	unadj. P-value	Fold change (case/control)	adj. P-value	Fold change (case/control)	unadj. P-value	Fold change (case/control)	adj. P-value	Fold change (case/control)	unadj. P-value	Fold change (case/control)	adj. P-value	Fold change (case/control)	unadj. P-value	Fold change (case/control)
3318,52	30,97	Collagen alpha-1(I) chain	N/A	1,88		2,43	N/A	2,26		2,22	N/A	2,05		0,97	N/A	0,79		1,27	N/A	1,27		3,28
3356,52	25,46	Collagen alpha-1(I) chain	N/A	2,07		2,89	N/A	2,33		8,72	N/A	1,26		2,82	N/A	1,12		0,85	N/A	5,73		4,75
3385,59	25,54		N/A	1,59		0,92	N/A	0,94		1,01	N/A	0,76		0,72	N/A	1,12		1,23	N/A	1,80		2,29
3401,61	25,47	Collagen alpha-1(V) chain	0,02	1,61		0,81	n.s	1,04		1,22	n.s	0,92		1,35	n.s	0,91		1,14	0,00	2,04		1,58
3439,59	38,90	Collagen alpha-1(XII) chain	N/A	1,61		6,36	N/A	0,72		0,73	N/A	0,43		2,88	N/A	1,07		0,47	N/A	1,58		2,60
3489,58	31,42		N/A	1,35		0,97	N/A	2,28		2,87	N/A	0,83		1,73	N/A	0,63		1,02	N/A	1,01		0,98
3546,67	26,15		N/A	3,08		2,40	N/A	4,54		1,52	N/A	3,11		1,96	N/A	0,26		0,39	N/A	0,92		2,36
3582,75	19,46		N/A	2,66		2,53	N/A	0,76		0,60	N/A	1,67		2,10	N/A	0,89		1,05	N/A	1,72		0,49
3583,63	26,32	Collagen alpha-1(III) chain	N/A	2,01		1,12	N/A	2,13		1,60	N/A	0,64		1,41	N/A	0,86		0,81	N/A	5,01		8,35
3593,47	20,19	Collagen alpha-1(XI) chain	N/A	1,15		1,25	N/A	1,76		1,65	N/A	0,79		1,05	N/A	1,99		0,76	N/A	0,64		0,48
3618,67	31,67	Collagen alpha-1(I) chain	N/A	1,42		1,40	N/A	1,55		1,60	N/A	1,19		1,18	N/A	1,42		0,94	N/A	1,07		1,92
3681,71	31,87		N/A	2,53		1,46	N/A	1,63		106,84	N/A	1,15		2,45	N/A	1,04		1,12	N/A	1,26		2,24
3710,76	19,57	Collagen alpha-1(XVI) chain	N/A	6,56		1,63	N/A	1,23		0,70	N/A	2,57		2,71	N/A	0,73		0,34	N/A	2,37		1,18
3822,11	24,81		N/A	0,74		0,57	N/A	0,42		1,10	N/A	0,33		0,26	N/A	0,53		0,78	N/A	0,54		0,56
3943,83	33,54	Collagen alpha-1(III) chain	N/A	1,46		1,01	N/A	1,38		1,37	N/A	0,92		0,72	n.s	0,71		1,01	n.s	1,37		1,53
4043,65	20,16		N/A	4,62		0,94	N/A	0,91		1,12	N/A	1,08		0,21	N/A	0,79		1,51	N/A	1,93		0,23
4059,65	20,38		N/A	15,25		1,28	N/A	3,39		6,36	N/A	1,21		0,48	N/A	0,93		1,69	N/A	1,96		1,46
4078,81	33,06	Collagen alpha-3(IV) chain	N/A	1,78		1,84	N/A	2,59		1,35	N/A	2,40		1,70	N/A	0,54		0,87	N/A	3,09		1,94
4185,91	33,60		N/A	2,62		2,12	N/A	1,32		2,09	N/A	1,03		0,73	N/A	0,70		0,77	N/A	2,08		4,51
4196,78	20,85		N/A	0,61		0,27	N/A	1,21		0,74	N/A	0,42		0,39	N/A	1,11		4,79	N/A	0,71		0,45
4368,87	20,21	Collagen alpha-1(XI) chain	N/A	13,14		1,25	N/A	2,49		4,76	N/A	1,07		0,56	N/A	1,63		1,46	N/A	2,51		0,71
4404,87	20,59		N/A	0,88		0,42	n.s	0,85		0,88	N/A	0,69		0,36	N/A	0,86		2,12	N/A	0,71		0,53
4654,14	25,78	Collagen alpha-1(III) chain	N/A	1,06		1,09	N/A	0,77		0,82	N/A	2,16		1,20	N/A	1,01		0,96	N/A	1,78		2,14
4817,17	23,74	Collagen alpha-1(II) chain	N/A	5,32		5,18	N/A	1,46		23,21	N/A	1,76		1,33	N/A	1,34		0,49	N/A	7,65		33,99
4833,16	23,83	Collagen alpha-1(I) chain	N/A	2,47		6,54	N/A	2,74		0,79	N/A	1,01		2,43	N/A	1,06		0,60	N/A	9,27		50,19
4960,48	20,57	Collagen alpha-1(II) chain	N/A	1,16		1,63	N/A	1,53		0,56	N/A	9,51		0,21	N/A	0,09		0,29	N/A	9,07		0,56
5510,35	26,99		n.s	0,66		0,74	N/A	0,93		1,26	0,00	0,42		0,37	0,03	0,43		0,55	N/A	1,19		1,09
6211,71	20,26		N/A	0,99		0,36	N/A	0,67		0,80	N/A	0,08		0,56	N/A	0,31		0,08	N/A	1,64		0,34
6236,85	21,06		0,02	3,78		2,28	N/A	1,47		1,34	n.s	9,83		0,97	n.s	0,94		0,25	0,01	2,51		4,20
9882,83	20,82		N/A	4,60		1,06	N/A	3,27		9,10	N/A	0,62		0,69	N/A	0,28		0,07	N/A	2,67		1,11
10999,63	21,33		N/A	1,40		0,07	N/A	0,66		1,01	N/A	0,66		1,24	N/A	1,22		1,43	N/A	0,93		0,36

Abbreviations: CE-MS, Capillary Electrophoresis coupled to Mass Spectrometry; Da, Dalton; min, minutes; N/A

Supplementary Table S3. **Spreadsheet 1**. Distribution of the bladder cancer cohort.

Bca cases

	Discovery Phase	Validation Phase	P
Patients, n(%)	349 (64%)	138 (25%)	
Female/male, n/n	65/283	28/110	0.07
Age, mean/range	67/28-95	69/27-87	0.05
Stage, n(%)			
pTa	151	62	
pTis	16	6	
pT1	71	24	
pT2	72	30	
pT3	29	14	
pT4	9	2	
No gender data available, n	1		
No staging data available, n	1		

Urological controls

	Discovery Phase	Validation Phase	P
Patients, n(%)	35 (7%)	23 (4%)	
Female/male, n/n	4/31	8/15	0.05
Age, mean/range	60/43-88	65/33-93	0.31

Abbreviations: Bca, Bladder cancer; P, p-value

Supplementary Table S3. **Spreadsheet 2**. Distribution of the prostate cancer cohort.

Prostate cancer cases

	Discovery Phase	Validation Phase	P
Patients, n (%)	138 (36%)	68 (18%)	
Age, mean/range	68 (41-87)	68 (45-86)	0.93
Stage, n	32	3	
pT1	1		
pT2	9	1	
pT3	15	2	
pT4	7		
No staging data available, n	106	65	
Gleason score			
6	12	5	
7	28	16	
8-10	12	4	
No Gleason score data available, n	86	43	
PSA level (ng/ml)	38.8 (1.61-987)	12.1 (1.2-136)	
No PSA level data available, n	13	5	

BPH controls

	Discovery Phase	Validation Phase	P
Patients, n (%)	130 (33%)	49 (13%)	
Age, mean/range	66 (51-87)	66 (51-83)	0.61
PSA level (ng/ml)	13.1 (0.6-72.5)	17.8 (3.05-384)	
No PSA level data available, n	13	6	

Abbreviations: BPH, Benign Prostatic Hyperplasia; PSA, prostate specific antigen; P, p-value

Supplementary Table S3. **Spreadsheet 3**. Distribution of the pancreatic cancer cohort.

Pancreatic cancer cases

	Discovery Phase	Validation Phase	P
Patients, n(%)	61 (%)	27 (25%)	
Female/male, n/n	21/40	13/4	0.24
Age, mean/range	67/28-95	69/27-87	0.51
CEA level	33001 (1-575127.6)	11515.5 (7-120931)	
No CEA level data available, n	17	8	
CA19-9 level	84.8 (1-1051)	7 (0-36)	
No CA19-9 level data available, n	27	9	

Chronic pancreatitis controls

	Discovery Phase	Validation Phase	P
Patients, n(%)	62 (7%)	30 (4%)	
Female/male, n/n	10/52	3/27	0.54
Age, mean/range	60/43-88	65/33-93	0.87
CEA level	5.2 (1-30)	8.5 (1-112)	
No CEA level data available, n	18	8	
CA19-9 level	26.5 (1-598.7)	51.8 (0.6-363)	
No CA19-9 level data available, n	17	8	

Abbreviations: CEA, carcinoembryonic antigen; CA19-9, carbohydrate antigen 19-9; P, p-value

Supplementary Table S3. **Spreadsheet 4**. Distribution of the renal cell carcinoma cohort.

RCC cases

	Discovery Phase	Validation Phase	P
Patients, n(%)	46 (26%)	24 (14%)	
Female/male, n/n	16/30	9/15	1
Age, mean/range	62/41-81	61/33-79	0.87
Stage, n (%)			
pT1	18	3	
pT2	8		
pT3	12	1	
No staging data available, n	8	20	

RCC control

	Discovery Phase	Validation Phase	P
Patients, n(%)	80 (46%)	25 (14%)	
Female/male, n/n	22/35	11/14	0.81
Age, mean/range	58/23-84	59/28-80	0.94
No gender data available, n	23		

Abbreviations: RCC, Renal Cell Carcinoma; P, p-value

Supplementary Table S3. **Spreadsheet 5**. Distribution of the cholangiocarcinoma cohort.

CC cases

	Discovery Phase	Validation Phase	P
Patients, n(%)	82 (%)	36 (%)	
Female/male, n/n	32/49	11/25	0.41
No gender data available, n	1		
Age, mean/range	61/22-81	58/30-86	0.49

Chronic pancreatitis controls

	Discovery Phase	Validation Phase	P
Patients, n(%)	83 (7%)	39 (4%)	
Female/male, n/n	29/52	15/24	0.84
No gender data available, n	2		
Age, mean/range	48/20-83	50/22-85	0.45

Abbreviations: CC, Cholangiocarcinoma; P, p-value

Supplementary Table S3. **Spreadsheet 6**. Distribution of the control set of health individuals.

Healthy Individuals

	Discovery Phase	Validation Phase	P
Patients, n(%)	167 (51%)	89 (27%)	
Female/male, n/n	90/77	48/41	1.0
Age, mean (range)	60 (19-86)	60 (22-86)	0.75

Individuals with smoking history

	Discovery Phase	Validation Phase	P
Patients, n(%)	51(15%)	24 (7%)	
Female/male, n/n	28/23	13/11	1.0
Age, mean (range)	43 (19-72)	46 (20-72)	0.55

Supplementary Table S3. **Spreadsheet 7**. Distribution of the control set of patients with inflammatory diseases.

Inflammatory diseases			
	Discovery Phase	Validation Phase	P
Patients, n(%)	136	63	
Female/male, n/n	79/57	40/23	0.54
Age, mean (range)	46 (19-88)	47 (19-78)	0.93

Supplementary Table S3. **Spreadsheet 8**. Distribution of the independent control set of patients with inflammatory diseases.

Independent specificity phase

	HV
Patients, n(%)	58 (63%)
Female/male, n/n	16/3
No gender data available, n	39
Age, mean (range)	30 (19-47)
	**SLE**
Patients, n(%)	34 (37%)
Female/male, n/n	16/2
No gender data available, n	16
Age, mean (range)	42 (27-63)

Abbreviations: HV, Hypersensitivity vasculitis; SLE, Systemic Lupus erytemathosus

Supplementary Table S3. **Spreadsheet 9**. Distribution of the additional set of patients with other tumor types.

Additional Validation Phase

	AML
Patients, n(%)	27 (63%)
Female/male, n/n	13/14
Age, mean (range)	50 (17-70)

	Other tumors
Patients, n(%)	13 (37%)
Female/male, n/n	7/6
Age, mean (range)	52 (33-74)

Abbreviations: AML, Acute Myeloid Leukemia

Supplementary Table S4. **Spreadsheet 1**. Clinical and demographical data of the bladder cancer cohort (n=545).

CE-MS Analysis ID	Diagnosis	Stage	Age	Gender	Analytical Phase
20877	Urological Control		51	Female	Training/Discovery Phase
80120	Urological Control		52	Female	Training/Discovery Phase
18236	Urological Control		56	Female	Training/Discovery Phase
85255	Urological Control		77	Female	Training/Discovery Phase
78461	Urological Control		43	Male	Training/Discovery Phase
80338	Urological Control		46	Male	Training/Discovery Phase
21479	Urological Control		48	Male	Training/Discovery Phase
20977	Urological Control		49	Male	Training/Discovery Phase
82784	Urological Control		50	Male	Training/Discovery Phase
19851	Urological Control		51	Male	Training/Discovery Phase
21503	Urological Control		52	Male	Training/Discovery Phase
21530	Urological Control		52	Male	Training/Discovery Phase
19875	Urological Control		54	Male	Training/Discovery Phase
21502	Urological Control		54	Male	Training/Discovery Phase
81353	Urological Control		54	Male	Training/Discovery Phase
19864	Urological Control		57	Male	Training/Discovery Phase
19841	Urological Control		58	Male	Training/Discovery Phase
27154	Urological Control		59	Male	Training/Discovery Phase
27363	Urological Control		59	Male	Training/Discovery Phase
19866	Urological Control		61	Male	Training/Discovery Phase
19916	Urological Control		61	Male	Training/Discovery Phase
21736	Urological Control		61	Male	Training/Discovery Phase
27188	Urological Control		61	Male	Training/Discovery Phase
81677	Urological Control		61	Male	Training/Discovery Phase
19897	Urological Control		62	Male	Training/Discovery Phase
80730	Urological Control		64	Male	Training/Discovery Phase
21739	Urological Control		68	Male	Training/Discovery Phase
82625	Urological Control		68	Male	Training/Discovery Phase
19854	Urological Control		69	Male	Training/Discovery Phase
82606	Urological Control		71	Male	Training/Discovery Phase
85367	Urological Control		71	Male	Training/Discovery Phase
19850	Urological Control		73	Male	Training/Discovery Phase
19842	Urological Control		74	Male	Training/Discovery Phase
19853	Urological Control		78	Male	Training/Discovery Phase
20881	Urological Control		88	Male	Training/Discovery Phase
85309	Bca case	pT2	28	Female	Training/Discovery Phase
27713	Bca case	pTa	39	Female	Training/Discovery Phase
85736	Bca case	pTa	43	Female	Training/Discovery Phase
87823	Bca case	pT3	47	Female	Training/Discovery Phase
21480	Bca case	pTa	47	Female	Training/Discovery Phase
22109	Bca case	pTa	47	Female	Training/Discovery Phase
81569	Bca case	pTis	48	Female	Training/Discovery Phase
82118	Bca case	pT3	51	Female	Training/Discovery Phase
26639	Bca case	pTa	57	Female	Training/Discovery Phase
88142	Bca case	pTa	57	Female	Training/Discovery Phase
27080	Bca case	pTis	59	Female	Training/Discovery Phase

CE-MS Analysis ID	Diagnosis	Stage	Age	Gender	Analytical Phase
27169	Bca case	pT4	60	Female	Training/Discovery Phase
88118	Bca case	pTa	60	Female	Training/Discovery Phase
88119	Bca case	pTa	60	Female	Training/Discovery Phase
88100	Bca case	pT1	61	Female	Training/Discovery Phase
87744	Bca case	pT3	61	Female	Training/Discovery Phase
18755	Bca case	pTa	61	Female	Training/Discovery Phase
85244	Bca case	pTa	61	Female	Training/Discovery Phase
87738	Bca case	pT2	62	Female	Training/Discovery Phase
19845	Bca case	pTa	62	Female	Training/Discovery Phase
27694	Bca case	pTa	63	Female	Training/Discovery Phase
88110	Bca case	pTa	63	Female	Training/Discovery Phase
32555	Bca case	pT2	64	Female	Training/Discovery Phase
87893	Bca case	pT2	64	Female	Training/Discovery Phase
87728	Bca case	pT4	64	Female	Training/Discovery Phase
19871	Bca case	pTa	64	Female	Training/Discovery Phase
22024	Bca case	pT1	65	Female	Training/Discovery Phase
27691	Bca case	pTa	66	Female	Training/Discovery Phase
18852	Bca case	pT1	68	Female	Training/Discovery Phase
87854	Bca case	pT3	68	Female	Training/Discovery Phase
82330	Bca case	pTa	68	Female	Training/Discovery Phase
84989	Bca case	pTa	68	Female	Training/Discovery Phase
87821	Bca case	pT3	69	Female	Training/Discovery Phase
87216	Bca case	pTa	69	Female	Training/Discovery Phase
88128	Bca case	pTa	69	Female	Training/Discovery Phase
27182	Bca case	pT2	70	Female	Training/Discovery Phase
27083	Bca case	pT3	71	Female	Training/Discovery Phase
86607	Bca case	pT1	72	Female	Training/Discovery Phase
88046	Bca case	pT3	74	Female	Training/Discovery Phase
27088	Bca case	pTis	74	Female	Training/Discovery Phase
82099	Bca case	pT2	75	Female	Training/Discovery Phase
84957	Bca case	pT1	76	Female	Training/Discovery Phase
86373	Bca case	pT2	76	Female	Training/Discovery Phase
87736	Bca case	pT3	76	Female	Training/Discovery Phase
27692	Bca case	pTa	76	Female	Training/Discovery Phase
86209	Bca case	pTa	76	Female	Training/Discovery Phase
88113	Bca case	pTa	76	Female	Training/Discovery Phase
88145	Bca case	pTa	76	Female	Training/Discovery Phase
27077	Bca case	pT3	77	Female	Training/Discovery Phase
86712	Bca case	pTa	77	Female	Training/Discovery Phase
80735	Bca case	pTa	78	Female	Training/Discovery Phase
88127	Bca case	pT1	79	Female	Training/Discovery Phase
88146	Bca case	pT1	80	Female	Training/Discovery Phase
27551	Bca case	pT1	81	Female	Training/Discovery Phase
86573	Bca case	pT1	81	Female	Training/Discovery Phase
85025	Bca case	pT2	81	Female	Training/Discovery Phase
87743	Bca case	pT3	81	Female	Training/Discovery Phase
84966	Bca case	pTa	81	Female	Training/Discovery Phase
86210	Bca case	pT1	82	Female	Training/Discovery Phase
27068	Bca case	pT3	82	Female	Training/Discovery Phase

CE-MS Analysis ID	Diagnosis	Stage	Age	Gender	Analytical Phase
22025	Bca case	pT1	83	Female	Training/Discovery Phase
85083	Bca case	pT1	83	Female	Training/Discovery Phase
80339	Bca case	pT2	85	Female	Training/Discovery Phase
82121	Bca case	pT2	86	Female	Training/Discovery Phase
85154	Bca case	pT2	86	Female	Training/Discovery Phase
85233	Bca case	pTa	35	Male	Training/Discovery Phase
27069	Bca case	pT3	38	Male	Training/Discovery Phase
18363	Bca case	pT2	40	Male	Training/Discovery Phase
87730	Bca case	pT2	40	Male	Training/Discovery Phase
85304	Bca case	pTa	40	Male	Training/Discovery Phase
85573	Bca case	pTa	40	Male	Training/Discovery Phase
87857	Bca case	pT2	41	Male	Training/Discovery Phase
27190	Bca case	pTa	41	Male	Training/Discovery Phase
87752	Bca case	pTis	41	Male	Training/Discovery Phase
88045	Bca case	pTis	41	Male	Training/Discovery Phase
85012	Bca case	pT1	42	Male	Training/Discovery Phase
87774	Bca case	pT2	43	Male	Training/Discovery Phase
85194	Bca case	pT1	46	Male	Training/Discovery Phase
27364	Bca case	pTa	46	Male	Training/Discovery Phase
88114	Bca case	pTa	46	Male	Training/Discovery Phase
88104	Bca case	pTis	46	Male	Training/Discovery Phase
85235	Bca case	pT1	47	Male	Training/Discovery Phase
81348	Bca case	pTa	47	Male	Training/Discovery Phase
82340	Bca case	pTa	47	Male	Training/Discovery Phase
85813	Bca case	pTa	47	Male	Training/Discovery Phase
86611	Bca case	pTa	47	Male	Training/Discovery Phase
69658	Bca case	pT4	48	Male	Training/Discovery Phase
22020	Bca case	pT3	49	Male	Training/Discovery Phase
87860	Bca case	pT3	49	Male	Training/Discovery Phase
21961	Bca case	pT4	49	Male	Training/Discovery Phase
87780	Bca case	pT2	50	Male	Training/Discovery Phase
84970	Bca case	pTa	50	Male	Training/Discovery Phase
85989	Bca case	pTa	50	Male	Training/Discovery Phase
86241	Bca case	pTa	50	Male	Training/Discovery Phase
87775	Bca case	pT3	51	Male	Training/Discovery Phase
86203	Bca case	pTa	51	Male	Training/Discovery Phase
87885	Bca case	pT2	52	Male	Training/Discovery Phase
88136	Bca case	pT2	52	Male	Training/Discovery Phase
87827	Bca case	pT3	52	Male	Training/Discovery Phase
20540	Bca case	pTa	52	Male	Training/Discovery Phase
27963	Bca case	pTa	52	Male	Training/Discovery Phase
86804	Bca case	pTa	52	Male	Training/Discovery Phase
86805	Bca case	pTa	52	Male	Training/Discovery Phase
85838	Bca case	pT1	53	Male	Training/Discovery Phase
86422	Bca case	pT2	53	Male	Training/Discovery Phase
20733	Bca case	pTa	53	Male	Training/Discovery Phase
27965	Bca case	pTa	53	Male	Training/Discovery Phase
86806	Bca case	pTa	53	Male	Training/Discovery Phase

CE-MS Analysis ID	Diagnosis	Stage	Age	Gender	Analytical Phase
87727	Bca case	pT2	54	Male	Training/Discovery Phase
87886	Bca case	pT3	54	Male	Training/Discovery Phase
85198	Bca case	pTa	54	Male	Training/Discovery Phase
86706	Bca case	pTa	54	Male	Training/Discovery Phase
85234	Bca case	pT1	55	Male	Training/Discovery Phase
88102	Bca case	pT1	55	Male	Training/Discovery Phase
18237	Bca case	pT2	55	Male	Training/Discovery Phase
27067	Bca case	pT2	55	Male	Training/Discovery Phase
87749	Bca case	pT2	55	Male	Training/Discovery Phase
19961	Bca case	pTa	55	Male	Training/Discovery Phase
80963	Bca case	pTa	55	Male	Training/Discovery Phase
86589	Bca case	pT1	56	Male	Training/Discovery Phase
85918	Bca case	pTa	56	Male	Training/Discovery Phase
87856	Bca case	pTis	56	Male	Training/Discovery Phase
88144	Bca case	pTis	56	Male	Training/Discovery Phase
86707	Bca case	pT2	57	Male	Training/Discovery Phase
87859	Bca case	pT2	57	Male	Training/Discovery Phase
78478	Bca case	pTa	57	Male	Training/Discovery Phase
81038	Bca case	pTa	57	Male	Training/Discovery Phase
81041	Bca case	pTa	57	Male	Training/Discovery Phase
82106	Bca case	pTa	57	Male	Training/Discovery Phase
85820	Bca case	pTa	57	Male	Training/Discovery Phase
85943	Bca case	pTa	57	Male	Training/Discovery Phase
20966	Bca case	pTa	58	Male	Training/Discovery Phase
85200	Bca case	pTa	58	Male	Training/Discovery Phase
86709	Bca case	pTa	58	Male	Training/Discovery Phase
27356	Bca case	pT1	59	Male	Training/Discovery Phase
27149	Bca case	pT3	59	Male	Training/Discovery Phase
28580	Bca case	pTa	59	Male	Training/Discovery Phase
84963	Bca case	pTa	59	Male	Training/Discovery Phase
85944	Bca case	pT1	60	Male	Training/Discovery Phase
86037	Bca case	pT1	60	Male	Training/Discovery Phase
86704	Bca case	pT1	60	Male	Training/Discovery Phase
27086	Bca case	pT3	60	Male	Training/Discovery Phase
18746	Bca case	pTa	60	Male	Training/Discovery Phase
19868	Bca case	pTa	60	Male	Training/Discovery Phase
27964	Bca case	pTa	60	Male	Training/Discovery Phase
80662	Bca case	pTa	60	Male	Training/Discovery Phase
80700	Bca case	pTa	60	Male	Training/Discovery Phase
85027	Bca case	pTa	60	Male	Training/Discovery Phase
88129	Bca case	pTa	60	Male	Training/Discovery Phase
80320	Bca case	pT2	61	Male	Training/Discovery Phase
87149	Bca case	pT2	61	Male	Training/Discovery Phase
87746	Bca case	pT2	61	Male	Training/Discovery Phase
88050	Bca case	pT4	61	Male	Training/Discovery Phase
80119	Bca case	pTa	61	Male	Training/Discovery Phase
88101	Bca case	pTa	61	Male	Training/Discovery Phase
19869	Bca case	pT3	62	Male	Training/Discovery Phase
78448	Bca case	pTa	62	Male	Training/Discovery Phase

CE-MS Analysis ID	Diagnosis	Stage	Age	Gender	Analytical Phase
78465	Bca case	pTa	62	Male	Training/Discovery Phase
78528	Bca case	pTa	62	Male	Training/Discovery Phase
87819	Bca case	pT2	63	Male	Training/Discovery Phase
85993	Bca case	pT4	63	Male	Training/Discovery Phase
20690	Bca case	pTa	63	Male	Training/Discovery Phase
87316	Bca case	pTa	63	Male	Training/Discovery Phase
88112	Bca case	pTa	63	Male	Training/Discovery Phase
88105	Bca case	pTis	63	Male	Training/Discovery Phase
88130	Bca case	pTis	63	Male	Training/Discovery Phase
86375	Bca case	pT1	64	Male	Training/Discovery Phase
87858	Bca case	pT2	64	Male	Training/Discovery Phase
88132	Bca case	pT2	64	Male	Training/Discovery Phase
84973	Bca case	pTa	64	Male	Training/Discovery Phase
85013	Bca case	pTa	64	Male	Training/Discovery Phase
85315	Bca case	pTa	64	Male	Training/Discovery Phase
85815	Bca case	pTa	64	Male	Training/Discovery Phase
87862	Bca case	pTis	64	Male	Training/Discovery Phase
82795	Bca case	pT1	65	Male	Training/Discovery Phase
19867	Bca case	pT2	65	Male	Training/Discovery Phase
27179	Bca case	pT2	65	Male	Training/Discovery Phase
27207	Bca case	pT2	65	Male	Training/Discovery Phase
82516	Bca case	pT2	65	Male	Training/Discovery Phase
82599	Bca case	pT2	65	Male	Training/Discovery Phase
27072	Bca case	pT4	65	Male	Training/Discovery Phase
82562	Bca case	pTa	65	Male	Training/Discovery Phase
85078	Bca case	pTa	65	Male	Training/Discovery Phase
86429	Bca case	pTa	65	Male	Training/Discovery Phase
82724	Bca case		65	Male	Training/Discovery Phase
27645	Bca case	pT1	66	Male	Training/Discovery Phase
85942	Bca case	pT1	66	Male	Training/Discovery Phase
86717	Bca case	pT1	66	Male	Training/Discovery Phase
82556	Bca case	pT2	66	Male	Training/Discovery Phase
21842	Bca case	pTa	66	Male	Training/Discovery Phase
82602	Bca case	pTa	66	Male	Training/Discovery Phase
82718	Bca case	pTa	66	Male	Training/Discovery Phase
84960	Bca case	pTa	66	Male	Training/Discovery Phase
88116	Bca case	pTa	66	Male	Training/Discovery Phase
27948	Bca case	pT1	67	Male	Training/Discovery Phase
27954	Bca case	pT1	67	Male	Training/Discovery Phase
27957	Bca case	pT1	67	Male	Training/Discovery Phase
82367	Bca case	pT1	67	Male	Training/Discovery Phase
84987	Bca case	pT1	67	Male	Training/Discovery Phase
88106	Bca case	pT1	67	Male	Training/Discovery Phase
88111	Bca case	pT1	67	Male	Training/Discovery Phase
88139	Bca case	pT1	67	Male	Training/Discovery Phase
23543	Bca case	pT3	67	Male	Training/Discovery Phase
82318	Bca case	pTa	67	Male	Training/Discovery Phase
85020	Bca case	pTa	67	Male	Training/Discovery Phase
88107	Bca case	pTa	67	Male	Training/Discovery Phase

CE-MS Analysis ID	Diagnosis	Stage	Age	Gender	Analytical Phase
88117	Bca case	pTa	67	Male	Training/Discovery Phase
88134	Bca case	pTa	67	Male	Training/Discovery Phase
86812	Bca case	pTis	67	Male	Training/Discovery Phase
19900	Bca case	pTa	68	Male	Training/Discovery Phase
28015	Bca case	pTa	68	Male	Training/Discovery Phase
85023	Bca case	pTis	68	Male	Training/Discovery Phase
27966	Bca case	pT1	69	Male	Training/Discovery Phase
84954	Bca case	pT1	69	Male	Training/Discovery Phase
85937	Bca case	pT1	69	Male	Training/Discovery Phase
85811	Bca case	pT2	69	Male	Training/Discovery Phase
87729	Bca case	pT3	69	Male	Training/Discovery Phase
87735	Bca case	pT3	69	Male	Training/Discovery Phase
87745	Bca case	pT3	69	Male	Training/Discovery Phase
81694	Bca case	pTa	69	Male	Training/Discovery Phase
87114	Bca case	pTa	69	Male	Training/Discovery Phase
22016	Bca case	pT1	70	Male	Training/Discovery Phase
27534	Bca case	pT1	70	Male	Training/Discovery Phase
27712	Bca case	pT1	70	Male	Training/Discovery Phase
87281	Bca case	pT1	70	Male	Training/Discovery Phase
69848	Bca case	pT2	70	Male	Training/Discovery Phase
87737	Bca case	pT3	70	Male	Training/Discovery Phase
26566	Bca case	pTa	70	Male	Training/Discovery Phase
86710	Bca case	pT1	71	Male	Training/Discovery Phase
19852	Bca case	pT2	71	Male	Training/Discovery Phase
78373	Bca case	pT2	71	Male	Training/Discovery Phase
81330	Bca case	pT2	71	Male	Training/Discovery Phase
81349	Bca case	pT2	71	Male	Training/Discovery Phase
85844	Bca case	pT2	71	Male	Training/Discovery Phase
87892	Bca case	pT2	71	Male	Training/Discovery Phase
87889	Bca case	pT3	71	Male	Training/Discovery Phase
85848	Bca case	pTa	71	Male	Training/Discovery Phase
81601	Bca case	pTis	71	Male	Training/Discovery Phase
87740	Bca case	pT2	72	Male	Training/Discovery Phase
27553	Bca case	pTa	72	Male	Training/Discovery Phase
80955	Bca case	pTa	72	Male	Training/Discovery Phase
81355	Bca case	pTa	72	Male	Training/Discovery Phase
84965	Bca case	pTa	72	Male	Training/Discovery Phase
85816	Bca case	pTa	72	Male	Training/Discovery Phase
87116	Bca case	pTa	72	Male	Training/Discovery Phase
85817	Bca case	pT1	73	Male	Training/Discovery Phase
85931	Bca case	pT1	73	Male	Training/Discovery Phase
87731	Bca case	pT2	73	Male	Training/Discovery Phase
87742	Bca case	pT4	73	Male	Training/Discovery Phase
85991	Bca case	pTa	73	Male	Training/Discovery Phase
86605	Bca case	pTa	73	Male	Training/Discovery Phase
81322	Bca case	pT1	74	Male	Training/Discovery Phase
21985	Bca case	pT2	74	Male	Training/Discovery Phase
85161	Bca case	pT2	74	Male	Training/Discovery Phase
78111	Bca case	pTa	74	Male	Training/Discovery Phase

CE-MS Analysis ID	Diagnosis	Stage	Age	Gender	Analytical Phase
80810	Bca case	pTa	74	Male	Training/Discovery Phase
81593	Bca case	pTa	74	Male	Training/Discovery Phase
82305	Bca case	pTa	74	Male	Training/Discovery Phase
85941	Bca case	pTa	74	Male	Training/Discovery Phase
86714	Bca case	pTa	74	Male	Training/Discovery Phase
87865	Bca case	pT1	75	Male	Training/Discovery Phase
27070	Bca case	pT2	75	Male	Training/Discovery Phase
27209	Bca case	pT2	75	Male	Training/Discovery Phase
81046	Bca case	pT2	75	Male	Training/Discovery Phase
19843	Bca case	pT3	75	Male	Training/Discovery Phase
82109	Bca case	pTa	75	Male	Training/Discovery Phase
84981	Bca case	pTa	75	Male	Training/Discovery Phase
86713	Bca case	pTa	75	Male	Training/Discovery Phase
88108	Bca case	pTa	75	Male	Training/Discovery Phase
84964	Bca case	pT1	76	Male	Training/Discovery Phase
86718	Bca case	pT1	76	Male	Training/Discovery Phase
87747	Bca case	pT1	76	Male	Training/Discovery Phase
87890	Bca case	pT1	76	Male	Training/Discovery Phase
88150	Bca case	pT1	76	Male	Training/Discovery Phase
18383	Bca case	pT2	76	Male	Training/Discovery Phase
27064	Bca case	pT2	76	Male	Training/Discovery Phase
84977	Bca case	pT2	76	Male	Training/Discovery Phase
87118	Bca case	pT2	76	Male	Training/Discovery Phase
87773	Bca case	pT2	76	Male	Training/Discovery Phase
87831	Bca case	pT2	76	Male	Training/Discovery Phase
87778	Bca case	pT4	76	Male	Training/Discovery Phase
19874	Bca case	pTa	76	Male	Training/Discovery Phase
27962	Bca case	pTa	76	Male	Training/Discovery Phase
82094	Bca case	pTa	76	Male	Training/Discovery Phase
85236	Bca case	pTa	76	Male	Training/Discovery Phase
85814	Bca case	pTa	76	Male	Training/Discovery Phase
86808	Bca case	pTa	76	Male	Training/Discovery Phase
86613	Bca case	pT1	77	Male	Training/Discovery Phase
69666	Bca case	pT2	77	Male	Training/Discovery Phase
81363	Bca case	pT2	77	Male	Training/Discovery Phase
86608	Bca case	pT2	77	Male	Training/Discovery Phase
87280	Bca case	pT2	77	Male	Training/Discovery Phase
21975	Bca case	pT3	77	Male	Training/Discovery Phase
27443	Bca case	pTa	77	Male	Training/Discovery Phase
82310	Bca case	pTa	77	Male	Training/Discovery Phase
88125	Bca case	pTa	77	Male	Training/Discovery Phase
86211	Bca case	pT2	78	Male	Training/Discovery Phase
86363	Bca case	pTa	78	Male	Training/Discovery Phase
86810	Bca case	pTa	78	Male	Training/Discovery Phase
87217	Bca case	pTa	78	Male	Training/Discovery Phase
88133	Bca case	pTa	78	Male	Training/Discovery Phase
84980	Bca case	pT1	79	Male	Training/Discovery Phase
86588	Bca case	pT1	79	Male	Training/Discovery Phase
80619	Bca case	pTa	79	Male	Training/Discovery Phase

CE-MS Analysis ID	Diagnosis	Stage	Age	Gender	Analytical Phase
85990	Bca case	pTa	79	Male	Training/Discovery Phase
86197	Bca case	pTa	79	Male	Training/Discovery Phase
88122	Bca case	pTa	79	Male	Training/Discovery Phase
88124	Bca case	pTa	79	Male	Training/Discovery Phase
80651	Bca case	pT1	80	Male	Training/Discovery Phase
80656	Bca case	pT1	80	Male	Training/Discovery Phase
80636	Bca case	pT2	80	Male	Training/Discovery Phase
80637	Bca case	pT2	80	Male	Training/Discovery Phase
82083	Bca case	pTa	80	Male	Training/Discovery Phase
85021	Bca case	pTa	80	Male	Training/Discovery Phase
88103	Bca case	pTa	80	Male	Training/Discovery Phase
86364	Bca case	pTis	80	Male	Training/Discovery Phase
86809	Bca case	pT1	81	Male	Training/Discovery Phase
87054	Bca case	pT1	81	Male	Training/Discovery Phase
27074	Bca case	pT3	81	Male	Training/Discovery Phase
85000	Bca case	pTa	81	Male	Training/Discovery Phase
86196	Bca case	pTa	81	Male	Training/Discovery Phase
19870	Bca case	pT2	82	Male	Training/Discovery Phase
85199	Bca case	pTa	82	Male	Training/Discovery Phase
87315	Bca case	pTa	82	Male	Training/Discovery Phase
27951	Bca case	pT1	83	Male	Training/Discovery Phase
80630	Bca case	pT1	83	Male	Training/Discovery Phase
86019	Bca case	pT1	83	Male	Training/Discovery Phase
86215	Bca case	pT1	83	Male	Training/Discovery Phase
86719	Bca case	pT1	83	Male	Training/Discovery Phase
85987	Bca case	pTa	83	Male	Training/Discovery Phase
86235	Bca case	pTa	83	Male	Training/Discovery Phase
85022	Bca case	pT1	84	Male	Training/Discovery Phase
27081	Bca case	pT2	84	Male	Training/Discovery Phase
88135	Bca case	pT2	84	Male	Training/Discovery Phase
85818	Bca case	pTa	84	Male	Training/Discovery Phase
86003	Bca case	pTis	84	Male	Training/Discovery Phase
85992	Bca case	pT1	85	Male	Training/Discovery Phase
19865	Bca case	pT2	85	Male	Training/Discovery Phase
87833	Bca case	pT2	85	Male	Training/Discovery Phase
85029	Bca case	pT1	86	Male	Training/Discovery Phase
88120	Bca case	pT1	86	Male	Training/Discovery Phase
87776	Bca case	pT2	86	Male	Training/Discovery Phase
86593	Bca case	pTa	86	Male	Training/Discovery Phase
84962	Bca case	pTa	87	Male	Training/Discovery Phase
86708	Bca case	pT2	88	Male	Training/Discovery Phase
84952	Bca case	pTa	88	Male	Training/Discovery Phase
81304	Bca case	pT1	89	Male	Training/Discovery Phase
86811	Bca case	pT1	95	Male	Training/Discovery Phase
27967	Bca case	pTa	83		Training/Discovery Phase
19962	Urological Control		34	Female	Test/Validation Phase
20890	Urological Control		36	Female	Test/Validation Phase
20891	Urological Control		41	Female	Test/Validation Phase
20913	Urological Control		46	Female	Test/Validation Phase

CE-MS Analysis ID	Diagnosis	Stage	Age	Gender	Analytical Phase
20938	Urological Control		55	Female	Test/Validation Phase
21033	Urological Control		63	Female	Test/Validation Phase
21524	Urological Control		68	Female	Test/Validation Phase
21694	Urological Control		78	Female	Test/Validation Phase
17256	Urological Control		33	Male	Test/Validation Phase
20937	Urological Control		48	Male	Test/Validation Phase
20941	Urological Control		60	Male	Test/Validation Phase
21091	Urological Control		63	Male	Test/Validation Phase
21155	Urological Control		64	Male	Test/Validation Phase
21251	Urological Control		65	Male	Test/Validation Phase
21620	Urological Control		69	Male	Test/Validation Phase
21693	Urological Control		70	Male	Test/Validation Phase
21708	Urological Control		78	Male	Test/Validation Phase
21740	Urological Control		83	Male	Test/Validation Phase
21750	Urological Control		85	Male	Test/Validation Phase
87043	Urological Control		85	Male	Test/Validation Phase
87214	Urological Control		86	Male	Test/Validation Phase
87448	Urological Control		86	Male	Test/Validation Phase
87640	Urological Control		93	Male	Test/Validation Phase
27921	BCa case	pT2	51	Female	Test/Validation Phase
27974	BCa case	pT1	52	Female	Test/Validation Phase
27986	BCa case	pT2	53	Female	Test/Validation Phase
27998	BCa case	pT1	54	Female	Test/Validation Phase
87030	BCa case	pT1	54	Female	Test/Validation Phase
87035	BCa case	pT1	54	Female	Test/Validation Phase
87066	BCa case	pTa	54	Female	Test/Validation Phase
87077	BCa case	pTa	54	Female	Test/Validation Phase
87106	BCa case	pT2	56	Female	Test/Validation Phase
19839	BCa case	pT3	59	Female	Test/Validation Phase
20734	BCa case	pT2	60	Female	Test/Validation Phase
27949	BCa case	pTa	62	Female	Test/Validation Phase
27961	BCa case	pTa	62	Female	Test/Validation Phase
27991	BCa case	pTa	63	Female	Test/Validation Phase
87029	BCa case	pT1	64	Female	Test/Validation Phase
87031	BCa case	pTa	64	Female	Test/Validation Phase
87079	BCa case	pT3	65	Female	Test/Validation Phase
87190	BCa case	pTis	65	Female	Test/Validation Phase
87461	BCa case	pTa	66	Female	Test/Validation Phase
19898	BCa case	pTa	67	Female	Test/Validation Phase
19903	BCa case	pTa	67	Female	Test/Validation Phase
17473	BCa case	pT3	70	Female	Test/Validation Phase
17567	BCa case	pT4	70	Female	Test/Validation Phase
27671	BCa case	pTa	73	Female	Test/Validation Phase
27739	BCa case	pTa	74	Female	Test/Validation Phase
27982	BCa case	pTa	76	Female	Test/Validation Phase
87203	BCa case	pTa	79	Female	Test/Validation Phase
19844	BCa case	pTa	86	Female	Test/Validation Phase
17751	BCa case	pT2	27	Male	Test/Validation Phase
17201	Bca case	pTa	48	Male	Test/Validation Phase

CE-MS Analysis ID	Diagnosis	Stage	Age	Gender	Analytical Phase
27726	BCa case	pT1	49	Male	Test/Validation Phase
27735	BCa case	pTa	49	Male	Test/Validation Phase
27923	BCa case	pTa	51	Male	Test/Validation Phase
27987	BCa case	pTa	53	Male	Test/Validation Phase
27988	BCa case	pTa	53	Male	Test/Validation Phase
27997	BCa case	pTis	53	Male	Test/Validation Phase
87044	BCa case	pT2	54	Male	Test/Validation Phase
87078	BCa case	pT1	55	Male	Test/Validation Phase
87080	BCa case	pTa	55	Male	Test/Validation Phase
87105	BCa case	pT2	56	Male	Test/Validation Phase
87123	BCa case	pTa	56	Male	Test/Validation Phase
87585	BCa case	pT2	57	Male	Test/Validation Phase
87631	BCa case	pT2	58	Male	Test/Validation Phase
87701	BCa case	pTa	58	Male	Test/Validation Phase
19899	BCa case	pT1	60	Male	Test/Validation Phase
20741	BCa case	pT2	60	Male	Test/Validation Phase
27686	BCa case	pTa	60	Male	Test/Validation Phase
27697	BCa case	pTa	60	Male	Test/Validation Phase
27711	BCa case	pTa	60	Male	Test/Validation Phase
27925	BCa case	pT1	61	Male	Test/Validation Phase
27952	BCa case	pTa	62	Male	Test/Validation Phase
27969	BCa case	pT1	63	Male	Test/Validation Phase
27970	BCa case	pTa	63	Male	Test/Validation Phase
27992	BCa case	pTa	63	Male	Test/Validation Phase
32560	BCa case	pTa	63	Male	Test/Validation Phase
87045	BCa case	pTa	64	Male	Test/Validation Phase
87074	BCa case	pT1	65	Male	Test/Validation Phase
87125	BCa case	pTa	65	Male	Test/Validation Phase
87195	BCa case	pT2	66	Male	Test/Validation Phase
87242	BCa case	pT3	66	Male	Test/Validation Phase
87244	BCa case	pTa	66	Male	Test/Validation Phase
87271	BCa case	pTa	66	Male	Test/Validation Phase
87664	BCa case	pT1	67	Male	Test/Validation Phase
10264	BCa case	pT2	67	Male	Test/Validation Phase
20074	BCa case	pT3	68	Male	Test/Validation Phase
21707	BCa case	pTa	68	Male	Test/Validation Phase
26567	BCa case	pT1	69	Male	Test/Validation Phase
27724	BCa case	pT2	69	Male	Test/Validation Phase
27847	BCa case	pT2	69	Male	Test/Validation Phase
27953	BCa case	pTa	69	Male	Test/Validation Phase
27955	BCa case	pT2	70	Male	Test/Validation Phase
27956	BCa case	pT2	70	Male	Test/Validation Phase
27960	BCa case	pT2	70	Male	Test/Validation Phase
28010	BCa case	pT3	70	Male	Test/Validation Phase
27764	BCa case	pT1	71	Male	Test/Validation Phase
27766	BCa case	pT1	71	Male	Test/Validation Phase
27604	BCa case	pT2	71	Male	Test/Validation Phase
27606	BCa case	pTa	71	Male	Test/Validation Phase
27609	BCa case	pT1	72	Male	Test/Validation Phase

CE-MS Analysis ID	Diagnosis	Stage	Age	Gender	Analytical Phase
27610	BCa case	pTa	72	Male	Test/Validation Phase
27611	BCa case	pTis	72	Male	Test/Validation Phase
27652	BCa case	pT1	73	Male	Test/Validation Phase
27670	BCa case	pT3	73	Male	Test/Validation Phase
27723	BCa case	pT1	74	Male	Test/Validation Phase
27725	BCa case	pT1	74	Male	Test/Validation Phase
27727	BCa case	pT3	74	Male	Test/Validation Phase
27738	BCa case	pT4	74	Male	Test/Validation Phase
27751	BCa case	pTa	74	Male	Test/Validation Phase
27805	BCa case	pTis	74	Male	Test/Validation Phase
27813	BCa case	pT2	75	Male	Test/Validation Phase
27922	BCa case	pTa	75	Male	Test/Validation Phase
27927	BCa case	pTa	75	Male	Test/Validation Phase
27976	BCa case	pTa	75	Male	Test/Validation Phase
27977	BCa case	pT2	76	Male	Test/Validation Phase
27978	BCa case	pT2	76	Male	Test/Validation Phase
27979	BCa case	pT2	76	Male	Test/Validation Phase
27980	BCa case	pT3	76	Male	Test/Validation Phase
27981	BCa case	pT3	76	Male	Test/Validation Phase
27984	BCa case	pTa	76	Male	Test/Validation Phase
86821	BCa case	pTa	76	Male	Test/Validation Phase
86901	BCa case	pTis	76	Male	Test/Validation Phase
87017	BCa case	pT1	77	Male	Test/Validation Phase
87028	BCa case	pT2	77	Male	Test/Validation Phase
87033	BCa case	pT2	77	Male	Test/Validation Phase
87047	BCa case	pTa	77	Male	Test/Validation Phase
87075	BCa case	pTa	77	Male	Test/Validation Phase
87076	BCa case	pT2	78	Male	Test/Validation Phase
87108	BCa case	pT2	78	Male	Test/Validation Phase
87126	BCa case	pT3	78	Male	Test/Validation Phase
87130	BCa case	pT3	78	Male	Test/Validation Phase
87133	BCa case	pTa	79	Male	Test/Validation Phase
87199	BCa case	pTa	79	Male	Test/Validation Phase
87200	BCa case	pTa	79	Male	Test/Validation Phase
87204	BCa case	pTa	79	Male	Test/Validation Phase
87232	BCa case	pT2	80	Male	Test/Validation Phase
87235	BCa case	pT3	80	Male	Test/Validation Phase
87240	BCa case	pTa	80	Male	Test/Validation Phase
87270	BCa case	pTa	80	Male	Test/Validation Phase
87358	BCa case	pTis	80	Male	Test/Validation Phase
87446	BCa case	pT1	81	Male	Test/Validation Phase
87460	BCa case	pT2	81	Male	Test/Validation Phase
87485	BCa case	pT3	81	Male	Test/Validation Phase
87580	BCa case	pTa	81	Male	Test/Validation Phase
87582	BCa case	pT2	82	Male	Test/Validation Phase
87584	BCa case	pTa	82	Male	Test/Validation Phase
87587	BCa case	pT1	83	Male	Test/Validation Phase
87622	BCa case	pT1	83	Male	Test/Validation Phase
87669	BCa case	pTa	83	Male	Test/Validation Phase

CE-MS Analysis ID	Diagnosis	Stage	Age	Gender	Analytical Phase
87672	BCa case	pTa	83	Male	Test/Validation Phase
87677	BCa case	pTa	83	Male	Test/Validation Phase
87702	BCa case	pTa	83	Male	Test/Validation Phase
87713	BCa case	pTa	83	Male	Test/Validation Phase
17255	BCa case	pTa	85	Male	Test/Validation Phase
92595	BCa case	pTa	85	Male	Test/Validation Phase
19835	BCa case	pT1	86	Male	Test/Validation Phase
19836	BCa case	pT2	86	Male	Test/Validation Phase
19901	BCa case	pTa	86	Male	Test/Validation Phase
87081	BCa case	pTa	87	Male	Test/Validation Phase

Abbreviations: BCa, Bladder Cancer

Supplementary Table S4. **Spreadsheet 2**. Clinical and demographical data of the prostate cancer cohort (n=385).

CE-MS Analysis ID	Diagnosis	Stage	Age	PSA level	Gleason Score	Analytical Phase
108669	BPH		70	0,6		Training/Discovery Phase
108859	BPH		66	1		Training/Discovery Phase
108911	BPH		70	2,3		Training/Discovery Phase
108951	BPH		67	2,8		Training/Discovery Phase
70492	BPH		67	3,05		Training/Discovery Phase
25528	BPH		69	3,1		Training/Discovery Phase
70491	BPH		56	3,38		Training/Discovery Phase
48785	BPH		55	3,54		Training/Discovery Phase
45192	BPH		59	3,59		Training/Discovery Phase
28104	BPH		69	3,73		Training/Discovery Phase
108256	BPH		68	3,79		Training/Discovery Phase
70489	BPH		65	3,84		Training/Discovery Phase
27846	BPH		62	3,89		Training/Discovery Phase
70551	BPH		68	3,99		Training/Discovery Phase
27939	BPH		75	4,1		Training/Discovery Phase
70476	BPH		54	4,2		Training/Discovery Phase
108963	BPH		75	4,3		Training/Discovery Phase
70450	BPH		56	4,4		Training/Discovery Phase
25728	BPH		67	4,52		Training/Discovery Phase
70456	BPH		59	4,7		Training/Discovery Phase
108941	BPH		73	4,7		Training/Discovery Phase
69694	BPH		69	4,71		Training/Discovery Phase
26008	BPH		71	4,8		Training/Discovery Phase
108778	BPH		58	4,89		Training/Discovery Phase
25609	BPH		63	5,06		Training/Discovery Phase
26017	BPH		68	5,08		Training/Discovery Phase
70446	BPH		68	5,08		Training/Discovery Phase
108262	BPH		65	5,1		Training/Discovery Phase
108902	BPH		75	5,1		Training/Discovery Phase
20632	BPH		73	5,2		Training/Discovery Phase
108282	BPH		58	5,25		Training/Discovery Phase
78724	BPH		72	5,3		Training/Discovery Phase
25800	BPH		61	5,33		Training/Discovery Phase
83250	BPH		76	5,4		Training/Discovery Phase
70484	BPH		78	5,4		Training/Discovery Phase
25594	BPH		70	5,55		Training/Discovery Phase
70567	BPH		71	5,6		Training/Discovery Phase
70452	BPH		76	5,6		Training/Discovery Phase
20616	BPH		68	5,66		Training/Discovery Phase
25627	BPH		60	5,7		Training/Discovery Phase
108805	BPH		80	5,7		Training/Discovery Phase
108283	BPH		60	5,8		Training/Discovery Phase
108266	BPH		58	5,9		Training/Discovery Phase
78757	BPH		71	5,9		Training/Discovery Phase
28100	BPH		69	5,98		Training/Discovery Phase
70459	BPH		71	6		Training/Discovery Phase
70565	BPH		55	6,08		Training/Discovery Phase

CE-MS Analysis ID	Diagnosis	Stage	Age	PSA level	Gleason Score	Analytical Phase
22701	BPH		67	6,16		Training/Discovery Phase
108971	BPH		71	6,2		Training/Discovery Phase
108748	BPH		75	6,2		Training/Discovery Phase
70418	BPH		87	6,4		Training/Discovery Phase
70481	BPH		58	6,41		Training/Discovery Phase
70349	BPH		51	6,45		Training/Discovery Phase
108823	BPH		79	6,5		Training/Discovery Phase
22826	BPH		73	6,6		Training/Discovery Phase
70560	BPH		57	6,62		Training/Discovery Phase
70463	BPH		63	6,63		Training/Discovery Phase
79039	BPH		70	6,7		Training/Discovery Phase
25074	BPH		57	6,72		Training/Discovery Phase
70445	BPH		52	6,96		Training/Discovery Phase
78799	BPH		65	7,2		Training/Discovery Phase
78978	BPH		64	7,22		Training/Discovery Phase
26011	BPH		63	7,27		Training/Discovery Phase
33073	BPH		74	7,3		Training/Discovery Phase
108975	BPH		74	7,4		Training/Discovery Phase
108249	BPH		63	7,58		Training/Discovery Phase
108830	BPH		68	7,58		Training/Discovery Phase
83362	BPH		80	7,6		Training/Discovery Phase
32609	BPH		79	7,8		Training/Discovery Phase
109000	BPH		62	7,82		Training/Discovery Phase
43564	BPH		62	8,03		Training/Discovery Phase
70570	BPH		64	8,18		Training/Discovery Phase
70470	BPH		78	8,2		Training/Discovery Phase
108314	BPH		74	8,5		Training/Discovery Phase
108862	BPH		66	8,61		Training/Discovery Phase
70333	BPH		65	8,71		Training/Discovery Phase
70430	BPH		81	8,8		Training/Discovery Phase
45186	BPH		72	8,9		Training/Discovery Phase
70417	BPH		56	9,1		Training/Discovery Phase
70441	BPH		52	9,13		Training/Discovery Phase
108744	BPH		68	9,4		Training/Discovery Phase
70482	BPH		77	9,5		Training/Discovery Phase
28004	BPH		60	9,7		Training/Discovery Phase
108393	BPH		84	9,7		Training/Discovery Phase
70465	BPH		51	9,89		Training/Discovery Phase
108967	BPH		77	10		Training/Discovery Phase
108787	BPH		63	10,34		Training/Discovery Phase
28602	BPH		62	10,56		Training/Discovery Phase
23046	BPH		65	10,56		Training/Discovery Phase
45194	BPH		71	10,8		Training/Discovery Phase
27625	BPH		58	11,3		Training/Discovery Phase
79002	BPH		63	11,7		Training/Discovery Phase
78701	BPH		70	11,82		Training/Discovery Phase
32836	BPH		67	11,9		Training/Discovery Phase
70458	BPH		69	11,9		Training/Discovery Phase

CE-MS Analysis ID	Diagnosis	Stage	Age	PSA level	Gleason Score	Analytical Phase
25742	BPH		71	12,4		Training/Discovery Phase
45147	BPH		72	12,8		Training/Discovery Phase
79070	BPH		60	13,25		Training/Discovery Phase
25562	BPH		74	13,4		Training/Discovery Phase
45184	BPH		72	13,9		Training/Discovery Phase
108905	BPH		65	14,4		Training/Discovery Phase
83420	BPH		71	14,6		Training/Discovery Phase
108670	BPH		83	15		Training/Discovery Phase
108258	BPH		67	15,06		Training/Discovery Phase
108263	BPH		54	16,9		Training/Discovery Phase
51385	BPH		61	17,55		Training/Discovery Phase
108833	BPH		72	18,3		Training/Discovery Phase
24196	BPH		73	18,8		Training/Discovery Phase
45142	BPH		61	19,78		Training/Discovery Phase
26010	BPH		58	20,56		Training/Discovery Phase
45151	BPH		57	21,7		Training/Discovery Phase
22863	BPH		59	24		Training/Discovery Phase
25885	BPH		72	28		Training/Discovery Phase
108253	BPH		69	28,6		Training/Discovery Phase
108261	BPH		62	30,61		Training/Discovery Phase
108983	BPH		70	63,8		Training/Discovery Phase
108978	BPH		56	72,5		Training/Discovery Phase
26065	BPH		54			Training/Discovery Phase
25603	BPH		55			Training/Discovery Phase
108747	BPH		62			Training/Discovery Phase
70586	BPH		64			Training/Discovery Phase
108934	BPH		64			Training/Discovery Phase
43870	BPH		65			Training/Discovery Phase
17290	BPH		65			Training/Discovery Phase
45198	BPH		65			Training/Discovery Phase
108251	BPH		65			Training/Discovery Phase
108342	BPH		65			Training/Discovery Phase
108974	BPH		65			Training/Discovery Phase
79096	BPH		65			Training/Discovery Phase
25707	BPH		67			Training/Discovery Phase
83520	PCa case	pT3	53	1,61		Training/Discovery Phase
70569	PCa case		65	2,53		Training/Discovery Phase
70525	PCa case		60	3,03		Training/Discovery Phase
70585	PCa case		65	3,27		Training/Discovery Phase
26122	PCa case		60	3,58		Training/Discovery Phase
108972	PCa case		66	3,6		Training/Discovery Phase
78694	PCa case		65	3,8		Training/Discovery Phase
83141	PCa case		62	3,89		Training/Discovery Phase
83143	PCa case		61	4,07		Training/Discovery Phase
70528	PCa case		68	4,17		Training/Discovery Phase
83496	PCa case	pT3	64	4,49		Training/Discovery Phase
108779	PCa case		68	4,76		Training/Discovery Phase
83267	PCa case	pT2	53	4,77		Training/Discovery Phase
108749	PCa case	pT4	59	4,81		Training/Discovery Phase

CE-MS Analysis ID	Diagnosis	Stage	Age	PSA level	Gleason Score	Analytical Phase
43862	PCa case		73	4,9	6	Training/Discovery Phase
108428	PCa case		82	5	6	Training/Discovery Phase
70679	PCa case		70	5,5	7	Training/Discovery Phase
70468	PCa case		71	5,8	7	Training/Discovery Phase
83345	PCa case	pT3	75	5,8	7	Training/Discovery Phase
70451	PCa case		69	5,8		Training/Discovery Phase
108341	PCa case		73	5,9	7	Training/Discovery Phase
70595	PCa case		68	5,97		Training/Discovery Phase
108863	PCa case		72	6	7	Training/Discovery Phase
108746	PCa case	pT3	70	6,2	6	Training/Discovery Phase
45197	PCa case		65	6,2		Training/Discovery Phase
32697	PCa case		54	6,38		Training/Discovery Phase
23234	PCa case		66	6,4		Training/Discovery Phase
70668	PCa case		78	6,5	7	Training/Discovery Phase
108826	PCa case		64	6,75		Training/Discovery Phase
45191	PCa case		71	6,8	6	Training/Discovery Phase
28452	PCa case		74	6,8		Training/Discovery Phase
69688	PCa case		69	6,88		Training/Discovery Phase
70575	PCa case		70	7,1	7	Training/Discovery Phase
108976	PCa case		72	7,1	7	Training/Discovery Phase
45149	PCa case		73	7,2	7	Training/Discovery Phase
20634	PCa case		67	7,2		Training/Discovery Phase
70580	PCa case		61	7,3		Training/Discovery Phase
108790	PCa case		68	7,34		Training/Discovery Phase
43754	PCa case	pT2	65	7,4		Training/Discovery Phase
108827	PCa case		69	7,5	7	Training/Discovery Phase
83522	PCa case		74	7,5	7	Training/Discovery Phase
25544	PCa case		68	7,67		Training/Discovery Phase
70566	PCa case		69	7,92		Training/Discovery Phase
83521	PCa case	pT4	75	8	7	Training/Discovery Phase
32539	PCa case		68	8,01		Training/Discovery Phase
70527	PCa case		68	8,09		Training/Discovery Phase
108357	PCa case		73	8,1	7	Training/Discovery Phase
83262	PCa case	pT3	82	8,1	6	Training/Discovery Phase
83208	PCa case	pT3	74	8,2	7	Training/Discovery Phase
83342	PCa case		76	8,2	6	Training/Discovery Phase
70670	PCa case		69	8,2		Training/Discovery Phase
108829	PCa case		67	8,49		Training/Discovery Phase
43572	PCa case	PT1	74	8,6	7	Training/Discovery Phase
108836	PCa case		63	8,75		Training/Discovery Phase
83344	PCa case	pT4	87	8,8	6	Training/Discovery Phase
45156	PCa case		72	8,9	6	Training/Discovery Phase
70665	PCa case		72	9	7	Training/Discovery Phase
45202	PCa case		73	9,2	7	Training/Discovery Phase
70579	PCa case		75	9,3	6	Training/Discovery Phase
83268	PCa case	pT3	75	9,3	7	Training/Discovery Phase
108344	PCa case		73	9,3		Training/Discovery Phase
108822	PCa case		66	9,4		Training/Discovery Phase

CE-MS Analysis ID	Diagnosis	Stage	Age	PSA level	Gleason Score	Analytical Phase
83269	PCa case		77	9,5	7	Training/Discovery Phase
70485	PCa case		79	9,5	9	Training/Discovery Phase
108285	PCa case		69	9,72		Training/Discovery Phase
83145	PCa case	pT2	66	10		Training/Discovery Phase
83356	PCa case		61	10,03		Training/Discovery Phase
43601	PCa case	pT3	59	10,04		Training/Discovery Phase
70419	PCa case		56	10,16		Training/Discovery Phase
45193	PCa case		73	10,4	6	Training/Discovery Phase
70654	PCa case		57	10,55		Training/Discovery Phase
108696	PCa case		60	10,86		Training/Discovery Phase
83225	PCa case		64	11,2		Training/Discovery Phase
108315	PCa case	pT2	86	11,4	9	Training/Discovery Phase
83508	PCa case	pT2	63	11,48		Training/Discovery Phase
108742	PCa case	pT3	71	11,5	7	Training/Discovery Phase
70671	PCa case		74	11,5	6	Training/Discovery Phase
83237	PCa case	pT4	76	11,5	7	Training/Discovery Phase
70563	PCa case		59	11,79		Training/Discovery Phase
24968	PCa case		72	12	7	Training/Discovery Phase
70433	PCa case		48	12,03		Training/Discovery Phase
22820	PCa case		68	12,3		Training/Discovery Phase
45139	PCa case		58	12,6		Training/Discovery Phase
70475	PCa case		54	13,1		Training/Discovery Phase
108912	PCa case		78	13,3	7	Training/Discovery Phase
108966	PCa case		66	13,55		Training/Discovery Phase
83263	PCa case		71	14,3	9	Training/Discovery Phase
108407	PCa case		58	14,5		Training/Discovery Phase
20633	PCa case		73	15	7	Training/Discovery Phase
45183	PCa case		73	15	6	Training/Discovery Phase
83146	PCa case	pT3	77	15,5		Training/Discovery Phase
70499	PCa case		76	15,7	9	Training/Discovery Phase
70479	PCa case		45	16,02		Training/Discovery Phase
43556	PCa case	pT2	61	16,39		Training/Discovery Phase
83488	PCa case		66	16,66		Training/Discovery Phase
32829	PCa case		65	16,96		Training/Discovery Phase
70474	PCa case		61	17,19		Training/Discovery Phase
55827	PCa case		71	17,3	9	Training/Discovery Phase
70341	PCa case		60	17,56		Training/Discovery Phase
27658	PCa case		64	17,7		Training/Discovery Phase
108973	PCa case		74	19,9		Training/Discovery Phase
44011	PCa case		63	20,21		Training/Discovery Phase
83444	PCa case		77	23,7	9	Training/Discovery Phase
83261	PCa case	pT4	86	25,2	7	Training/Discovery Phase
108743	PCa case	pT4	76	26,9	7	Training/Discovery Phase
45201	PCa case		68	31,42		Training/Discovery Phase
83122	PCa case	pT2	66	31,5		Training/Discovery Phase
108745	PCa case	pT3	71	33,4	7	Training/Discovery Phase
83446	PCa case	pT4	79	37	9	Training/Discovery Phase
108832	PCa case		64	40,31		Training/Discovery Phase
45195	PCa case		71	42,2	9	Training/Discovery Phase

CE-MS Analysis ID	Diagnosis	Stage	Age	PSA level	Gleason Score	Analytical Phase
45185	PCa case		74	44,4	9	Training/Discovery Phase
70592	PCa case		75	44,4	9	Training/Discovery Phase
70483	PCa case		41	45,35		Training/Discovery Phase
70453	PCa case		72	58,1	7	Training/Discovery Phase
108340	PCa case	pT2	85	66	9	Training/Discovery Phase
83142	PCa case		71	71	8	Training/Discovery Phase
70589	PCa case		62	110		Training/Discovery Phase
70478	PCa case		75	163,1		Training/Discovery Phase
70554	PCa case		71	217		Training/Discovery Phase
83215	PCa case	pT3	75	236,9		Training/Discovery Phase
83343	PCa case	pT3	76	329,7		Training/Discovery Phase
70423	PCa case		76	397		Training/Discovery Phase
45143	PCa case		76	850,5		Training/Discovery Phase
25642	PCa case		73	987		Training/Discovery Phase
70494	PCa case		45			Training/Discovery Phase
70448	PCa case		56			Training/Discovery Phase
45153	PCa case		68			Training/Discovery Phase
108985	PCa case		64			Training/Discovery Phase
83341	PCa case	pT3	65			Training/Discovery Phase
45148	PCa case		65			Training/Discovery Phase
70653	PCa case		66			Training/Discovery Phase
83147	PCa case	pT2	67			Training/Discovery Phase
83450	PCa case	pT3	67			Training/Discovery Phase
70469	PCa case		67			Training/Discovery Phase
108821	PCa case		67			Training/Discovery Phase
70462	PCa case		69			Training/Discovery Phase
83381	PCa case		69			Training/Discovery Phase
20617	BPH		69	12,5		Test/Validation Phase
20620	BPH		71	8,83		Test/Validation Phase
20635	BPH		65	3,34		Test/Validation Phase
23028	BPH		70	11,15		Test/Validation Phase
23537	BPH		69	7		Test/Validation Phase
24805	BPH		68	8,2		Test/Validation Phase
24969	BPH		65	7,29		Test/Validation Phase
25611	BPH		74	5,96		Test/Validation Phase
25630	BPH		53	6,27		Test/Validation Phase
25694	BPH		74	5,7		Test/Validation Phase
25865	BPH		62	24,57		Test/Validation Phase
25942	BPH		61	8,24		Test/Validation Phase
25994	BPH		65	18,32		Test/Validation Phase
27594	BPH		69	10,5		Test/Validation Phase
27715	BPH		56	5,4		Test/Validation Phase
27758	BPH		62	3,05		Test/Validation Phase
28001	BPH		83	4,6		Test/Validation Phase
28222	BPH		70			Test/Validation Phase
28455	BPH		51	5,6		Test/Validation Phase
28456	BPH		71	5,4		Test/Validation Phase
28516	BPH		73	12,48		Test/Validation Phase

CE-MS Analysis ID	Diagnosis	Stage	Age	PSA level	Gleason Score	Analytical Phase
28520	BPH		71	5,5		Test/Validation Phase
28581	BPH		55	4,39		Test/Validation Phase
32814	BPH		61	5,25		Test/Validation Phase
45146	BPH		67	3,6		Test/Validation Phase
45189	BPH		56			Test/Validation Phase
45199	BPH		63	6,25		Test/Validation Phase
45200	BPH		65	7,36		Test/Validation Phase
70432	BPH		76	32,1	7	Test/Validation Phase
70454	BPH		72	12,6	9	Test/Validation Phase
70457	BPH		68			Test/Validation Phase
70461	BPH		64	6,68		Test/Validation Phase
70464	BPH		70	5,31		Test/Validation Phase
70467	BPH		67			Test/Validation Phase
70471	BPH		65	10,5		Test/Validation Phase
70480	BPH		55	5,69		Test/Validation Phase
70487	BPH		67	6,11		Test/Validation Phase
70488	BPH		73	8,3	7	Test/Validation Phase
70490	BPH		61	6,66		Test/Validation Phase
70500	BPH		67			Test/Validation Phase
70559	BPH		75	384		Test/Validation Phase
70578	BPH		69	6,1		Test/Validation Phase
70583	BPH		68	7,06		Test/Validation Phase
70672	BPH		73	20,1	7	Test/Validation Phase
79011	BPH		69			Test/Validation Phase
79669	BPH		63	4,89		Test/Validation Phase
108672	BPH		76	28,8	7	Test/Validation Phase
108901	BPH		66	9,5		Test/Validation Phase
108909	BPH		71	4,1	6	Test/Validation Phase
19386	PCa case		64	6,54		Test/Validation Phase
22916	PCa case		79	4,7		Test/Validation Phase
24894	PCa case		68	5,2		Test/Validation Phase
25729	PCa case		60	3,98		Test/Validation Phase
25982	PCa case		67	6,83		Test/Validation Phase
27662	PCa case		58	8,48		Test/Validation Phase
28270	PCa case		63	7,38		Test/Validation Phase
32795	PCa case		64	7,63		Test/Validation Phase
43600	PCa case		58	3,87		Test/Validation Phase
43751	PCa case		75	18,42		Test/Validation Phase
43752	PCa case		73	18,9		Test/Validation Phase
43861	PCa case		74	5,53		Test/Validation Phase
45145	PCa case		56	1,22		Test/Validation Phase
45150	PCa case		66	7,43		Test/Validation Phase
45152	PCa case		81	4,9		Test/Validation Phase
45154	PCa case		65	20,5		Test/Validation Phase
45187	PCa case		62	5,26		Test/Validation Phase
45188	PCa case		72	6,9		Test/Validation Phase
45190	PCa case		67	7		Test/Validation Phase
70421	PCa case		60	4,34		Test/Validation Phase
70422	PCa case		54	10,01		Test/Validation Phase

CE-MS Analysis ID	Diagnosis	Stage	Age	PSA level	Gleason Score	Analytical Phase
70440	PCa case		71			Test/Validation Phase
70455	PCa case		73	17,2	7	Test/Validation Phase
70460	PCa case		57	9,56		Test/Validation Phase
70473	PCa case	pT2	59			Test/Validation Phase
70486	PCa case		71	4,47		Test/Validation Phase
70498	PCa case		59	7,02	7	Test/Validation Phase
70526	PCa case		66	2,42		Test/Validation Phase
70552	PCa case		78	5	6	Test/Validation Phase
70556	PCa case		45	17,4		Test/Validation Phase
70568	PCa case		76	7,8	7	Test/Validation Phase
70574	PCa case		67	12,89		Test/Validation Phase
70581	PCa case		74	136		Test/Validation Phase
70587	PCa case		73	10,9	6	Test/Validation Phase
70588	PCa case		73	21,5	9	Test/Validation Phase
70590	PCa case		63	9,25		Test/Validation Phase
70593	PCa case		79	13,3	7	Test/Validation Phase
70596	PCa case		54	5,51		Test/Validation Phase
70598	PCa case		53	8,34		Test/Validation Phase
70666	PCa case		60	6,13		Test/Validation Phase
78716	PCa case		69	8,12		Test/Validation Phase
83226	PCa case		62	11,2		Test/Validation Phase
83227	PCa case		66	4,13		Test/Validation Phase
83248	PCa case		68			Test/Validation Phase
83280	PCa case		71	13,2	6	Test/Validation Phase
83339	PCa case		58	5,79		Test/Validation Phase
83349	PCa case		70	10,41		Test/Validation Phase
83355	PCa case		61	8,53		Test/Validation Phase
83359	PCa case		75	10,9	9	Test/Validation Phase
83372	PCa case		61	6,39		Test/Validation Phase
83376	PCa case		68			Test/Validation Phase
83404	PCa case		68	10,1		Test/Validation Phase
83423	PCa case		64	3,95		Test/Validation Phase
83424	PCa case		74	16,6	7	Test/Validation Phase
83425	PCa case		66	4,7		Test/Validation Phase
83431	PCa case		86		8	Test/Validation Phase
83436	PCa case		76	7,8	7	Test/Validation Phase
83440	PCa case		65	4,4		Test/Validation Phase
83448	PCa case		74	66,7	7	Test/Validation Phase
83455	PCa case		85	38,2		Test/Validation Phase
83516	PCa case		78	6,2	7	Test/Validation Phase
108388	PCa case		75	21,3	6	Test/Validation Phase
108410	PCa case		72	6	7	Test/Validation Phase
108449	PCa case		72	7,7	7	Test/Validation Phase
108675	PCa case		64	13,19		Test/Validation Phase
108828	PCa case		65	2,2		Test/Validation Phase
108913	PCa case		77	7,4	7	Test/Validation Phase
108977	PCa case		71	6,8	7	Test/Validation Phase

Abbreviations: BPH, Benign Prostatic Hyperplasia; PCa, Prostate Cancer

Supplementary Table S4. Spreadsheet 3. Clinical and demographical data of pancreatic cancer cohort (n=180).

CE-MS Analysis ID	Diagnosis	Age	Gender	CA19-9 level	CEA level	Analytical Phase
115760	Chronic Pancreatitis	27	Male			Training/Discovery Phase
115765	Chronic Pancreatitis	28	Male			Training/Discovery Phase
115660	Chronic Pancreatitis	29	Male			Training/Discovery Phase
115741	Chronic Pancreatitis	34	Female			Training/Discovery Phase
57679	Chronic Pancreatitis	36	Male			Training/Discovery Phase
115744	Chronic Pancreatitis	37	Male			Training/Discovery Phase
115692	Chronic Pancreatitis	40	Male			Training/Discovery Phase
115655	Chronic Pancreatitis	40	Male			Training/Discovery Phase
115663	Chronic Pancreatitis	42	Male			Training/Discovery Phase
115690	Chronic Pancreatitis	43	Male			Training/Discovery Phase
115771	Chronic Pancreatitis	44	Male			Training/Discovery Phase
115656	Chronic Pancreatitis	45	Male			Training/Discovery Phase
115755	Chronic Pancreatitis	45	Male			Training/Discovery Phase
115772	Chronic Pancreatitis	45	Male			Training/Discovery Phase
115764	Chronic Pancreatitis	45	Female			Training/Discovery Phase
115695	Chronic Pancreatitis	46	Female			Training/Discovery Phase
115701	Chronic Pancreatitis	46	Male			Training/Discovery Phase
57673	Chronic Pancreatitis	47	Male	2,9		Training/Discovery Phase
115769	Chronic Pancreatitis	47	Male	3,0	1,0	Training/Discovery Phase
115633	Chronic Pancreatitis	47	Male	5,0	1,0	Training/Discovery Phase
115743	Chronic Pancreatitis	47	Male	10,0	1,0	Training/Discovery Phase
115758	Chronic Pancreatitis	48	Male	16,0	1,0	Training/Discovery Phase
115749	Chronic Pancreatitis	48	Male	37,0	1,0	Training/Discovery Phase
115768	Chronic Pancreatitis	49	Female	3,0	2,0	Training/Discovery Phase
115698	Chronic Pancreatitis	50	Male	4,2	2,0	Training/Discovery Phase
115713	Chronic Pancreatitis	50	Male	5,0	2,0	Training/Discovery Phase
115669	Chronic Pancreatitis	50	Male	6,0	2,0	Training/Discovery Phase
115754	Chronic Pancreatitis	50	Male	7,0	2,0	Training/Discovery Phase
115688	Chronic Pancreatitis	51	Male	8,0	2,0	Training/Discovery Phase
115694	Chronic Pancreatitis	51	Male	8,0	2,0	Training/Discovery Phase
115705	Chronic Pancreatitis	52	Male	9,0	2,0	Training/Discovery Phase
115766	Chronic Pancreatitis	52	Male	11,0	2,0	Training/Discovery Phase
115786	Chronic Pancreatitis	52	Male	15,0	2,0	Training/Discovery Phase
115670	Chronic Pancreatitis	53	Male	30,0	2,0	Training/Discovery Phase
38490	Chronic Pancreatitis	55	Male	2,0	3,0	Training/Discovery Phase
115654	Chronic Pancreatitis	55	Male	10,0	3,0	Training/Discovery Phase
115747	Chronic Pancreatitis	56	Female	10,0	3,0	Training/Discovery Phase
115649	Chronic Pancreatitis	57	Male	14,0	3,0	Training/Discovery Phase
115684	Chronic Pancreatitis	58	Male	18,0	3,0	Training/Discovery Phase
115672	Chronic Pancreatitis	58	Male	19,0	3,0	Training/Discovery Phase
115704	Chronic Pancreatitis	59	Male	23,0	3,0	Training/Discovery Phase
57680	Chronic Pancreatitis	59	Male	86,0	3,0	Training/Discovery Phase
115689	Chronic Pancreatitis	59	Female	22,7	3,1	Training/Discovery Phase
115650	Chronic Pancreatitis	60	Male	1,0	4,0	Training/Discovery Phase
115693	Chronic Pancreatitis	60	Male	5,0	4,0	Training/Discovery Phase
115678	Chronic Pancreatitis	61	Male	8,0	4,0	Training/Discovery Phase

CE-MS Analysis ID	Diagnosis	Age	Gender	CA19-9 level	CEA level	Analytical Phase
115780	Chronic Pancreatitis	61	Female	9,0	4,0	Training/Discovery Phase
115799	Chronic Pancreatitis	61	Male	14,0	4,0	Training/Discovery Phase
115665	Chronic Pancreatitis	61	Male	10,0	5,0	Training/Discovery Phase
115779	Chronic Pancreatitis	63	Female	1,0	6,0	Training/Discovery Phase
115639	Chronic Pancreatitis	66	Male	1,0	6,0	Training/Discovery Phase
115667	Chronic Pancreatitis	68	Male	18,0	6,0	Training/Discovery Phase
115777	Chronic Pancreatitis	69	Male	17,0	7,0	Training/Discovery Phase
115762	Chronic Pancreatitis	72	Male	19,0	7,0	Training/Discovery Phase
115740	Chronic Pancreatitis	73	Male	23,0	7,0	Training/Discovery Phase
115746	Chronic Pancreatitis	73	Female	1,0	8,0	Training/Discovery Phase
115682	Chronic Pancreatitis	75	Male	1,0	8,0	Training/Discovery Phase
115742	Chronic Pancreatitis	77	Male	63,0	9,0	Training/Discovery Phase
115717	Chronic Pancreatitis		Female	1,0	16,0	Training/Discovery Phase
115721	Chronic Pancreatitis		Male	15,0	20,0	Training/Discovery Phase
115733	Chronic Pancreatitis		Male	598,7	21,0	Training/Discovery Phase
115724	Chronic Pancreatitis		Male	1,0	30,0	Training/Discovery Phase
38608	Pancreatic cancer	35	Female			Training/Discovery Phase
115781	Pancreatic cancer	35	Male			Training/Discovery Phase
115789	Pancreatic cancer	43	Male			Training/Discovery Phase
115737	Pancreatic cancer	43	Male			Training/Discovery Phase
115748	Pancreatic cancer	44	Male			Training/Discovery Phase
115734	Pancreatic cancer	46	Male			Training/Discovery Phase
115631	Pancreatic cancer	48	Male			Training/Discovery Phase
115641	Pancreatic cancer	52	Female			Training/Discovery Phase
115681	Pancreatic cancer	53	Male			Training/Discovery Phase
115637	Pancreatic cancer	55	Male			Training/Discovery Phase
115782	Pancreatic cancer	55	Male			Training/Discovery Phase
115803	Pancreatic cancer	56	Male			Training/Discovery Phase
115802	Pancreatic cancer	56	Male			Training/Discovery Phase
115753	Pancreatic cancer	57	Male			Training/Discovery Phase
115643	Pancreatic cancer	58	Male	2,0	1,0	Training/Discovery Phase
115790	Pancreatic cancer	59	Female	5,0	1,0	Training/Discovery Phase
115723	Pancreatic cancer	60	Male	1,0	6,0	Training/Discovery Phase
115800	Pancreatic cancer	60	Female	3,0	6,0	Training/Discovery Phase
115703	Pancreatic cancer	61	Male		9,5	Training/Discovery Phase
115773	Pancreatic cancer	62	Male	2,0	12,0	Training/Discovery Phase
115775	Pancreatic cancer	62	Male	9	12,0	Training/Discovery Phase
115646	Pancreatic cancer	64	Female	2,0	18,0	Training/Discovery Phase
115666	Pancreatic cancer	66	Female	7,0	23,0	Training/Discovery Phase
115709	Pancreatic cancer	67	Male		25,0	Training/Discovery Phase
115719	Pancreatic cancer	67	Female	9,0	31,9	Training/Discovery Phase
115774	Pancreatic cancer	67	Male	917,5	35,0	Training/Discovery Phase
56165	Pancreatic cancer	68	Female		39,5	Training/Discovery Phase
115673	Pancreatic cancer	69	Male		92,0	Training/Discovery Phase
115638	Pancreatic cancer	69	Female	3,0	123,0	Training/Discovery Phase
115644	Pancreatic cancer	69	Female	4,0	186,0	Training/Discovery Phase
56171	Pancreatic cancer	69	Female	30,0	205,0	Training/Discovery Phase
115691	Pancreatic cancer	71	Female	4,0	214,0	Training/Discovery Phase

CE-MS Analysis ID	Diagnosis	Age	Gender	CA19-9 level	CEA level	Analytical Phase
115642	Pancreatic cancer	71	Male	5,0	302,0	Training/Discovery Phase
115657	Pancreatic cancer	71	Female		321,9	Training/Discovery Phase
115784	Pancreatic cancer	72	Female	6,0	327,0	Training/Discovery Phase
115708	Pancreatic cancer	72	Male	5,0	374,0	Training/Discovery Phase
56160	Pancreatic cancer	73	Male	12,0	450,0	Training/Discovery Phase
115729	Pancreatic cancer	75	Male	5,0	823,5	Training/Discovery Phase
115787	Pancreatic cancer	75	Female	2,8	1078,0	Training/Discovery Phase
38491	Pancreatic cancer	76	Male	2,0	1284,0	Training/Discovery Phase
115653	Pancreatic cancer	76	Male	36,0	1829,0	Training/Discovery Phase
115795	Pancreatic cancer	76	Male		1913,0	Training/Discovery Phase
115798	Pancreatic cancer	76	Male		2222,0	Training/Discovery Phase
115792	Pancreatic cancer	76	Male	2,6	2537,0	Training/Discovery Phase
115715	Pancreatic cancer	76	Male	2,0	2651,0	Training/Discovery Phase
115696	Pancreatic cancer	77	Male	3,0	3336,0	Training/Discovery Phase
115727	Pancreatic cancer	77	Male		3492,0	Training/Discovery Phase
115720	Pancreatic cancer	79	Male	57,0	4867,0	Training/Discovery Phase
115757	Pancreatic cancer	82	Male	132,0	7990,0	Training/Discovery Phase
115718	Pancreatic cancer	86	Female		10500,0	Training/Discovery Phase
115699	Pancreatic cancer	88	Male	8,0	11893,0	Training/Discovery Phase
115726	Pancreatic cancer		Male	33,0	13149,0	Training/Discovery Phase
115661	Pancreatic cancer		Female	1051,0	30124,0	Training/Discovery Phase
115680	Pancreatic cancer		Female		42502,0	Training/Discovery Phase
115677	Pancreatic cancer		Female	26,0	58382,0	Training/Discovery Phase
115732	Pancreatic cancer		Male	27,0	191530,0	Training/Discovery Phase
115728	Pancreatic cancer		Female	45,0	481998,0	Training/Discovery Phase
115725	Pancreatic cancer		Female	349,0	575127,0	Training/Discovery Phase
115714	Pancreatic cancer		Male			Training/Discovery Phase
115662	Pancreatic cancer		Male			Training/Discovery Phase
115731	Pancreatic cancer		Male			Training/Discovery Phase
115761	Chronic Pancreatitis	25	Female			Test/Validation Phase
115674	Chronic Pancreatitis	28	Male			Test/Validation Phase
38566	Chronic Pancreatitis	36	Male			Test/Validation Phase
115652	Chronic Pancreatitis	39	Male			Test/Validation Phase
115745	Chronic Pancreatitis	39	Male			Test/Validation Phase
115702	Chronic Pancreatitis	43	Male			Test/Validation Phase
115683	Chronic Pancreatitis	44	Male			Test/Validation Phase
115697	Chronic Pancreatitis	45	Male			Test/Validation Phase
115751	Chronic Pancreatitis	47	Female	7,0	1,0	Test/Validation Phase
115756	Chronic Pancreatitis	47	Male	17,0	1,0	Test/Validation Phase
115640	Chronic Pancreatitis	48	Female	5,0	2,0	Test/Validation Phase
38474	Chronic Pancreatitis	49	Male	5,0	2,0	Test/Validation Phase
115679	Chronic Pancreatitis	50	Male	10,0	2,0	Test/Validation Phase
115645	Chronic Pancreatitis	50	Male	13,0	2,0	Test/Validation Phase
115759	Chronic Pancreatitis	51	Male	21,0	2,0	Test/Validation Phase
115767	Chronic Pancreatitis	52	Male	0,6	2,6	Test/Validation Phase
115685	Chronic Pancreatitis	52	Male	3,0	3,0	Test/Validation Phase
115776	Chronic Pancreatitis	54	Male	5,0	3,0	Test/Validation Phase

CE-MS Analysis ID	Diagnosis	Age	Gender	CA19-9 level	CEA level	Analytical Phase
115770	Chronic Pancreatitis	56	Male	7,0	3,0	Test/Validation Phase
115750	Chronic Pancreatitis	57	Male	14,0	3,0	Test/Validation Phase
115647	Chronic Pancreatitis	59	Male	99,0	3,0	Test/Validation Phase
115664	Chronic Pancreatitis	59	Male	13,0	4,0	Test/Validation Phase
38468	Chronic Pancreatitis	60	Male	24,0	4,0	Test/Validation Phase
115710	Chronic Pancreatitis	61	Male	36,0	4,0	Test/Validation Phase
115668	Chronic Pancreatitis	61	Male	363,0	4,0	Test/Validation Phase
115648	Chronic Pancreatitis	62	Male	19,0	5,0	Test/Validation Phase
115687	Chronic Pancreatitis	65	Male	51,0	7,0	Test/Validation Phase
115634	Chronic Pancreatitis	70	Male	41,0	8,0	Test/Validation Phase
115671	Chronic Pancreatitis	73	Male	246,0	9,0	Test/Validation Phase
115797	Chronic Pancreatitis	73	Male	140,0	112,0	Test/Validation Phase
115716	Pancreatic cancer	33	Male			Test/Validation Phase
115738	Pancreatic cancer	41	Male			Test/Validation Phase
115763	Pancreatic cancer	44	Male			Test/Validation Phase
115636	Pancreatic cancer	46	Female			Test/Validation Phase
115706	Pancreatic cancer	46	Female			Test/Validation Phase
115707	Pancreatic cancer	49	Female			Test/Validation Phase
115711	Pancreatic cancer	49	Female			Test/Validation Phase
115793	Pancreatic cancer	52	Female			Test/Validation Phase
115783	Pancreatic cancer	55	Female	25,0	7,0	Test/Validation Phase
115722	Pancreatic cancer	56	Male	4,0	8,0	Test/Validation Phase
115712	Pancreatic cancer	61	Female	1,0	21,0	Test/Validation Phase
115739	Pancreatic cancer	62	Female	1,0	33,0	Test/Validation Phase
115791	Pancreatic cancer	62	Male	3,0	36,0	Test/Validation Phase
115794	Pancreatic cancer	64	Male	1,0	60,0	Test/Validation Phase
115635	Pancreatic cancer	68	Male	1,0	106,0	Test/Validation Phase
115796	Pancreatic cancer	69	Male	6,0	108,0	Test/Validation Phase
115658	Pancreatic cancer	70	Male	2,0	162,0	Test/Validation Phase
115736	Pancreatic cancer	70	Female	3,0	199,0	Test/Validation Phase
115686	Pancreatic cancer	71	Male	0,0	275,1	Test/Validation Phase
115700	Pancreatic cancer	73	Female	36,0	291,0	Test/Validation Phase
115735	Pancreatic cancer	74	Male	2,0	552,0	Test/Validation Phase
115785	Pancreatic cancer	76	Male	7,0	638,0	Test/Validation Phase
115752	Pancreatic cancer	76	Female	17,0	3321,0	Test/Validation Phase
115801	Pancreatic cancer	76	Female	0,0	10000,0	Test/Validation Phase
115778	Pancreatic cancer	78	Male	4,4	25275,0	Test/Validation Phase
115788	Pancreatic cancer	79	Female	12,0	56771,0	Test/Validation Phase
115651	Pancreatic cancer	90	Male		120931,0	Test/Validation Phase

Supplementary Table S4. **Spreadsheet 4**. Clinical and demographical data of renal cell carcinoma cohort (n=175).

CE-MS Analysis ID	Diagnosis	Stage	Age	Gender	Analytical Phase
9481	RCC control		23	Female	Training/Discovery Phase
9468	RCC control		25	Male	Training/Discovery Phase
15036	RCC control		26	Male	Training/Discovery Phase
21685	RCC control		37	Female	Training/Discovery Phase
27124	RCC control		42	Male	Training/Discovery Phase
27235	RCC control		47	Male	Training/Discovery Phase
41357	RCC control		48	Female	Training/Discovery Phase
21719	RCC control		50	Male	Training/Discovery Phase
36678	RCC control		50	Male	Training/Discovery Phase
41305	RCC control		52	Female	Training/Discovery Phase
9506	RCC control		54	Female	Training/Discovery Phase
9308	RCC control		55	Female	Training/Discovery Phase
17115	RCC control		55	Male	Training/Discovery Phase
27228	RCC control		57	Male	Training/Discovery Phase
36625	RCC control		58	Female	Training/Discovery Phase
21623	RCC control		58	Male	Training/Discovery Phase
21692	RCC control		59	Male	Training/Discovery Phase
38731	RCC control		60	Male	Training/Discovery Phase
41420	RCC control		61	Female	Training/Discovery Phase
38735	RCC control		61	Male	Training/Discovery Phase
21704	RCC control		62	Female	Training/Discovery Phase
9278	RCC control		62	Male	Training/Discovery Phase
21690	RCC control		63	Male	Training/Discovery Phase
41413	RCC control		64	Female	Training/Discovery Phase
24132	RCC control		65	Female	Training/Discovery Phase
36586	RCC control		65	Male	Training/Discovery Phase
16159	RCC control		66	Male	Training/Discovery Phase
21817	RCC control		67	Female	Training/Discovery Phase
36666	RCC control		67	Male	Training/Discovery Phase
9306	RCC control		68	Male	Training/Discovery Phase
9698	RCC control		68	Male	Training/Discovery Phase
17116	RCC control		68	Male	Training/Discovery Phase
21632	RCC control		70	Male	Training/Discovery Phase
21709	RCC control		70	Male	Training/Discovery Phase
38712	RCC control		70	Male	Training/Discovery Phase
21672	RCC control		71	Female	Training/Discovery Phase
41437	RCC control		71	Female	Training/Discovery Phase
21631	RCC control		71	Male	Training/Discovery Phase
38695	RCC control		71	Male	Training/Discovery Phase
21633	RCC control		72	Female	Training/Discovery Phase
21630	RCC control		73	Female	Training/Discovery Phase
21698	RCC control		74	Female	Training/Discovery Phase
21712	RCC control		74	Male	Training/Discovery Phase
21815	RCC control		75	Female	Training/Discovery Phase
38719	RCC control		75	Male	Training/Discovery Phase
21629	RCC control		77	Female	Training/Discovery Phase

CE-MS Analysis ID	Diagnosis	Stage	Age	Gender	Analytical Phase
21635	RCC control		77	Male	Training/Discovery Phase
21710	RCC control		77	Male	Training/Discovery Phase
21626	RCC control		78	Female	Training/Discovery Phase
21669	RCC control		78	Male	Training/Discovery Phase
21726	RCC control		82	Female	Training/Discovery Phase
21671	RCC control		84	Male	Training/Discovery Phase
39243	RCC control			Male	Training/Discovery Phase
39274	RCC control			Male	Training/Discovery Phase
10052	RCC control				Training/Discovery Phase
10054	RCC control				Training/Discovery Phase
10055	RCC control				Training/Discovery Phase
10058	RCC control				Training/Discovery Phase
10059	RCC control				Training/Discovery Phase
10063	RCC control				Training/Discovery Phase
13875	RCC control				Training/Discovery Phase
13879	RCC control				Training/Discovery Phase
13880	RCC control				Training/Discovery Phase
13897	RCC control				Training/Discovery Phase
13898	RCC control				Training/Discovery Phase
13899	RCC control				Training/Discovery Phase
15349	RCC control				Training/Discovery Phase
19100	RCC control				Training/Discovery Phase
19157	RCC control				Training/Discovery Phase
19190	RCC control				Training/Discovery Phase
23985	RCC control				Training/Discovery Phase
24023	RCC control				Training/Discovery Phase
24032	RCC control				Training/Discovery Phase
24245	RCC control				Training/Discovery Phase
10797	RCC control			Female	Training/Discovery Phase
9449	RCC control			Male	Training/Discovery Phase
10796	RCC control			Male	Training/Discovery Phase
10051	RCC control				Training/Discovery Phase
10061	RCC control				Training/Discovery Phase
10070	RCC control				Training/Discovery Phase
10081	RCC case	pT1	41	Female	Training/Discovery Phase
18992	RCC case	pT1	41	Male	Training/Discovery Phase
18602	RCC case	pT2	41	Female	Training/Discovery Phase
19908	RCC case		42	Male	Training/Discovery Phase
18971	RCC case		44	Male	Training/Discovery Phase
19971	RCC case	pT1	46	Male	Training/Discovery Phase
10087	RCC case	pT2	46	Male	Training/Discovery Phase
18636	RCC case	pT2	48	Male	Training/Discovery Phase
13900	RCC case	pT3	49	Male	Training/Discovery Phase
18962	RCC case	pT1	50	Male	Training/Discovery Phase
15344	RCC case	pT3	51	Female	Training/Discovery Phase
18887	RCC case	pT1	52	Female	Training/Discovery Phase
18929	RCC case		53	Male	Training/Discovery Phase
18938	RCC case	pT1	55	Female	Training/Discovery Phase
18973	RCC case	pT2	57	Female	Training/Discovery Phase

CE-MS Analysis ID	Diagnosis	Stage	Age	Gender	Analytical Phase
18960	RCC case		57	Male	Training/Discovery Phase
15343	RCC case	pT1	58	Male	Training/Discovery Phase
19821	RCC case	pT2	58	Male	Training/Discovery Phase
10071	RCC case	pT1	60	Male	Training/Discovery Phase
18994	RCC case	pT3	60	Male	Training/Discovery Phase
18905	RCC case	pT1	61	Male	Training/Discovery Phase
18872	RCC case	pT2	61	Female	Training/Discovery Phase
10079	RCC case	pT1	62	Male	Training/Discovery Phase
10089	RCC case	pT2	64	Male	Training/Discovery Phase
13878	RCC case	pT1	66	Male	Training/Discovery Phase
18516	RCC case	pT2	66	Male	Training/Discovery Phase
18934	RCC case	pT3	66	Male	Training/Discovery Phase
10076	RCC case	pT1	67	Male	Training/Discovery Phase
10090	RCC case	pT3	67	Female	Training/Discovery Phase
18870	RCC case	pT3	67	Female	Training/Discovery Phase
13877	RCC case	pT3	68	Male	Training/Discovery Phase
10091	RCC case	pT1	69	Male	Training/Discovery Phase
19893	RCC case	pT1	70	Female	Training/Discovery Phase
10080	RCC case		71	Female	Training/Discovery Phase
18736	RCC case	pT1	72	Female	Training/Discovery Phase
10093	RCC case	pT3	72	Male	Training/Discovery Phase
10077	RCC case	pT3	73	Female	Training/Discovery Phase
15342	RCC case	pT3	73	Male	Training/Discovery Phase
15337	RCC case		74	Male	Training/Discovery Phase
18883	RCC case		74	Male	Training/Discovery Phase
18958	RCC case	pT1	75	Female	Training/Discovery Phase
10088	RCC case	pT3	75	Female	Training/Discovery Phase
13913	RCC case	pT1	79	Male	Training/Discovery Phase
18930	RCC case	pT1	79	Male	Training/Discovery Phase
18942	RCC case		79	Female	Training/Discovery Phase
10072	RCC case	pT3	81	Male	Training/Discovery Phase
9464	RCC control		28	Female	Test/Validation Phase
38690	RCC control		51	Male	Test/Validation Phase
10804	RCC control		52	Female	Test/Validation Phase
41379	RCC control		55	Female	Test/Validation Phase
21689	RCC control		56	Male	Test/Validation Phase
21627	RCC control		58	Male	Test/Validation Phase
38701	RCC control		59	Male	Test/Validation Phase
41331	RCC control		60	Female	Test/Validation Phase
36624	RCC control		63	Male	Test/Validation Phase
37610	RCC control		64	Female	Test/Validation Phase
21624	RCC control		64	Male	Test/Validation Phase
38700	RCC control		64	Male	Test/Validation Phase
24154	RCC control		65	Female	Test/Validation Phase
38684	RCC control		66	Male	Test/Validation Phase
21662	RCC control		67	Female	Test/Validation Phase
21663	RCC control		68	Female	Test/Validation Phase
41361	RCC control		70	Female	Test/Validation Phase
38696	RCC control		70	Male	Test/Validation Phase

CE-MS Analysis ID	Diagnosis	Stage	Age	Gender	Analytical Phase
21793	RCC control		71	Male	Test/Validation Phase
21702	RCC control		72	Female	Test/Validation Phase
21676	RCC control		75	Male	Test/Validation Phase
37876	RCC control		75	Male	Test/Validation Phase
21668	RCC control		77	Female	Test/Validation Phase
21686	RCC control		78	Male	Test/Validation Phase
38693	RCC control		80	Male	Test/Validation Phase
18723	RCC case	pT1	44	Female	Test/Validation Phase
18921	RCC case	pT3	54	Female	Test/Validation Phase
19016	RCC case	pT1	56	Female	Test/Validation Phase
18885	RCC case		58	Female	Test/Validation Phase
18919	RCC case		63	Female	Test/Validation Phase
10086	RCC case		68	Female	Test/Validation Phase
10073	RCC case	pT1	69	Female	Test/Validation Phase
18578	RCC case		74	Female	Test/Validation Phase
18543	RCC case		78	Female	Test/Validation Phase
10075	RCC case		33	Male	Test/Validation Phase
10082	RCC case		36	Male	Test/Validation Phase
18928	RCC case		44	Male	Test/Validation Phase
18963	RCC case		49	Male	Test/Validation Phase
18937	RCC case		53	Male	Test/Validation Phase
18961	RCC case		58	Male	Test/Validation Phase
10074	RCC case		62	Male	Test/Validation Phase
18886	RCC case		64	Male	Test/Validation Phase
15345	RCC case		66	Male	Test/Validation Phase
18995	RCC case		66	Male	Test/Validation Phase
13887	RCC case		71	Male	Test/Validation Phase
18579	RCC case		72	Male	Test/Validation Phase
15348	RCC case		73	Male	Test/Validation Phase
18920	RCC case		79	Male	Test/Validation Phase
19889	RCC case		79	Male	Test/Validation Phase

Abbreviations: RCC, Renal Cell Carcinoma

Supplementary Table S4. **Spreadsheet 5**. Clinical and demographical data of cholangiocarcinoma cohort (n=240).

CE-MS Analysis ID	Diagnosis	Stage	Age	Gender	Analytical Phase
38609	Benign biliary disorders		22	Female	Training/Discovery Phase
38686	Benign biliary disorders		35	Female	Training/Discovery Phase
38577	Benign biliary disorders		37	Female	Training/Discovery Phase
38562	Benign biliary disorders		38	Female	Training/Discovery Phase
65637	Benign biliary disorders		41	Female	Training/Discovery Phase
38605	Benign biliary disorders		43	Female	Training/Discovery Phase
38641	Benign biliary disorders		45	Female	Training/Discovery Phase
52204	Benign biliary disorders		45	Male	Training/Discovery Phase
38526	Benign biliary disorders		46	Female	Training/Discovery Phase
38449	Benign biliary disorders		47	Female	Training/Discovery Phase
36193	Benign biliary disorders		50	Male	Training/Discovery Phase
52273	Benign biliary disorders		51	Female	Training/Discovery Phase
36157	Benign biliary disorders		56	Male	Training/Discovery Phase
52183	Benign biliary disorders		57	Female	Training/Discovery Phase
52174	Benign biliary disorders		59	Male	Training/Discovery Phase
52175	Benign biliary disorders		60	Female	Training/Discovery Phase
52272	Benign biliary disorders		62	Male	Training/Discovery Phase
52283	Benign biliary disorders		64	Male	Training/Discovery Phase
38525	Benign biliary disorders		66	Male	Training/Discovery Phase
38530	Benign biliary disorders		66	Female	Training/Discovery Phase
38492	Benign biliary disorders		68	Male	Training/Discovery Phase
36187	Benign biliary disorders		70	Male	Training/Discovery Phase
38519	Benign biliary disorders		75	Female	Training/Discovery Phase
52269	Benign biliary disorders		76	Male	Training/Discovery Phase
38523	Benign biliary disorders		80	Female	Training/Discovery Phase
52275	Benign biliary disorders		80	Male	Training/Discovery Phase
38497	Benign biliary disorders		82	Male	Training/Discovery Phase
38580	Benign biliary disorders		83	Female	Training/Discovery Phase
99904	Benign biliary disorders		30	Female	Training/Discovery Phase
99920	Benign biliary disorders		29	Female	Training/Discovery Phase
99023	Benign biliary disorders				Training/Discovery Phase
98852	Benign biliary disorders		77	Male	Training/Discovery Phase
99839	Benign biliary disorders			Male	Training/Discovery Phase
66852	Cholangiocarcinoma		22	Male	Training/Discovery Phase
52201	Cholangiocarcinoma		26	Male	Training/Discovery Phase
99907	Cholangiocarcinoma		33	Male	Training/Discovery Phase
52520	Cholangiocarcinoma		34	Male	Training/Discovery Phase
38668	Cholangiocarcinoma		35	Female	Training/Discovery Phase
99656	Cholangiocarcinoma		40	Female	Training/Discovery Phase
51669	Cholangiocarcinoma		41	Male	Training/Discovery Phase
68536	Cholangiocarcinoma	pT2	41	Male	Training/Discovery Phase
99917	Cholangiocarcinoma	pT3	42	Female	Training/Discovery Phase
85436	Cholangiocarcinoma		43	Male	Training/Discovery Phase
52173	Cholangiocarcinoma	pT3	45	Male	Training/Discovery Phase
52573	Cholangiocarcinoma		47	Male	Training/Discovery Phase

CE-MS Analysis ID	Diagnosis	Stage	Age	Gender	Analytical Phase
52181	Cholangiocarcinoma		48	Male	Training/Discovery Phase
99607	Cholangiocarcinoma	pT2	50	Female	Training/Discovery Phase
52184	Cholangiocarcinoma		50	Male	Training/Discovery Phase
57608	Cholangiocarcinoma		50	Male	Training/Discovery Phase
38674	Cholangiocarcinoma		51	Female	Training/Discovery Phase
52523	Cholangiocarcinoma		52	Female	Training/Discovery Phase
99918	Cholangiocarcinoma	pT2	53	Male	Training/Discovery Phase
92477	Cholangiocarcinoma		54	Female	Training/Discovery Phase
99922	Cholangiocarcinoma		55	Female	Training/Discovery Phase
90566	Cholangiocarcinoma	pT2	55	Male	Training/Discovery Phase
56170	Cholangiocarcinoma	pT2	56	Female	Training/Discovery Phase
99915	Cholangiocarcinoma		56	Female	Training/Discovery Phase
98973	Cholangiocarcinoma		56	Female	Training/Discovery Phase
52588	Cholangiocarcinoma		57	Female	Training/Discovery Phase
99921	Cholangiocarcinoma		57	Male	Training/Discovery Phase
38556	Cholangiocarcinoma		58	Female	Training/Discovery Phase
44345	Cholangiocarcinoma		58	Female	Training/Discovery Phase
38473	Cholangiocarcinoma		59	Female	Training/Discovery Phase
98940	Cholangiocarcinoma	pT3	59	Male	Training/Discovery Phase
38664	Cholangiocarcinoma		60	Female	Training/Discovery Phase
115456	Cholangiocarcinoma	pT2	60	Male	Training/Discovery Phase
99900	Cholangiocarcinoma		61	Female	Training/Discovery Phase
89552	Cholangiocarcinoma		61	Male	Training/Discovery Phase
99250	Cholangiocarcinoma	pT3	62	Female	Training/Discovery Phase
38606	Cholangiocarcinoma		63	Male	Training/Discovery Phase
99704	Cholangiocarcinoma		63	Male	Training/Discovery Phase
99123	Cholangiocarcinoma	pT2	63	Male	Training/Discovery Phase
99700	Cholangiocarcinoma	pT3	64	Female	Training/Discovery Phase
38654	Cholangiocarcinoma		64	Male	Training/Discovery Phase
99846	Cholangiocarcinoma		65	Female	Training/Discovery Phase
38675	Cholangiocarcinoma		65	Male	Training/Discovery Phase
115359	Cholangiocarcinoma	pT1	65	Male	Training/Discovery Phase
99522	Cholangiocarcinoma		66	Male	Training/Discovery Phase
115354	Cholangiocarcinoma	pT2	66	Male	Training/Discovery Phase
115356	Cholangiocarcinoma	pT2	66	Male	Training/Discovery Phase
38637	Cholangiocarcinoma		67	Female	Training/Discovery Phase
52596	Cholangiocarcinoma		67	Male	Training/Discovery Phase
52517	Cholangiocarcinoma		68	Female	Training/Discovery Phase
99916	Cholangiocarcinoma	pT2	68	Female	Training/Discovery Phase
99120	Cholangiocarcinoma	pT3	68	Female	Training/Discovery Phase
41234	Cholangiocarcinoma		68	Male	Training/Discovery Phase
52689	Cholangiocarcinoma		71	Male	Training/Discovery Phase
98752	Cholangiocarcinoma	pT3	71	Male	Training/Discovery Phase
44312	Cholangiocarcinoma		72	Male	Training/Discovery Phase
99024	Cholangiocarcinoma	pT3	72	Male	Training/Discovery Phase
52574	Cholangiocarcinoma		73	Male	Training/Discovery Phase
99416	Cholangiocarcinoma	pT1	73	Male	Training/Discovery Phase
99897	Cholangiocarcinoma	pT2	73	Male	Training/Discovery Phase

CE-MS Analysis ID	Diagnosis	Stage	Age	Gender	Analytical Phase
99975	Cholangiocarcinoma	pT2	73	Male	Training/Discovery Phase
51708	Cholangiocarcinoma		74	Male	Training/Discovery Phase
41244	Cholangiocarcinoma		74	Male	Training/Discovery Phase
115357	Cholangiocarcinoma		75	Female	Training/Discovery Phase
99412	Cholangiocarcinoma		75	Male	Training/Discovery Phase
38615	Cholangiocarcinoma		76	Male	Training/Discovery Phase
99130	Cholangiocarcinoma		76	Male	Training/Discovery Phase
99360	Cholangiocarcinoma		77	Female	Training/Discovery Phase
99418	Cholangiocarcinoma	pT1	77	Male	Training/Discovery Phase
41236	Cholangiocarcinoma		78	Female	Training/Discovery Phase
115352	Cholangiocarcinoma		78	Male	Training/Discovery Phase
52592	Cholangiocarcinoma		79	Female	Training/Discovery Phase
99908	Cholangiocarcinoma	pT2	79	Female	Training/Discovery Phase
98783	Cholangiocarcinoma		79	Male	Training/Discovery Phase
52591	Cholangiocarcinoma		81	Male	Training/Discovery Phase
52577	Cholangiocarcinoma				Training/Discovery Phase
52579	Cholangiocarcinoma			Female	Training/Discovery Phase
52572	Cholangiocarcinoma			Female	Training/Discovery Phase
52600	Cholangiocarcinoma			Female	Training/Discovery Phase
52944	Cholangiocarcinoma			Male	Training/Discovery Phase
52597	Cholangiocarcinoma			Male	Training/Discovery Phase
52590	Cholangiocarcinoma			Male	Training/Discovery Phase
52554	Primary sclerosing cholangitis		20	Female	Training/Discovery Phase
38665	Primary sclerosing cholangitis		22	Male	Training/Discovery Phase
61033	Primary sclerosing cholangitis		23	Male	Training/Discovery Phase
85456	Primary sclerosing cholangitis		24	Male	Training/Discovery Phase
38671	Primary sclerosing cholangitis		26	Female	Training/Discovery Phase
73863	Primary sclerosing cholangitis		26	Male	Training/Discovery Phase
38498	Primary sclerosing cholangitis		28	Male	Training/Discovery Phase
52513	Primary sclerosing cholangitis		30	Female	Training/Discovery Phase
52553	Primary sclerosing cholangitis		31	Male	Training/Discovery Phase
52549	Primary sclerosing cholangitis		31	Male	Training/Discovery Phase
52550	Primary sclerosing cholangitis		35	Male	Training/Discovery Phase
52535	Primary sclerosing cholangitis		35		Training/Discovery Phase
52534	Primary sclerosing cholangitis		37	Male	Training/Discovery Phase
55615	Primary sclerosing cholangitis		37	Female	Training/Discovery Phase
56200	Primary sclerosing cholangitis		38	Male	Training/Discovery Phase
38558	Primary sclerosing cholangitis		39	Male	Training/Discovery Phase
80686	Primary sclerosing cholangitis		39	Male	Training/Discovery Phase
38661	Primary sclerosing cholangitis		40	Male	Training/Discovery Phase
89871	Primary sclerosing cholangitis		42	Male	Training/Discovery Phase
52522	Primary sclerosing cholangitis		42	Male	Training/Discovery Phase
52516	Primary sclerosing cholangitis		43	Male	Training/Discovery Phase
38632	Primary sclerosing cholangitis		44	Male	Training/Discovery Phase
38635	Primary sclerosing cholangitis		45	Male	Training/Discovery Phase
38502	Primary sclerosing cholangitis		46	Male	Training/Discovery Phase
55623	Primary sclerosing cholangitis		46	Male	Training/Discovery Phase
38540	Primary sclerosing cholangitis		47	Male	Training/Discovery Phase

CE-MS Analysis ID	Diagnosis	Stage	Age	Gender	Analytical Phase
52544	Primary sclerosing cholangitis		47	Male	Training/Discovery Phase
52738	Primary sclerosing cholangitis		48	Male	Training/Discovery Phase
52533	Primary sclerosing cholangitis		50	Male	Training/Discovery Phase
38662	Primary sclerosing cholangitis		50	Male	Training/Discovery Phase
52514	Primary sclerosing cholangitis		51	Male	Training/Discovery Phase
57860	Primary sclerosing cholangitis		51	Male	Training/Discovery Phase
91456	Primary sclerosing cholangitis		51	Female	Training/Discovery Phase
52540	Primary sclerosing cholangitis		52	Male	Training/Discovery Phase
38567	Primary sclerosing cholangitis		55	Male	Training/Discovery Phase
38553	Primary sclerosing cholangitis		55	Male	Training/Discovery Phase
52200	Primary sclerosing cholangitis		58	Male	Training/Discovery Phase
52541	Primary sclerosing cholangitis		58	Male	Training/Discovery Phase
38559	Primary sclerosing cholangitis		61	Male	Training/Discovery Phase
52176	Primary sclerosing cholangitis		62	Male	Training/Discovery Phase
52521	Primary sclerosing cholangitis		66	Female	Training/Discovery Phase
57862	Primary sclerosing cholangitis		83	Female	Training/Discovery Phase
109218	Primary sclerosing cholangitis		50	Female	Training/Discovery Phase
102873	Primary sclerosing cholangitis		34	Male	Training/Discovery Phase
102879	Primary sclerosing cholangitis		45	Female	Training/Discovery Phase
41240	Primary sclerosing cholangitis		39	Female	Training/Discovery Phase
41241	Primary sclerosing cholangitis		35	Female	Training/Discovery Phase
41242	Primary sclerosing cholangitis		34	Male	Training/Discovery Phase
93902	Primary sclerosing cholangitis			Male	Training/Discovery Phase
91676	Primary sclerosing cholangitis		36	Male	Training/Discovery Phase
38521	Benign biliary disorders		37	Female	Test/Validation Phase
38565	Benign biliary disorders		38	Male	Test/Validation Phase
38551	Benign biliary disorders		44	Male	Test/Validation Phase
38507	Benign biliary disorders		46	Female	Test/Validation Phase
38500	Benign biliary disorders		49	Female	Test/Validation Phase
52268	Benign biliary disorders		55	Female	Test/Validation Phase
52291	Benign biliary disorders		59	Female	Test/Validation Phase
38448	Benign biliary disorders		61	Male	Test/Validation Phase
36222	Benign biliary disorders		65	Female	Test/Validation Phase
38584	Benign biliary disorders		67	Female	Test/Validation Phase
38630	Benign biliary disorders		74	Female	Test/Validation Phase
52225	Benign biliary disorders		79	Male	Test/Validation Phase
38528	Benign biliary disorders		81	Female	Test/Validation Phase
52274	Benign biliary disorders		85	Male	Test/Validation Phase
98754	Benign biliary disorders		53	Female	Test/Validation Phase
98949	Benign biliary disorders		71	Female	Test/Validation Phase
56497	Cholangiocarcinoma		39	Female	Test/Validation Phase
99928	Cholangiocarcinoma	pT1	48	Female	Test/Validation Phase
36188	Cholangiocarcinoma		49	Female	Test/Validation Phase
99909	Cholangiocarcinoma		59	Female	Test/Validation Phase
44377	Cholangiocarcinoma		59	Female	Test/Validation Phase
98797	Cholangiocarcinoma		64	Female	Test/Validation Phase
38639	Cholangiocarcinoma		69	Female	Test/Validation Phase
90263	Cholangiocarcinoma		73	Female	Test/Validation Phase

CE-MS Analysis ID	Diagnosis	Stage	Age	Gender	Analytical Phase
99905	Cholangiocarcinoma		77	Female	Test/Validation Phase
99901	Cholangiocarcinoma		82	Female	Test/Validation Phase
99124	Cholangiocarcinoma		83	Female	Test/Validation Phase
52180	Cholangiocarcinoma		30	Male	Test/Validation Phase
51527	Cholangiocarcinoma		43	Male	Test/Validation Phase
38653	Cholangiocarcinoma		46	Male	Test/Validation Phase
36202	Cholangiocarcinoma	pT2	51	Male	Test/Validation Phase
99702	Cholangiocarcinoma	pT2	51	Male	Test/Validation Phase
99344	Cholangiocarcinoma	pT2	54	Male	Test/Validation Phase
38688	Cholangiocarcinoma		55	Male	Test/Validation Phase
44281	Cholangiocarcinoma		55	Male	Test/Validation Phase
52589	Cholangiocarcinoma		58	Male	Test/Validation Phase
99129	Cholangiocarcinoma	pT3	58	Male	Test/Validation Phase
51734	Cholangiocarcinoma		59	Male	Test/Validation Phase
93937	Cholangiocarcinoma	pT2	60	Male	Test/Validation Phase
99903	Cholangiocarcinoma	pT1	62	Male	Test/Validation Phase
99605	Cholangiocarcinoma		62	Male	Test/Validation Phase
38617	Cholangiocarcinoma		63	Male	Test/Validation Phase
38658	Cholangiocarcinoma		64	Male	Test/Validation Phase
52185	Cholangiocarcinoma		67	Male	Test/Validation Phase
99339	Cholangiocarcinoma	pT2	69	Male	Test/Validation Phase
99220	Cholangiocarcinoma		73	Male	Test/Validation Phase
99652	Cholangiocarcinoma	pT1	74	Male	Test/Validation Phase
38670	Cholangiocarcinoma		78	Male	Test/Validation Phase
99561	Cholangiocarcinoma		78	Male	Test/Validation Phase
44310	Cholangiocarcinoma		79	Male	Test/Validation Phase
99029	Cholangiocarcinoma	pT2	79	Male	Test/Validation Phase
52739	Cholangiocarcinoma		86	Male	Test/Validation Phase
52538	Primary sclerosing cholangitis		22	Male	Test/Validation Phase
76574	Primary sclerosing cholangitis		25	Male	Test/Validation Phase
68439	Primary sclerosing cholangitis		30	Male	Test/Validation Phase
55295	Primary sclerosing cholangitis		33	Male	Test/Validation Phase
76142	Primary sclerosing cholangitis		36	Male	Test/Validation Phase
52285	Primary sclerosing cholangitis		38	Male	Test/Validation Phase
52557	Primary sclerosing cholangitis		39	Male	Test/Validation Phase
58613	Primary sclerosing cholangitis		42	Male	Test/Validation Phase
53805	Primary sclerosing cholangitis		44	Male	Test/Validation Phase
52559	Primary sclerosing cholangitis		46	Male	Test/Validation Phase
52519	Primary sclerosing cholangitis		47	Male	Test/Validation Phase
65499	Primary sclerosing cholangitis		49	Male	Test/Validation Phase
38669	Primary sclerosing cholangitis		51	Male	Test/Validation Phase
91164	Primary sclerosing cholangitis		51	Male	Test/Validation Phase
76588	Primary sclerosing cholangitis		52	Female	Test/Validation Phase
52551	Primary sclerosing cholangitis		56	Male	Test/Validation Phase
38499	Primary sclerosing cholangitis		60	Female	Test/Validation Phase
38524	Primary sclerosing cholangitis		64	Female	Test/Validation Phase
109192	Primary sclerosing cholangitis		37	Male	Test/Validation Phase
102874	Primary sclerosing cholangitis		47	Male	Test/Validation Phase

CE-MS Analysis ID	Diagnosis	Stage	Age	Gender	Analytical Phase
41243	Primary sclerosing cholangitis		46	Male	Test/Validation Phase
74718	Primary sclerosing cholangitis		45	Male	Test/Validation Phase
93813	Primary sclerosing cholangitis		31	Female	Test/Validation Phase

Supplementary Table S4. **Spreadsheet 6**. Characterization of control set of health individuals (n=331).

CE-MS Analysis ID	Diagnosis	Age	Gender	Analytical Phase
22028	Healthy Individual	19	Female	Training/Discovery Phase
64130	Healthy Individual	21	Male	Training/Discovery Phase
9482	Healthy Individual	23	Female	Training/Discovery Phase
9498	Healthy Individual	24	Female	Training/Discovery Phase
64051	Healthy Individual	27	Male	Training/Discovery Phase
63749	Healthy Individual	28	Male	Training/Discovery Phase
63860	Healthy Individual	29	Male	Training/Discovery Phase
20752	Healthy Individual	30	Female	Training/Discovery Phase
63841	Healthy Individual	31	Female	Training/Discovery Phase
87755	Healthy Individual	31	Female	Training/Discovery Phase
9407	Healthy Individual	33	Female	Training/Discovery Phase
63696	Healthy Individual	33	Female	Training/Discovery Phase
87915	Healthy Individual	34	Male	Training/Discovery Phase
9488	Healthy Individual	35	Female	Training/Discovery Phase
87788	Healthy Individual	36	Female	Training/Discovery Phase
63581	Healthy Individual	37	Female	Training/Discovery Phase
16132	Healthy Individual	38	Male	Training/Discovery Phase
63976	Healthy Individual	38	Male	Training/Discovery Phase
57789	Healthy Individual	39	Female	Training/Discovery Phase
88066	Healthy Individual	40	Female	Training/Discovery Phase
39289	Healthy Individual	41	Male	Training/Discovery Phase
63946	Healthy Individual	41	Male	Training/Discovery Phase
39322	Healthy Individual	42	Male	Training/Discovery Phase
39247	Healthy Individual	43	Male	Training/Discovery Phase
39281	Healthy Individual	43	Male	Training/Discovery Phase
63530	Healthy Individual	43	Male	Training/Discovery Phase
39249	Healthy Individual	44	Male	Training/Discovery Phase
39244	Healthy Individual	45	Female	Training/Discovery Phase
63598	Healthy Individual	45	Female	Training/Discovery Phase
39305	Healthy Individual	45	Male	Training/Discovery Phase
37125	Healthy Individual	46	Female	Training/Discovery Phase
39302	Healthy Individual	47	Female	Training/Discovery Phase
63951	Healthy Individual	47	Male	Training/Discovery Phase
39313	Healthy Individual	48	Male	Training/Discovery Phase
39270	Healthy Individual	49	Male	Training/Discovery Phase
41754	Healthy Individual	50	Male	Training/Discovery Phase
39318	Healthy Individual	51	Female	Training/Discovery Phase
87924	Healthy Individual	51	Male	Training/Discovery Phase
27120	Healthy Individual	52	Female	Training/Discovery Phase
63802	Healthy Individual	52	Female	Training/Discovery Phase
63836	Healthy Individual	53	Female	Training/Discovery Phase
39293	Healthy Individual	54	Female	Training/Discovery Phase
63814	Healthy Individual	54	Male	Training/Discovery Phase
63992	Healthy Individual	54	Male	Training/Discovery Phase
39246	Healthy Individual	55	Female	Training/Discovery Phase
39304	Healthy Individual	55	Female	Training/Discovery Phase

CE-MS Analysis ID	Diagnosis	Age	Gender	Analytical Phase
48824	Healthy Individual	55	Female	Training/Discovery Phase
63832	Healthy Individual	55	Female	Training/Discovery Phase
88059	Healthy Individual	55	Male	Training/Discovery Phase
88096	Healthy Individual	55	Male	Training/Discovery Phase
39256	Healthy Individual	56	Female	Training/Discovery Phase
39324	Healthy Individual	56	Female	Training/Discovery Phase
63713	Healthy Individual	56	Female	Training/Discovery Phase
88060	Healthy Individual	56	Female	Training/Discovery Phase
16380	Healthy Individual	56	Male	Training/Discovery Phase
16160	Healthy Individual	56	Male	Training/Discovery Phase
39294	Healthy Individual	56	Male	Training/Discovery Phase
64049	Healthy Individual	56	Male	Training/Discovery Phase
39258	Healthy Individual	57	Female	Training/Discovery Phase
39300	Healthy Individual	57	Female	Training/Discovery Phase
39337	Healthy Individual	57	Female	Training/Discovery Phase
48830	Healthy Individual	57	Female	Training/Discovery Phase
48838	Healthy Individual	57	Female	Training/Discovery Phase
49366	Healthy Individual	57	Female	Training/Discovery Phase
49391	Healthy Individual	57	Female	Training/Discovery Phase
88065	Healthy Individual	57	Female	Training/Discovery Phase
37799	Healthy Individual	57	Male	Training/Discovery Phase
9336	Healthy Individual	57	Male	Training/Discovery Phase
39278	Healthy Individual	57	Male	Training/Discovery Phase
37806	Healthy Individual	58	Female	Training/Discovery Phase
39279	Healthy Individual	58	Female	Training/Discovery Phase
21627	Healthy Individual	58	Male	Training/Discovery Phase
17128	Healthy Individual	58	Male	Training/Discovery Phase
63714	Healthy Individual	58	Male	Training/Discovery Phase
64040	Healthy Individual	58	Male	Training/Discovery Phase
39273	Healthy Individual	59	Female	Training/Discovery Phase
39306	Healthy Individual	59	Female	Training/Discovery Phase
49242	Healthy Individual	59	Female	Training/Discovery Phase
63584	Healthy Individual	59	Female	Training/Discovery Phase
37652	Healthy Individual	59	Male	Training/Discovery Phase
9402	Healthy Individual	59	Male	Training/Discovery Phase
63811	Healthy Individual	59	Male	Training/Discovery Phase
87918	Healthy Individual	60	Male	Training/Discovery Phase
39349	Healthy Individual	60	Male	Training/Discovery Phase
27290	Healthy Individual	61	Female	Training/Discovery Phase
88064	Healthy Individual	61	Female	Training/Discovery Phase
63817	Healthy Individual	61	Male	Training/Discovery Phase
16395	Healthy Individual	62	Female	Training/Discovery Phase
63722	Healthy Individual	62	Female	Training/Discovery Phase
9331	Healthy Individual	62	Male	Training/Discovery Phase
87902	Healthy Individual	62	Male	Training/Discovery Phase
39469	Healthy Individual	62	Male	Training/Discovery Phase
69857	Healthy Individual	62	Male	Training/Discovery Phase
9409	Healthy Individual	63	Female	Training/Discovery Phase

CE-MS Analysis ID	Diagnosis	Age	Gender	Analytical Phase
17117	Healthy Individual	63	Male	Training/Discovery Phase
41755	Healthy Individual	63	Male	Training/Discovery Phase
37796	Healthy Individual	64	Female	Training/Discovery Phase
63830	Healthy Individual	64	Female	Training/Discovery Phase
37617	Healthy Individual	64	Male	Training/Discovery Phase
9328	Healthy Individual	64	Male	Training/Discovery Phase
24154	Healthy Individual	65	Female	Training/Discovery Phase
37640	Healthy Individual	65	Female	Training/Discovery Phase
64119	Healthy Individual	65	Female	Training/Discovery Phase
37558	Healthy Individual	65	Male	Training/Discovery Phase
37606	Healthy Individual	65	Male	Training/Discovery Phase
37611	Healthy Individual	65	Male	Training/Discovery Phase
37585	Healthy Individual	66	Female	Training/Discovery Phase
37591	Healthy Individual	66	Female	Training/Discovery Phase
37615	Healthy Individual	66	Female	Training/Discovery Phase
37624	Healthy Individual	66	Female	Training/Discovery Phase
21662	Healthy Individual	67	Female	Training/Discovery Phase
37573	Healthy Individual	67	Female	Training/Discovery Phase
37645	Healthy Individual	67	Male	Training/Discovery Phase
37646	Healthy Individual	67	Male	Training/Discovery Phase
21663	Healthy Individual	68	Female	Training/Discovery Phase
16362	Healthy Individual	68	Male	Training/Discovery Phase
37575	Healthy Individual	69	Female	Training/Discovery Phase
37554	Healthy Individual	69	Male	Training/Discovery Phase
37653	Healthy Individual	69	Male	Training/Discovery Phase
16351	Healthy Individual	69	Male	Training/Discovery Phase
87927	Healthy Individual	69	Male	Training/Discovery Phase
37584	Healthy Individual	70	Female	Training/Discovery Phase
37702	Healthy Individual	70	Female	Training/Discovery Phase
37593	Healthy Individual	70	Male	Training/Discovery Phase
16363	Healthy Individual	70	Male	Training/Discovery Phase
37570	Healthy Individual	71	Female	Training/Discovery Phase
37587	Healthy Individual	71	Female	Training/Discovery Phase
37686	Healthy Individual	71	Female	Training/Discovery Phase
37934	Healthy Individual	71	Female	Training/Discovery Phase
63998	Healthy Individual	71	Female	Training/Discovery Phase
21678	Healthy Individual	71	Male	Training/Discovery Phase
37933	Healthy Individual	71	Male	Training/Discovery Phase
37601	Healthy Individual	72	Female	Training/Discovery Phase
37620	Healthy Individual	72	Male	Training/Discovery Phase
37677	Healthy Individual	72	Male	Training/Discovery Phase
37618	Healthy Individual	73	Female	Training/Discovery Phase
17346	Healthy Individual	73	Female	Training/Discovery Phase
37651	Healthy Individual	73	Male	Training/Discovery Phase
37553	Healthy Individual	74	Female	Training/Discovery Phase
37556	Healthy Individual	74	Female	Training/Discovery Phase
37637	Healthy Individual	74	Female	Training/Discovery Phase
37555	Healthy Individual	75	Female	Training/Discovery Phase

CE-MS Analysis ID	Diagnosis	Age	Gender	Analytical Phase
37643	Healthy Individual	75	Female	Training/Discovery Phase
37650	Healthy Individual	75	Male	Training/Discovery Phase
37595	Healthy Individual	76	Female	Training/Discovery Phase
37557	Healthy Individual	76	Male	Training/Discovery Phase
21668	Healthy Individual	77	Female	Training/Discovery Phase
63995	Healthy Individual	77	Female	Training/Discovery Phase
37559	Healthy Individual	78	Female	Training/Discovery Phase
37572	Healthy Individual	78	Female	Training/Discovery Phase
21686	Healthy Individual	78	Male	Training/Discovery Phase
37613	Healthy Individual	78	Male	Training/Discovery Phase
37743	Healthy Individual	78	Male	Training/Discovery Phase
37681	Healthy Individual	79	Female	Training/Discovery Phase
37596	Healthy Individual	79	Male	Training/Discovery Phase
37562	Healthy Individual	80	Female	Training/Discovery Phase
37588	Healthy Individual	80	Male	Training/Discovery Phase
37795	Healthy Individual	81	Female	Training/Discovery Phase
37552	Healthy Individual	81	Male	Training/Discovery Phase
37614	Healthy Individual	81	Male	Training/Discovery Phase
37642	Healthy Individual	81	Male	Training/Discovery Phase
37592	Healthy Individual	82	Female	Training/Discovery Phase
21795	Healthy Individual	83	Female	Training/Discovery Phase
37571	Healthy Individual	84	Female	Training/Discovery Phase
37794	Healthy Individual	84	Female	Training/Discovery Phase
37896	Healthy Individual	84	Male	Training/Discovery Phase
37886	Healthy Individual	86	Female	Training/Discovery Phase
9505	Healthy Individual	22	Female	Test/Validation Phase
63933	Healthy Individual	25	Male	Test/Validation Phase
20748	Healthy Individual	28	Male	Test/Validation Phase
69853	Healthy Individual	30	Male	Test/Validation Phase
37134	Healthy Individual	32	Male	Test/Validation Phase
63960	Healthy Individual	34	Male	Test/Validation Phase
63726	Healthy Individual	36	Male	Test/Validation Phase
9503	Healthy Individual	37	Female	Test/Validation Phase
63683	Healthy Individual	39	Male	Test/Validation Phase
39260	Healthy Individual	40	Male	Test/Validation Phase
64239	Healthy Individual	42	Male	Test/Validation Phase
39271	Healthy Individual	43	Male	Test/Validation Phase
64038	Healthy Individual	44	Female	Test/Validation Phase
39275	Healthy Individual	45	Male	Test/Validation Phase
63676	Healthy Individual	45	Male	Test/Validation Phase
63718	Healthy Individual	46	Female	Test/Validation Phase
63796	Healthy Individual	47	Female	Test/Validation Phase
39290	Healthy Individual	49	Male	Test/Validation Phase
64116	Healthy Individual	50	Female	Test/Validation Phase
39282	Healthy Individual	51	Female	Test/Validation Phase
63728	Healthy Individual	52	Female	Test/Validation Phase
64053	Healthy Individual	53	Female	Test/Validation Phase
39268	Healthy Individual	54	Female	Test/Validation Phase

CE-MS Analysis ID	Diagnosis	Age	Gender	Analytical Phase
9308	Healthy Individual	55	Female	Test/Validation Phase
48841	Healthy Individual	55	Female	Test/Validation Phase
63894	Healthy Individual	55	Female	Test/Validation Phase
39304	Healthy Individual	55	Female	Test/Validation Phase
37854	Healthy Individual	56	Female	Test/Validation Phase
63913	Healthy Individual	56	Female	Test/Validation Phase
88067	Healthy Individual	56	Female	Test/Validation Phase
39324	Healthy Individual	56	Female	Test/Validation Phase
39294	Healthy Individual	56	Male	Test/Validation Phase
39284	Healthy Individual	57	Female	Test/Validation Phase
39258	Healthy Individual	57	Female	Test/Validation Phase
39337	Healthy Individual	57	Female	Test/Validation Phase
37902	Healthy Individual	57	Male	Test/Validation Phase
27125	Healthy Individual	57	Male	Test/Validation Phase
39321	Healthy Individual	57	Male	Test/Validation Phase
21674	Healthy Individual	58	Female	Test/Validation Phase
39320	Healthy Individual	58	Female	Test/Validation Phase
39306	Healthy Individual	59	Female	Test/Validation Phase
49242	Healthy Individual	59	Female	Test/Validation Phase
63905	Healthy Individual	59	Male	Test/Validation Phase
39283	Healthy Individual	60	Male	Test/Validation Phase
37798	Healthy Individual	61	Female	Test/Validation Phase
63952	Healthy Individual	61	Female	Test/Validation Phase
37329	Healthy Individual	62	Male	Test/Validation Phase
39274	Healthy Individual	62	Male	Test/Validation Phase
63970	Healthy Individual	63	Female	Test/Validation Phase
37793	Healthy Individual	63	Male	Test/Validation Phase
16142	Healthy Individual	63	Male	Test/Validation Phase
37583	Healthy Individual	65	Male	Test/Validation Phase
37771	Healthy Individual	65	Male	Test/Validation Phase
87928	Healthy Individual	65	Male	Test/Validation Phase
37568	Healthy Individual	66	Female	Test/Validation Phase
63949	Healthy Individual	66	Female	Test/Validation Phase
63732	Healthy Individual	67	Female	Test/Validation Phase
37551	Healthy Individual	67	Male	Test/Validation Phase
37582	Healthy Individual	68	Female	Test/Validation Phase
26849	Healthy Individual	68	Male	Test/Validation Phase
37904	Healthy Individual	69	Female	Test/Validation Phase
16352	Healthy Individual	69	Male	Test/Validation Phase
37792	Healthy Individual	70	Female	Test/Validation Phase
37576	Healthy Individual	70	Male	Test/Validation Phase
87930	Healthy Individual	70	Male	Test/Validation Phase
21672	Healthy Individual	71	Female	Test/Validation Phase
37616	Healthy Individual	71	Female	Test/Validation Phase
17118	Healthy Individual	71	Female	Test/Validation Phase
37567	Healthy Individual	71	Male	Test/Validation Phase
37921	Healthy Individual	72	Female	Test/Validation Phase
37679	Healthy Individual	72	Male	Test/Validation Phase

CE-MS Analysis ID	Diagnosis	Age	Gender	Analytical Phase
63794	Healthy Individual	72	Male	Test/Validation Phase
37882	Healthy Individual	74	Female	Test/Validation Phase
21712	Healthy Individual	74	Male	Test/Validation Phase
37636	Healthy Individual	75	Male	Test/Validation Phase
37594	Healthy Individual	76	Female	Test/Validation Phase
37589	Healthy Individual	76	Male	Test/Validation Phase
21629	Healthy Individual	77	Female	Test/Validation Phase
37602	Healthy Individual	77	Female	Test/Validation Phase
21626	Healthy Individual	78	Female	Test/Validation Phase
37914	Healthy Individual	78	Female	Test/Validation Phase
37901	Healthy Individual	79	Male	Test/Validation Phase
37574	Healthy Individual	80	Male	Test/Validation Phase
9403	Healthy Individual	81	Female	Test/Validation Phase
37590	Healthy Individual	81	Male	Test/Validation Phase
21726	Healthy Individual	82	Female	Test/Validation Phase
37641	Healthy Individual	83	Female	Test/Validation Phase
37887	Healthy Individual	84	Female	Test/Validation Phase
20706	Healthy Individual	86	Male	Test/Validation Phase
69673	Individual with smoking history	19	Female	Training/Discovery Phase
9447	Individual with smoking history	20	Female	Training/Discovery Phase
9475	Individual with smoking history	20	Male	Training/Discovery Phase
9490	Individual with smoking history	22	Female	Training/Discovery Phase
10808	Individual with smoking history	22	Female	Training/Discovery Phase
10809	Individual with smoking history	23	Female	Training/Discovery Phase
87793	Individual with smoking history	23	Female	Training/Discovery Phase
9474	Individual with smoking history	24	Female	Training/Discovery Phase
10814	Individual with smoking history	25	Female	Training/Discovery Phase
10813	Individual with smoking history	25	Male	Training/Discovery Phase
87762	Individual with smoking history	25	Male	Training/Discovery Phase
9485	Individual with smoking history	26	Male	Training/Discovery Phase
10817	Individual with smoking history	27	Female	Training/Discovery Phase
69667	Individual with smoking history	27	Female	Training/Discovery Phase
10810	Individual with smoking history	29	Female	Training/Discovery Phase
69678	Individual with smoking history	29	Female	Training/Discovery Phase
10812	Individual with smoking history	31	Female	Training/Discovery Phase
9491	Individual with smoking history	33	Female	Training/Discovery Phase
87949	Individual with smoking history	33	Male	Training/Discovery Phase
9477	Individual with smoking history	34	Female	Training/Discovery Phase
9469	Individual with smoking history	38	Female	Training/Discovery Phase
36499	Individual with smoking history	40	Female	Training/Discovery Phase
50511	Individual with smoking history	44	Female	Training/Discovery Phase
50574	Individual with smoking history	44	Female	Training/Discovery Phase
9479	Individual with smoking history	45	Male	Training/Discovery Phase
37339	Individual with smoking history	46	Female	Training/Discovery Phase
37872	Individual with smoking history	47	Male	Training/Discovery Phase
50372	Individual with smoking history	50	Male	Training/Discovery Phase
50378	Individual with smoking history	51	Male	Training/Discovery Phase
50521	Individual with smoking history	51	Female	Training/Discovery Phase

CE-MS Analysis ID	Diagnosis	Age	Gender	Analytical Phase
87938	Individual with smoking history	52	Female	Training/Discovery Phase
36675	Individual with smoking history	52	Male	Training/Discovery Phase
73188	Individual with smoking history	52	Male	Training/Discovery Phase
50379	Individual with smoking history	52	Male	Training/Discovery Phase
9451	Individual with smoking history	53	Female	Training/Discovery Phase
50502	Individual with smoking history	54	Female	Training/Discovery Phase
36627	Individual with smoking history	54	Female	Training/Discovery Phase
36495	Individual with smoking history	55	Male	Training/Discovery Phase
50528	Individual with smoking history	56	Female	Training/Discovery Phase
36626	Individual with smoking history	56	Male	Training/Discovery Phase
37505	Individual with smoking history	57	Male	Training/Discovery Phase
50564	Individual with smoking history	58	Female	Training/Discovery Phase
50358	Individual with smoking history	58	Male	Training/Discovery Phase
55765	Individual with smoking history	58	Male	Training/Discovery Phase
50611	Individual with smoking history	62	Male	Training/Discovery Phase
50532	Individual with smoking history	63	Male	Training/Discovery Phase
37835	Individual with smoking history	64	Male	Training/Discovery Phase
73208	Individual with smoking history	65	Male	Training/Discovery Phase
36503	Individual with smoking history	66	Male	Training/Discovery Phase
73228	Individual with smoking history	68	Female	Training/Discovery Phase
37501	Individual with smoking history	72	Male	Training/Discovery Phase
9472	Individual with smoking history	20	Female	Test/Validation Phase
10811	Individual with smoking history	22	Female	Test/Validation Phase
69695	Individual with smoking history	23	Female	Test/Validation Phase
9499	Individual with smoking history	24	Female	Test/Validation Phase
9468	Individual with smoking history	25	Male	Test/Validation Phase
9473	Individual with smoking history	25	Male	Test/Validation Phase
9483	Individual with smoking history	27	Female	Test/Validation Phase
9489	Individual with smoking history	33	Female	Test/Validation Phase
10819	Individual with smoking history	36	Female	Test/Validation Phase
55760	Individual with smoking history	42	Male	Test/Validation Phase
55761	Individual with smoking history	45	Male	Test/Validation Phase
37865	Individual with smoking history	46	Female	Test/Validation Phase
36678	Individual with smoking history	50	Male	Test/Validation Phase
9506	Individual with smoking history	54	Female	Test/Validation Phase
37085	Individual with smoking history	54	Female	Test/Validation Phase
73193	Individual with smoking history	56	Male	Test/Validation Phase
9443	Individual with smoking history	57	Female	Test/Validation Phase
10807	Individual with smoking history	60	Male	Test/Validation Phase
37745	Individual with smoking history	62	Male	Test/Validation Phase
73199	Individual with smoking history	63	Male	Test/Validation Phase
55782	Individual with smoking history	64	Male	Test/Validation Phase
55840	Individual with smoking history	67	Female	Test/Validation Phase
37836	Individual with smoking history	68	Male	Test/Validation Phase
36622	Individual with smoking history	72	Female	Test/Validation Phase

Supplementary Table S4. **Spreadsheet 7**. Characterisation of control set of patients with inflammatory diseases (n=199).

CE-MS Analysis ID	Diagnosis	Age	Gender	Analytical Phase
36410	Arteriosclerosis	51	Male	Training/Discovery Phase
37840	Arteriosclerosis	74	Female	Training/Discovery Phase
49494	IgA nephropathy	16	Male	Training/Discovery Phase
49534	IgA nephropathy	16	Male	Training/Discovery Phase
22775	IgA nephropathy	17	Male	Training/Discovery Phase
49484	IgA nephropathy	19	Female	Training/Discovery Phase
49492	IgA nephropathy	19	Female	Training/Discovery Phase
61966	IgA nephropathy	19	Male	Training/Discovery Phase
49518	IgA nephropathy	22	Male	Training/Discovery Phase
61973	IgA nephropathy	22	Male	Training/Discovery Phase
49480	IgA nephropathy	23	Male	Training/Discovery Phase
20199	IgA nephropathy	24	Female	Training/Discovery Phase
27876	IgA nephropathy	24	Female	Training/Discovery Phase
49546	IgA nephropathy	24	Female	Training/Discovery Phase
27943	IgA nephropathy	25	Female	Training/Discovery Phase
20059	IgA nephropathy	25	Male	Training/Discovery Phase
22774	IgA nephropathy	26	Female	Training/Discovery Phase
49481	IgA nephropathy	26	Male	Training/Discovery Phase
49516	IgA nephropathy	27	Male	Training/Discovery Phase
22780	IgA nephropathy	28	Female	Training/Discovery Phase
49564	IgA nephropathy	29	Male	Training/Discovery Phase
27705	IgA nephropathy	30	Female	Training/Discovery Phase
49532	IgA nephropathy	30	Female	Training/Discovery Phase
20071	IgA nephropathy	30	Male	Training/Discovery Phase
49487	IgA nephropathy	30	Male	Training/Discovery Phase
22778	IgA nephropathy	31	Female	Training/Discovery Phase
49533	IgA nephropathy	33	Female	Training/Discovery Phase
27848	IgA nephropathy	33	Male	Training/Discovery Phase
49495	IgA nephropathy	34	Female	Training/Discovery Phase
49548	IgA nephropathy	34	Male	Training/Discovery Phase
61970	IgA nephropathy	35	Male	Training/Discovery Phase
22928	IgA nephropathy	36	Male	Training/Discovery Phase
23122	IgA nephropathy	36	Male	Training/Discovery Phase
27911	IgA nephropathy	38	Female	Training/Discovery Phase
61967	IgA nephropathy	38	Female	Training/Discovery Phase
27706	IgA nephropathy	41	Female	Training/Discovery Phase
49531	IgA nephropathy	42	Male	Training/Discovery Phase
49493	IgA nephropathy	44	Female	Training/Discovery Phase
22772	IgA nephropathy	45	Female	Training/Discovery Phase
22805	IgA nephropathy	45	Male	Training/Discovery Phase
27802	IgA nephropathy	45	Male	Training/Discovery Phase
49529	IgA nephropathy	45	Male	Training/Discovery Phase
27804	IgA nephropathy	48	Male	Training/Discovery Phase
27778	IgA nephropathy	51	Male	Training/Discovery Phase
27707	IgA nephropathy	52	Male	Training/Discovery Phase
22802	IgA nephropathy	55	Female	Training/Discovery Phase
22929	IgA nephropathy	55	Female	Training/Discovery Phase
23123	IgA nephropathy	55	Female	Training/Discovery Phase
27917	IgA nephropathy	56	Female	Training/Discovery Phase
49485	IgA nephropathy	57	Female	Training/Discovery Phase
27767	IgA nephropathy	57	Male	Training/Discovery Phase
27768	IgA nephropathy	57	Male	Training/Discovery Phase
23187	IgA nephropathy	63	Male	Training/Discovery Phase
63634	Rheumatoid arthritis	26	Female	Training/Discovery Phase

CE-MS Analysis ID	Diagnosis	Age	Gender	Analytical Phase
63638	Rheumatoid arthritis	33	Male	Training/Discovery Phase
63624	Rheumatoid arthritis	36	Female	Training/Discovery Phase
63651	Rheumatoid arthritis	37	Male	Training/Discovery Phase
63625	Rheumatoid arthritis	38	Female	Training/Discovery Phase
63671	Rheumatoid arthritis	38	Female	Training/Discovery Phase
63664	Rheumatoid arthritis	40	Female	Training/Discovery Phase
63636	Rheumatoid arthritis	45	Female	Training/Discovery Phase
63666	Rheumatoid arthritis	48	Female	Training/Discovery Phase
63667	Rheumatoid arthritis	48	Female	Training/Discovery Phase
63626	Rheumatoid arthritis	53	Female	Training/Discovery Phase
63640	Rheumatoid arthritis	54	Female	Training/Discovery Phase
63663	Rheumatoid arthritis	54	Female	Training/Discovery Phase
63669	Rheumatoid arthritis	55	Female	Training/Discovery Phase
63635	Rheumatoid arthritis	57	Female	Training/Discovery Phase
63670	Rheumatoid arthritis	57	Male	Training/Discovery Phase
63658	Rheumatoid arthritis	58	Female	Training/Discovery Phase
63632	Rheumatoid arthritis	59	Female	Training/Discovery Phase
63650	Rheumatoid arthritis	59	Female	Training/Discovery Phase
63662	Rheumatoid arthritis	61	Male	Training/Discovery Phase
63665	Rheumatoid arthritis	63	Female	Training/Discovery Phase
63660	Rheumatoid arthritis	64	Female	Training/Discovery Phase
63647	Rheumatoid arthritis	65	Female	Training/Discovery Phase
63659	Rheumatoid arthritis	66	Female	Training/Discovery Phase
63628	Rheumatoid arthritis	66	Male	Training/Discovery Phase
63639	Rheumatoid arthritis	67	Female	Training/Discovery Phase
63627	Rheumatoid arthritis	68	Female	Training/Discovery Phase
63656	Rheumatoid arthritis	69	Male	Training/Discovery Phase
63641	Rheumatoid arthritis	70	Male	Training/Discovery Phase
63631	Rheumatoid arthritis	71	Male	Training/Discovery Phase
63646	Rheumatoid arthritis	71	Male	Training/Discovery Phase
63642	Rheumatoid arthritis	74	Female	Training/Discovery Phase
63649	Rheumatoid arthritis	74	Male	Training/Discovery Phase
63654	Rheumatoid arthritis	88	Female	Training/Discovery Phase
28845	Systemic lupus erythematosus	20	Male	Training/Discovery Phase
31909	Systemic lupus erythematosus	22	Female	Training/Discovery Phase
56650	Systemic lupus erythematosus	22	Female	Training/Discovery Phase
28906	Systemic lupus erythematosus	23	Male	Training/Discovery Phase
30311	Systemic lupus erythematosus	25	Female	Training/Discovery Phase
56672	Systemic lupus erythematosus	26	Female	Training/Discovery Phase
31879	Systemic lupus erythematosus	28	Female	Training/Discovery Phase
31892	Systemic lupus erythematosus	30	Female	Training/Discovery Phase
56654	Systemic lupus erythematosus	30	Female	Training/Discovery Phase
31740	Systemic lupus erythematosus	32	Female	Training/Discovery Phase
28904	Systemic lupus erythematosus	35	Female	Training/Discovery Phase
31677	Systemic lupus erythematosus	35	Female	Training/Discovery Phase
56657	Systemic lupus erythematosus	35	Female	Training/Discovery Phase
28908	Systemic lupus erythematosus	36	Female	Training/Discovery Phase
28919	Systemic lupus erythematosus	38	Male	Training/Discovery Phase
31388	Systemic lupus erythematosus	39	Female	Training/Discovery Phase
56677	Systemic lupus erythematosus	39	Female	Training/Discovery Phase
31935	Systemic lupus erythematosus	44	Female	Training/Discovery Phase
56683	Systemic lupus erythematosus	44	Female	Training/Discovery Phase
29290	Systemic lupus erythematosus	45	Female	Training/Discovery Phase
28846	Systemic lupus erythematosus	48	Male	Training/Discovery Phase
28854	Systemic lupus erythematosus	50	Female	Training/Discovery Phase
31380	Systemic lupus erythematosus	50	Female	Training/Discovery Phase
56653	Systemic lupus erythematosus	50	Female	Training/Discovery Phase

CE-MS Analysis ID	Diagnosis	Age	Gender	Analytical Phase
56670	Systemic lupus erythematosus	50	Female	Training/Discovery Phase
28905	Systemic lupus erythematosus	53	Male	Training/Discovery Phase
31378	Systemic lupus erythematosus	53	Male	Training/Discovery Phase
56662	Systemic lupus erythematosus	53	Male	Training/Discovery Phase
56690	Systemic lupus erythematosus	53	Male	Training/Discovery Phase
28822	Systemic lupus erythematosus	57	Male	Training/Discovery Phase
31741	Systemic lupus erythematosus	61	Female	Training/Discovery Phase
28907	Systemic lupus erythematosus	66	Female	Training/Discovery Phase
61747	Vasculitis	62	Female	Training/Discovery Phase
62100	Vasculitis	62	Female	Training/Discovery Phase
61832	Vasculitis	63	Female	Training/Discovery Phase
62114	Vasculitis	63	Female	Training/Discovery Phase
61750	Vasculitis	64	Male	Training/Discovery Phase
61798	Vasculitis	68	Male	Training/Discovery Phase
61799	Vasculitis	68	Male	Training/Discovery Phase
61805	Vasculitis	68	Male	Training/Discovery Phase
61824	Vasculitis	69	Male	Training/Discovery Phase
61841	Vasculitis	69	Male	Training/Discovery Phase
62104	Vasculitis	69	Male	Training/Discovery Phase
9617	Vasculitis	70	Female	Training/Discovery Phase
61775	Vasculitis	70	Female	Training/Discovery Phase
62120	Vasculitis	70	Female	Training/Discovery Phase
61836	Vasculitis	70	Male	Training/Discovery Phase
61748	Vasculitis	71	Female	Training/Discovery Phase
61749	Vasculitis	75	Male	Training/Discovery Phase
36485	Arteriosclerosis	64	Male	Test/Validation Phase
62128	Arteriosclerosis	69	Male	Test/Validation Phase
20073	IgA nephropathy	16	Female	Test/Validation Phase
49496	IgA nephropathy	19	Male	Test/Validation Phase
20070	IgA nephropathy	23	Female	Test/Validation Phase
9387	IgA nephropathy	24	Female	Test/Validation Phase
20050	IgA nephropathy	25	Male	Test/Validation Phase
49514	IgA nephropathy	26	Female	Test/Validation Phase
49486	IgA nephropathy	28	Female	Test/Validation Phase
22782	IgA nephropathy	30	Male	Test/Validation Phase
22927	IgA nephropathy	30	Male	Test/Validation Phase
49499	IgA nephropathy	31	Female	Test/Validation Phase
49498	IgA nephropathy	34	Male	Test/Validation Phase
49536	IgA nephropathy	34	Male	Test/Validation Phase
22781	IgA nephropathy	36	Female	Test/Validation Phase
49483	IgA nephropathy	38	Female	Test/Validation Phase
27851	IgA nephropathy	43	Female	Test/Validation Phase
27822	IgA nephropathy	44	Male	Test/Validation Phase
22951	IgA nephropathy	46	Male	Test/Validation Phase
49545	IgA nephropathy	48	Male	Test/Validation Phase
49490	IgA nephropathy	50	Female	Test/Validation Phase
49489	IgA nephropathy	52	Male	Test/Validation Phase
49491	IgA nephropathy	52	Male	Test/Validation Phase
49517	IgA nephropathy	55	Female	Test/Validation Phase
27877	IgA nephropathy	56	Female	Test/Validation Phase
27821	IgA nephropathy	57	Male	Test/Validation Phase
27801	IgA nephropathy	62	Female	Test/Validation Phase
63648	Rheumatoid arthritis	33	Male	Test/Validation Phase
63633	Rheumatoid arthritis	34	Female	Test/Validation Phase
63661	Rheumatoid arthritis	37	Female	Test/Validation Phase
63644	Rheumatoid arthritis	39	Female	Test/Validation Phase
63655	Rheumatoid arthritis	48	Female	Test/Validation Phase

CE-MS Analysis ID	Diagnosis	Age	Gender	Analytical Phase
63657	Rheumatoid arthritis	50	Female	Test/Validation Phase
63637	Rheumatoid arthritis	52	Female	Test/Validation Phase
63668	Rheumatoid arthritis	54	Female	Test/Validation Phase
63653	Rheumatoid arthritis	57	Female	Test/Validation Phase
63643	Rheumatoid arthritis	59	Female	Test/Validation Phase
63672	Rheumatoid arthritis	63	Male	Test/Validation Phase
63652	Rheumatoid arthritis	65	Female	Test/Validation Phase
63623	Rheumatoid arthritis	67	Female	Test/Validation Phase
63629	Rheumatoid arthritis	70	Male	Test/Validation Phase
63630	Rheumatoid arthritis	73	Female	Test/Validation Phase
63645	Rheumatoid arthritis	78	Female	Test/Validation Phase
34764	Systemic lupus erythematosus	22	Female	Test/Validation Phase
31452	Systemic lupus erythematosus	26	Female	Test/Validation Phase
56649	Systemic lupus erythematosus	28	Female	Test/Validation Phase
30351	Systemic lupus erythematosus	30	Female	Test/Validation Phase
56659	Systemic lupus erythematosus	32	Female	Test/Validation Phase
28901	Systemic lupus erythematosus	35	Female	Test/Validation Phase
30349	Systemic lupus erythematosus	36	Female	Test/Validation Phase
56678	Systemic lupus erythematosus	39	Female	Test/Validation Phase
28900	Systemic lupus erythematosus	44	Male	Test/Validation Phase
30348	Systemic lupus erythematosus	47	Female	Test/Validation Phase
56664	Systemic lupus erythematosus	50	Female	Test/Validation Phase
56663	Systemic lupus erythematosus	53	Male	Test/Validation Phase
56679	Systemic lupus erythematosus	53	Male	Test/Validation Phase
56890	Systemic lupus erythematosus	61	Female	Test/Validation Phase
61826	Vasculitis	63	Female	Test/Validation Phase
61752	Vasculitis	68	Male	Test/Validation Phase
61795	Vasculitis	69	Male	Test/Validation Phase
61812	Vasculitis	70	Female	Test/Validation Phase
61834	Vasculitis	70	Male	Test/Validation Phase
61839	Vasculitis	71	Female	Test/Validation Phase

Supplementary Table S4. **Spreadsheet 8**. Independent specificity analysis. Characterization of the independent set of patients with inflammatory diseases (n=92).

CE-MS Analysis ID	Diagnosis	Age	Gender	Analytical Phase
69101	HV			Independent specificity analysis
68701	HV			Independent specificity analysis
68884	HV			Independent specificity analysis
68600	HV			Independent specificity analysis
68907	HV			Independent specificity analysis
68906	HV			Independent specificity analysis
68752	HV			Independent specificity analysis
68723	HV			Independent specificity analysis
68607	HV			Independent specificity analysis
68709	HV			Independent specificity analysis
69165	HV			Independent specificity analysis
68772	HV			Independent specificity analysis
68589	HV			Independent specificity analysis
68602	HV			Independent specificity analysis
68749	HV			Independent specificity analysis
68940	HV			Independent specificity analysis
68930	HV			Independent specificity analysis
68941	HV			Independent specificity analysis
68601	HV			Independent specificity analysis
68587	HV			Independent specificity analysis
68702	HV			Independent specificity analysis
68693	HV			Independent specificity analysis
68608	HV			Independent specificity analysis
68751	HV			Independent specificity analysis
69028	HV			Independent specificity analysis
69166	HV			Independent specificity analysis
68708	HV			Independent specificity analysis
68770	HV			Independent specificity analysis
68927	HV			Independent specificity analysis
69027	HV			Independent specificity analysis
68905	HV			Independent specificity analysis
68689	HV			Independent specificity analysis
68585	HV			Independent specificity analysis
68763	HV			Independent specificity analysis
68696	HV			Independent specificity analysis
69026	HV			Independent specificity analysis
68925	HV			Independent specificity analysis
68886	HV			Independent specificity analysis
69100	HV			Independent specificity analysis
105223	HV	19	Female	Independent specificity analysis
105429	HV	22	Female	Independent specificity analysis
105428	HV	22	Male	Independent specificity analysis
105142	HV	23	Female	Independent specificity analysis
104984	HV	24	Male	Independent specificity analysis
105058	HV	25	Female	Independent specificity analysis
105427	HV	26	Female	Independent specificity analysis

CE-MS Analysis ID	Diagnosis	Age	Gender	Analytical Phase
105132	HV	27	Female	Independent specificity analysis
105138	HV	27	Female	Independent specificity analysis
105133	HV	29	Female	Independent specificity analysis
105496	HV	29	Female	Independent specificity analysis
105225	HV	31	Female	Independent specificity analysis
105224	HV	33	Female	Independent specificity analysis
104985	HV	33	Male	Independent specificity analysis
105053	HV	35	Female	Independent specificity analysis
104982	HV	35	Female	Independent specificity analysis
105139	HV	39	Female	Independent specificity analysis
105144	HV	39	Female	Independent specificity analysis
105130	HV	47	Female	Independent specificity analysis
69176	SLE			Independent specificity analysis
69034	SLE			Independent specificity analysis
68700	SLE			Independent specificity analysis
68707	SLE			Independent specificity analysis
68591	SLE			Independent specificity analysis
68691	SLE			Independent specificity analysis
68692	SLE			Independent specificity analysis
69170	SLE			Independent specificity analysis
68724	SLE			Independent specificity analysis
69102	SLE			Independent specificity analysis
69025	SLE			Independent specificity analysis
69172	SLE			Independent specificity analysis
68785	SLE			Independent specificity analysis
68887	SLE			Independent specificity analysis
68703	SLE			Independent specificity analysis
68904	SLE			Independent specificity analysis
105514	SLE	27	Female	Independent specificity analysis
105135	SLE	29	Female	Independent specificity analysis
105003	SLE	31	Female	Independent specificity analysis
105004	SLE	31	Male	Independent specificity analysis
105063	SLE	36	Female	Independent specificity analysis
105513	SLE	37	Female	Independent specificity analysis
105222	SLE	37	Female	Independent specificity analysis
105506	SLE	39	Female	Independent specificity analysis
104997	SLE	40	Female	Independent specificity analysis
105064	SLE	42	Female	Independent specificity analysis
104993	SLE	44	Female	Independent specificity analysis
105221	SLE	46	Female	Independent specificity analysis
105507	SLE	46	Female	Independent specificity analysis
105220	SLE	49	Female	Independent specificity analysis
105667	SLE	51	Female	Independent specificity analysis
105787	SLE	55	Female	Independent specificity analysis
105239	SLE	55	Male	Independent specificity analysis
105061	SLE	63	Female	Independent specificity analysis

Abbreviations: HV, Hypersensitivity Vasculitis; SLE, Systemic Lupus erytemathosus;

Supplementary Table S4. **Spreadsheet 9**. Additional validation phase. Characterization of the additional set of patients with other cancer types (n=40).

CE-MS Analysis ID	Diagnosis	Age	Gender	Analytical Phase
35492	AML	17	Male	Additional Validation Phase
44174	AML	19	Male	Additional Validation Phase
35367	AML	30	Male	Additional Validation Phase
42292	AML	32	Female	Additional Validation Phase
78337	AML	34	Male	Additional Validation Phase
44581	AML	35	Female	Additional Validation Phase
39136	AML	39	Male	Additional Validation Phase
44586	AML	48	Male	Additional Validation Phase
44599	AML	49	Male	Additional Validation Phase
44582	AML	50	Female	Additional Validation Phase
39119	AML	50	Male	Additional Validation Phase
44598	AML	50	Male	Additional Validation Phase
44597	AML	53	Female	Additional Validation Phase
48548	AML	53	Female	Additional Validation Phase
79802	AML	55	Female	Additional Validation Phase
20804	AML	56	Male	Additional Validation Phase
79801	AML	56	Male	Additional Validation Phase
44587	AML	57	Male	Additional Validation Phase
36664	AML	59	Female	Additional Validation Phase
40169	AML	59	Female	Additional Validation Phase
44584	AML	59	Female	Additional Validation Phase
78401	AML	60	Male	Additional Validation Phase
40213	AML	61	Female	Additional Validation Phase
44360	AML	61	Male	Additional Validation Phase
44600	AML	62	Female	Additional Validation Phase
44588	AML	65	Female	Additional Validation Phase
44585	AML	70	Female	Additional Validation Phase
36195	Breast cancer	39	Female	Additional Validation Phase
51708	Bronchial cancer	74	Male	Additional Validation Phase
99912	Haemangioma	33	Female	Additional Validation Phase
38622	Metastatic Breast cancer	39	Female	Additional Validation Phase
38663	Metastatic Breast cancer	39	Female	Additional Validation Phase
38644	Metastatic Breast cancer	61	Female	Additional Validation Phase
38445	Metastatic Stomach cancer	40	Male	Additional Validation Phase
38478	Metastatic Stomach cancer	59	Male	Additional Validation Phase
38447	Metastatic Colon cancer	63	Male	Additional Validation Phase
38462	Metastatic Stomach cancer	66	Female	Additional Validation Phase
99518	Neuroendocine tumor	40	Female	Additional Validation Phase
99252	Neuroendocine tumor	68	Male	Additional Validation Phase
51606	Small cell lung cancer	49	Male	Additional Validation Phase

Abbreviations: AML, Acute Myeloid Leukemia
